# The 2023 MANCTRA Acute Biliary Pancreatitis Care Bundle: A Joint Effort Between Human Knowledge and Artificial Intelligence (ChatGPT) to Optimize the Care of Patients With Acute Biliary Pancreatitis in Western Countries

**DOI:** 10.1097/SLA.0000000000006008

**Published:** 2023-07-17

**Authors:** Mauro Podda, Marcello Di Martino, Benedetto Ielpo, Fausto Catena, Federico Coccolini, Francesco Pata, Giovanni Marchegiani, Belinda De Simone, Dimitrios Damaskos, Damian Mole, Ari Leppaniemi, Massimo Sartelli, Baohong Yang, Luca Ansaloni, Walter Biffl, Yoram Kluger, Ernest E. Moore, Gianluca Pellino, Salomone Di Saverio, Adolfo Pisanu

**Affiliations:** * Department of Surgical Science, Emergency Surgery Unit, Cagliari State University Hospital, Cagliari, Italy; † Division of Hepatobiliary and Liver Transplantation Surgery, A.O.R.N. Cardarelli, Naples, Italy; ‡ Hepatobiliary Division, Hospital del Mar, Pompeu Fabra University, Barcelona, Spain; § Department of Emergency and Trauma Surgery, Bufalini Hospital, Cesena, Italy; ∥ General, Emergency and Trauma Surgery Unit, Pisa University Hospital, Pisa, Italy; ¶ Department of Surgery, University of Calabria, Cosenza, Italy; # Department of Surgical, Oncological and Gastroenterological Sciences (DISCOG), Hepato-Pancreato-Biliary Surgery and Liver Transplantation Unit, University of Padua, Padua, Italy; ** Department of Emergency and Metabolic Minimally Invasive Surgery, Centre Hospitalier Intercommunal de Poissy/Saint Germain en Laye, Poissy Cedex, France; †† Department of Upper GI Surgery, Royal Infirmary of Edinburgh, Edinburgh, Scotland, UK; ‡‡ Centre for Inflammation Research, Clinical Surgery, University of Edinburgh, Edinburgh, Scotland, UK; §§ Department of Abdominal Surgery, University of Helsinki and Helsinki University Central Hospital, Helsinki, Finland; ∥∥ Department of Surgery, Macerata Civil Hospital, Macerata, Italy; ¶¶ Department of Oncology, Weifang People’s Hospital, The First Affiliated Hospital of Weifang Medical University, Weifang, Shandong, China; ## Department of Gastroenterology, The First Affiliated Hospital of Zhengzhou University, Zhengzhou, Henan, China; *** Department of General Surgery, IRCCS Policlinico San Matteo Foundation, Pavia, Italy; ††† Division of Trauma and Acute Care Surgery, Scripps Memorial Hospital La Jolla, La Jolla, CA; ‡‡‡ Department of General Surgery, Rambam Medical Center, Haifa, Israel; §§§ Denver Health System—Denver Health Medical Center, Denver, CO; ∥∥∥ “Luigi Vanvitelli” University of Campania, Naples, Italy; ¶¶¶ Department of Colorectal Surgery, Vall d’Hebron University Hospital, Universitat Autonoma de Barcelona UAB, Barcelona, Spain; ### Department of Surgery, Madonna del Soccorso Hospital, San Benedetto del Tronto, Italy

**Keywords:** acute biliary pancreatitis, artificial intelligence, care bundle, ChatGPT, clinical indicators, GRADE, guidelines

## Abstract

**Objective:**

To generate an up-to-date bundle to manage acute biliary pancreatitis using an evidence-based, artificial intelligence (AI)-assisted GRADE method.

**Background:**

A care bundle is a set of core elements of care that are distilled from the most solid evidence-based practice guidelines and recommendations.

**Methods:**

The research questions were addressed in this bundle following the PICO criteria. The working group summarized the effects of interventions with the strength of recommendation and quality of evidence applying the GRADE methodology. ChatGPT AI system was used to independently assess the quality of evidence of each element in the bundle, together with the strength of the recommendations.

**Results:**

The 7 elements of the bundle discourage antibiotic prophylaxis in patients with acute biliary pancreatitis, support the use of a full-solid diet in patients with mild to moderately severe acute biliary pancreatitis, and recommend early enteral nutrition in patients unable to feed by mouth. The bundle states that endoscopic retrograde cholangiopancreatography should be performed within the first 48 to 72 hours of hospital admission in patients with cholangitis. Early laparoscopic cholecystectomy should be performed in patients with mild acute biliary pancreatitis. When operative intervention is needed for necrotizing pancreatitis, this should start with the endoscopic step-up approach.

**Conclusions:**

We have developed a new care bundle with 7 key elements for managing patients with acute biliary pancreatitis. This new bundle, whose scientific strength has been increased thanks to the alliance between human knowledge and AI from the new ChatGPT software, should be introduced to emergency departments, wards, and intensive care units.

Acute pancreatitis is an inflammatory disease with different severity patterns and an incidence ranging from 5 to 30 cases per 100,000 inhabitants/year.^1^ It is still associated with overall mortality rates reaching 2% in Western countries^2^ and 7.5% in Asia.^3^ In 80% of cases, the outcome of acute pancreatitis is rapidly favorable. However, necrotizing pancreatitis may develop in up to 20% of cases, with associated significant rates of organ failure (38%) and deaths (15%).^4^

Although clinical practice guidelines for managing acute pancreatitis have been developed and disseminated by multiple scientific bodies,^5–7^ the adoption of evidence-based recommendations for acute biliary pancreatitis remains suboptimal. Reports about the real-world implementation of evidence-based statements demonstrated that the publication of nationally or internationally developed and approved recommendations alone is insufficient to modify clinical practice.^8,9^ Moreover, although it is commonly believed that noncompliance with published guidelines indicates areas in which recommendations are based on insufficient evidence, previous studies have shown a lack of compliance in areas where randomized controlled trials (RCTs) have already resolved controversial issues during the last 10 years.^10,11^ In 2022, we conducted an international cohort study that assessed the compliance rate with current guidelines in the treatment of 5275 patients with acute biliary pancreatitis, which showed overall poor compliance with evidence-based guidelines and wide variability of practice based on the admitting specialty.^11^ The most commonly reported deviations between clinical practice and guidelines included the indications for antibiotics, the need and also the characteristics and timing of artificial nutritional support, as well as the surgical/endoscopic management with endoscopic retrograde cholangiopancreatography (ERCP) and early cholecystectomy.

Guidelines must be widely disseminated to be implemented in daily clinical practice and improve patients’ prognosis. An effective dissemination strategy can be the simultaneous implementation of multiple measures, known as a “care bundle.” The Institute for Healthcare Improvement^12^ defined a care bundle as a straightforward set of core elements of care that are distilled from the most solid evidence-based practice guidelines recommendations and that, when implemented as a group of recommendations, have a positive effect on outcomes beyond those achieved when the individual elements are implemented alone. Lessons learned from the implementation of sepsis bundles,^13,14^ ventilator bundles,^15^ bundles for the prevention of surgical-site infections,^16^ and central line bundles^17,18^ showed that the improvement in patients’ prognosis is more remarkable when a core bundle has been implemented than when individual interventions have been delivered separately.^19^ To date, bundles that promote mandatory items or procedures to be implemented in clinical practice for acute biliary pancreatitis have rarely been used, and exclusively in Eastern countries.^7^ In the nationwide epidemiological analysis of the clinical practice of acute pancreatitis in Japan, Masamune et al^20^ found that the case-fatality rate was significantly lower when at least 8 elements of the bundle were implemented than when <8 items were followed (1.0% vs 7.1%).

With this in mind, this document aims to emphasize the importance of acute biliary pancreatitis bundle elements in Western Countries for the first time. The effectiveness and outcomes of the bundle will then be assessed in 2025 with a prospective study conducted within the centers that participated in the “coMpliAnce with evideNce-based cliniCal guidelines in the managemenT of acute biliaRy pancreAtitis” (MANCTRA-1) project.^21^

## METHODS

During the development of the care bundle, the members of the MANCTRA-1 Steering Committee extensively revised current guidelines with the aim of identifying the most solid evidence-based practices surgeons and physicians worldwide treating acute biliary pancreatitis can implement to achieve better medical care. Guidelines recommendations expected to yield favorable clinical results or increase the cost-effectiveness of diagnostic and therapeutic procedures were included in the initial assessment. In addition, the focus was set on suboptimal or scarce compliance to guidelines items raised by the results of the MANCTRA-1 international study,^11^ where compliance was determined by comparing the collected patient-based data with selected recommendations from 5 current evidence-based guidelines^5,6,22–24^ (Table 1).

**TABLE 1:** Summary of the MANCTRA-1 Audit^*^

	Investigated item	Compliance level (%)	Population
1	Optimal timing for the index CE-CT assessment is 72–96 hours after the onset of symptoms	6.1	Patients with severe acute biliary pancreatitis
2	Routine prophylactic antibiotics are not recommended for all patients with acute biliary pancreatitis (patients on antibiotics)	55.8 53.4 83.4 85.2	General cohort of patients with acute biliary pancreatitis Patients with mild acute biliary pancreatitis Patients with severe acute biliary pancreatitis Patients with infected pancreatic necrosis
3	Serum measurements of procalcitonin (PCT) may be valuable in predicting the risk of developing infected pancreatic necrosis	30.8 29.6	Patients with severe acute biliary pancreatitis Patients with infected pancreatic necrosis
4	Early (within 24 hours) oral feeding as tolerated, rather than keeping the patient nil per os, is recommended in patients with acute biliary pancreatitis	44.7 47.7	General cohort of patients with acute biliary pancreatitis Patients with mild acute biliary pancreatitis
5	EN is recommended to prevent gut failure and infectious complications in patients with acute biliary pancreatitis and inability to feed orally	33.2 39.3	Patients with severe acute biliary pancreatitis Patients with infected pancreatic necrosis
6	TPN should be avoided (patients on TPN)	36.2 34.4	Patients with severe acute biliary pancreatitis Patients with infected pancreatic necrosis
7	Early ERCP/ES should be performed in gallstone-induced acute biliary pancreatitis when complications of cholangitis and CBD obstruction occur	46.0 60.1 56.7	Patients with cholangitis Patients with CBD obstruction Patients with cholangitis and CBD obstruction
**8**	In patients with acute necrotizing pancreatitis, percutaneous or endoscopic drainage as the first-line treatment (step-up approach) delays the surgical treatment to a more favorable time or even results in complete resolution of infection in 25%–60% of patients, and it is recommended as the first line of treatment	33.7	Patients with acute necrotizing pancreatitis or infected pancreatic necrosis
**9**	Therapeutic intervention for infected pancreatic necrosis should be performed after 4 weeks of onset when the necrosis has been sufficiently walled off	37.2	Patients with infected pancreatic necrosis
**10**	Laparoscopic cholecystectomy during the index admission, rather than after discharge, is recommended in mild acute biliary pancreatitis	29.0	Patients with mild acute biliary pancreatitis

* Podda et al. *Pancreatology*. 2022;22(7):902-916.^11^

CBD indicates common bile duct; CE-CT, contrast enhanced CT scan; EN, enteral nutrition; ERCP/ES, endoscopic retrograde cholangiopancreatography/endoscopic sphincterotomy; PCT, procalcitonin; TPN, total parenteral nutrition.

### Objectives of the MANCTRA Acute Biliary Pancreatitis Care Bundle

The primary investigators (M.P. and A.P.) and the Steering Committee (B.I., G.P., F.P., M.D.M., and S.D.S.) of the MANCTRA project identified researchers with nationally and internationally recognized experience in caring for patients with acute biliary pancreatitis to participate in the panel of experts.

The bundle is intended for all health care professionals involved in the treatment of adult patients with acute biliary pancreatitis, with the aim to be a tool to improve and standardize the clinical practice concerning the diagnosis and treatment, offer the patient the opportunity to take advantage of optimized therapeutic paths based on scientific evidence and offer a reference basis on the available evidence.

### PICO Question Development

The panel formulated the research questions addressed in this bundle following the PICO criteria (Population, Intervention, Comparison, and Outcome). Several outcomes of interest for each question were identified, and their relative relevance was graded as follows:^25,26^

- Essential outcomes (also referred to as “critical”).
- Important but not essential outcomes.
- Irrelevant outcomes.

Only “critical” or “important” outcomes were considered in the literature review and subsequently in the formulation of the bundle.

### Literature Review

A systematic literature search was conducted (Supplemental Digital Content Appendix 1, http://links.lww.com/SLA/E734) to identify the best evidence supporting the recommendations of the guidelines and to reassess when needed, the quality of the evidence and the strength of the recommendation behind every single element. Systematic reviews with or without meta-analyses, RCT, and observational studies (n-RCT) supporting recommendations of current acute biliary pancreatitis guidelines were searched using Medline (through PubMed), the Cochrane Library, and Embase. Searches were limited to articles published in English from January 2000 through February 2023. For Cochrane reviews with multiple revisions or editions, we only included the most updated version. Case reports, trial protocols, narrative reviews and summaries, letters, editorials, position papers, congress abstracts, and posters were excluded. The list of titles and abstracts obtained from querying the databases was screened on Rayyan (https://www.rayyan.ai/) to select the relevant articles for each question. Then, 2 members of the Working Group (M.P. and A.P.) examined each study separately, determining its inclusion or exclusion based on pre-established criteria. In the absence of systematic reviews of RCTs and n-RCTs, or in the presence of systematic reviews judged to be of low methodological quality according to the AMSTAR II tool,^27^ primary studies were analyzed. For each outcome considered in the clinical questions, the working group assessed the confidence in effect estimates based on 5 dimensions (risk of bias, inconsistency, indirectness, imprecision of the estimated effects, and publication bias).^28–34^ For the aim of this study, the definitions adopted for “mild acute pancreatitis,” “severe acute pancreatitis,” “predicted severe acute pancreatitis,” “acute cholangitis,” “pancreatic necrosis,” and “infected pancreatic necrosis” are reported in Supplemental File (Supplemental Digital Content Table 1, http://links.lww.com/SLA/E734).

### Synthesis of the Evidence and Development of the Recommendations

The working group summarized the efficacy and safety of the interventions in synoptic tables reporting the study’s general characteristics and the summary of the effects with the strength of recommendation (SoR) and quality of evidence (QoE) applying the GRADE methodology,^35–37^ which provides the overall assessment of the relationship between desirable and undesirable effects through the “Evidence to Decision Framework”^38,39^ (Supplemental Digital Content Figs. 1–10, http://links.lww.com/SLA/E734; Supplemental Digital Content Tables 4–12, http://links.lww.com/SLA/E734). Furthermore, the ChatGPT artificial intelligence (AI) system (https://chat.openai.com/chat) was used to independently assess the QoE of each element in the bundle, together with the strength of the recommendations. ChatGPT is a large language model developed by OpenAI based on the “Generative Pretrained Transformer” architecture. It can generate human-like responses to natural language prompts, thanks to its ability to understand and model the patterns and structure of language. ChatGPT can provide information on a wide range of scientific topics and answer questions related to various research fields, including physics, biology, chemistry, mathematics, and medicine, providing definitions and explanations of scientific concepts and terms, describing scientific theories and principles, offering insights into current research and developments in the scientific community, and suggesting potential avenues for further exploration or study in a particular field of science.

The final recommendations were suggested in a preliminary version by the working group and were then discussed by the panel.

### Development of the Bundle

**FIGURE 1:**
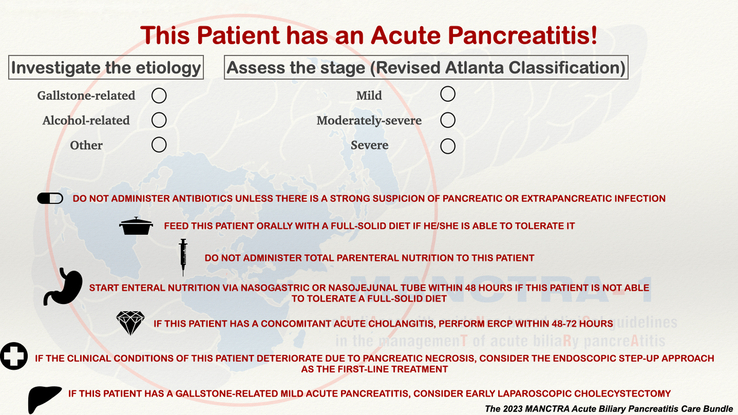
The 2023 MANCTRA Acute Biliary Pancreatitis Care Bundle.

Only elements with moderate to high-quality evidence and strong recommendation based on the literature review were chosen to enter the 2023 MANCTRA Acute Biliary Pancreatitis Care Bundle. We suggest that for future implementation of the present, except in special situations, all the elements are accomplished and recorded in medical records through an easy-to-use checklist (Fig. 1). The checklist was developed to be considered for placement by the bedside in the emergency department, intensive care units (ICUs), and wards.

### Diffusion, Implementation, and Assessment of the Bundle

**FIGURE 2:**
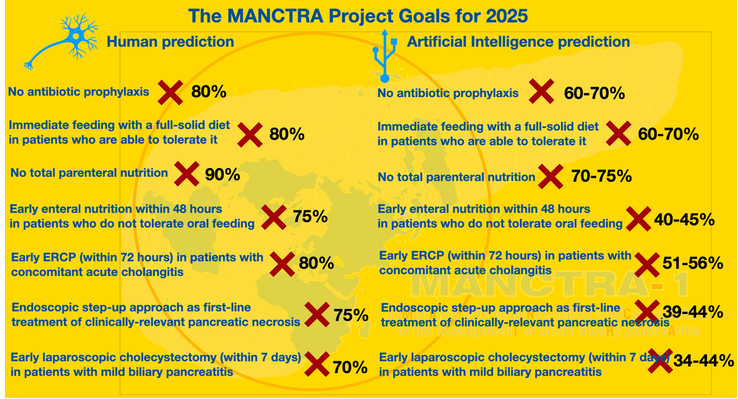
The MANCTRA project goals for 2025.

The monitoring of the application of the elements can be carried out considering a benchmark of ≥50% for the whole bundle and specific thresholds for every single element. The working group and the panel of experts have also elaborated forecasts on compliance with every single element of the bundle based on the potential for its dissemination and penetration into the clinical care practice (Fig. 2). For each bundle element, the panel of experts performed a literature review to highlight the levels of compliance reported in previous studies. The average compliance rate was taken as a reference, and +10% was applied to the final goal as a likely increase thanks to human effort. Within this context, the forecasts made by the panel have considered that an implementation plan drawn up by human intelligence can consider the organizational and behavioral efforts to achieve the goal, an aspect that AI cannot yet evaluate. These estimates were subsequently submitted for consensus by the experts until general approval by the same was reached. These predictions were compared with those elaborated by the ChatGPT AI model, which instead based its forecasts on the analysis of the trends in the literature.

## RESULTS

The synthesis of every bundle element has been classified into recommendations with a strength (SoR) based on the quality of literature evidence (QoE) (Supplemental Digital Content Table 2, http://links.lww.com/SLA/E734).

Research question 1. *Should routine enhanced computed tomography (CT) scan at the time of hospital admission versus contrast-enhanced CT performed 72 to 96 hours after onset of symptoms be used for the diagnosis of local complications (fluid collections, pancreatic necrosis) in patients with severe acute biliary pancreatitis?*

We analyzed 14 articles concerning the optimal timing for performing abdomen CT in patients with severe acute biliary pancreatitis, including 12 n-RCTs and 2 RCTs. The synthesis of evidence from the analyzed studies (Supplemental Digital Content Fig. 1, http://links.lww.com/SLA/E734) demonstrated that performing CT of the abdomen within 72 hours of hospital admission can predict the risk of local pancreatic complications, including pancreatic and peripancreatic necrosis (7 n-RCTs, moderate certainty). The presence of areas with reduced parenchymal enhancement on early CT may be associated with the development of organ failure and other severe systemic complications (3 n-RCTs, low certainty). Although it has been reported that the presence of areas of the pancreas with decreased enhancement at the level of the pancreatic head and tail on an early CT scan may be associated with an increased risk of mortality (2 RCTs, high certainty), this does not lead to an increase in the rate of interventional treatments (3 n-RCTS, very low certainty). An early CT scan is able to diagnose the presence of peripancreatic fluid layers and areas of pancreatic necrosis, and it can play a decisive role when considering whether or not to perform an early cholecystectomy. In this sense, patients with mild acute biliary pancreatitis who do not show peripancreatic fluid can safely undergo early cholecystectomy without waiting for the complete resolution of the acute inflammation (1 RCT, moderate certainty).

Response: The experts did not rate the certainty of the evidence obtained from the analysis of this research question high enough to produce a bundle element (MANCTRA GRADE assessment: QoE, low; SoR, weak). CT at the time of hospital admission may be useful for identifying local complications, such as fluid collections and pancreatic necrosis, whereas others suggest that a delay of 72 to 96 hours may be more appropriate to allow time for the development of these complications. The decision of when to perform imaging and what type of imaging to use should be individualized based on the patient’s clinical condition and the availability of resources (ChatGPT GRADE assessment: QoE, low; SoR, weak).

Research question 2. *Should routine prophylactic antibiotics versus no routine prophylactic antibiotics be used for patients with acute biliary pancreatitis in the absence of infectious complications?*

Twelve articles were analyzed, of which 7 were systematic reviews with meta-analyses of RCTs, 4 were systematic reviews and meta-analyses of RCTs and n-RCTs, and 1 RCT.

The synthesis of the evidence deriving from the studies taken into consideration (Supplemental Digital Content Fig. 2, http://links.lww.com/SLA/E734) showed that the use of antibiotic prophylaxis does not significantly reduce the incidence of infected pancreatic necrosis (11 RCTs, high certainty), the mortality rate due to superinfection of pancreatic necrosis (10 RCTs, high certainty), and the need for endoscopic or surgical step-up approaches or upfront surgical necrosectomy (7 RCTs, high certainty). However, a body of low-certainty evidence has suggested that prophylactic antibiotics may play a role in reducing the rate of extrapancreatic infections (8 RCTs, low certainty) and reducing the length of hospital stay (1 RCT, low certainty).

Response: Antibiotic prophylaxis is strongly discouraged in patients with acute biliary pancreatitis of any degree unless there is a strong suspicion of an active pancreatic or extrapancreatic infection (MANCTRA GRADE assessment: QoE, high; SoR, strong). The evidence suggests that the routine use of antibiotic prophylaxis in patients with acute biliary pancreatitis is not recommended unless there is a strong suspicion of an active pancreatic or extrapancreatic infection (ChatGPT GRADE assessment: QoE, high; SoR, strong).

Research question 3. *Should serum measurements of procalcitonin (PCT) versus other sepsis markers be used for the early diagnosis of infected pancreatic necrosis in patients with severe acute biliary pancreatitis?*

Seventeen articles were analyzed, including 4 systematic reviews and meta-analyses of n-RCTs, 10 n-RCTs, 2 RCTs, and 1 study with an undefined design. Evidence synthesis from the studies reviewed (Supplemental Digital Content Fig. 3, http://links.lww.com/SLA/E734) demonstrated that a baseline PCT value at admission >1.0 ng/mL is associated with adverse outcomes. PCT dosage is superior to c-reactive protein and Interleukin 6 in predicting acute renal failure (7 n-RCTs, moderate certainty) and severe acute biliary pancreatitis (18 n-RCTs, low certainty).

PCT may help predict the need for Intensive Care Unit (ICU) admission (2 n-RCTs, moderate certainty) and mortality (4 n-RCTs, low certainty). The most significant utility of baseline and serial PCT dosage in patients with severe acute biliary pancreatitis lies in the possibility of modulating antibiotic therapy and its duration (2 RCTs, high certainty).

Response: The experts did not rate the certainty of the evidence obtained from the analysis of this research question high enough to produce a bundle item (MANCTRA GRADE assessment: QoE, moderate; SoR, weak). The use of serial PCT measurements in the context of diagnostic and therapeutic pathways of patients with severe acute biliary pancreatitis and infected pancreatic necrosis seems to be supported by the evidence. However, the optimal timing and frequency of PCT measurements, as well as the cutoff values for diagnosing infected pancreatic necrosis and predicting treatment response, are not yet well established (ChatGPT GRADE assessment: QoE, moderate to low; SoR, weak).

Research question 4. *Should early (within 24 hours) oral feeding as tolerated versus keeping the patient nil per os be used for patients with mild acute biliary pancreatitis (if tolerated)?*

Seventeen articles were analyzed, including 3 systematic reviews and meta-analyses of RCTs, 1 systematic review and meta-analysis of RCTs and n-RCTs, 1 systematic review and meta-analysis of n-RCTs, and 12 RCTs. The synthesis of evidence (Supplemental Digital Content Fig. 4, http://links.lww.com/SLA/E734) showed that an early (within 24 hours) oral solid diet, if tolerated, compared with nil by mouth in patients with mild acute biliary pancreatitis, reduces the hospital stay (10 RCTs, high certainty) and accelerates patient recovery, without affecting the rate of gastrointestinal adverse events and pain recovery (7 RCTs, high certainty). Immediate oral refeeding with a solid diet does not impact mortality (5 RCTs, moderate certainty) and overall complication rates (4 RCTs, moderate certainty). Furthermore, early oral feeding with a solid diet is not associated with the progression of acute biliary pancreatitis (3 RCTs, high certainty). Serum lipase levels represent an accurate parameter for identifying those patients at risk of intolerance to early oral refeeding (9 n-RCTs, moderate certainty).

Response: Patients with mild acute biliary pancreatitis, who are able to tolerate it, should be fed a full-solid diet instead of nil per os (MANCTRA GRADE assessment: QoE, high; SoR, strong). The evidence supports the use of solid diets in patients with mild to moderately severe acute biliary pancreatitis who can tolerate it (ChatGPT GRADE assessment: QoE, moderate to high; SoR, strong).

Research question 5. *Should enteral nutrition (EN) versus total parenteral nutrition (TPN) be used for the prevention of gut failure and infectious complications in patients with acute biliary pancreatitis and the inability to feed orally?*

Twenty-one articles were analyzed, including 10 systematic reviews and meta-analyses of RCTs and 11 RCTs. The synthesis of the evidence (Supplemental Digital Content Fig. 5, http://links.lww.com/SLA/E734) showed that in patients with severe acute biliary pancreatitis (or predicted severe acute biliary pancreatitis), EN through the nasogastric or nasojejunal routes is effective in reducing the rate of overall infections (11 RCTs, high certainty), the onset of multiple organ failure (9 RCTs, moderate certainty), the mean length of hospital stay (11 RCTs, high certainty), the incidence of overall complications (11 RCTs, high certainty) and the mortality rate (10 RCTs, high certainty) compared with total TPN. Similarly, compared with TPN, EN can reduce the incidence of infected pancreatic necrosis (8 RCTs, high certainty) and catheter-related septic complications (12 RCTs, high certainty). Furthermore, EN is associated with better glycemic control (6 RCTs, high certainty) and less need for interventional or surgical procedures for pancreatic necrosis (5 RCTs, moderate certainty). With lower certainty of the evidence, EN is associated with better maintenance of the gut barrier (3 RCTs, low certainty) and a reduction in hospital management costs (2 RCTs, low certainty) compared with TPN.

Response: EN should be started in patients with severe acute biliary pancreatitis unable to feed by mouth. TPN should only be reserved for those patients in whom it is impossible to start EN (MANCTRA GRADE assessment: QoE, high; SoR, strong). The available evidence consistently supports the use of EN as the preferred method of nutritional support in patients with severe acute biliary pancreatitis, and the potential risks associated with TPN support its limited use (ChatGPT GRADE assessment: QoE, high; SoR, strong).

Research question 6. *Should early EN (e-EN) within 48 hours versus delayed EN beyond 48 hours be used in patients with severe acute biliary pancreatitis and inability to feed orally?*

Ten articles were analyzed, including 2 systematic reviews and meta-analyses of RCTs, 3 systematic reviews and meta-analyses of RCTs and n-RCTs, and five RCTs.

The synthesis of the evidence (Supplemental Digital Content Fig. 6, http://links.lww.com/SLA/E734) demonstrated that in patients with severe acute biliary pancreatitis (or predicted severe acute biliary pancreatitis), e-EN established within 48 hours of admission is more effective than TPN in reducing the mortality rate (10 RCTs, high certainty), the incidence of new-onset organ failure (8 RCTs, high certainty), and the length of hospital stay (2 RCTs, high certainty). e-EN is also associated with reduced rates of infection (8 RCTs, moderate certainty), catheter-related complications (6 n-RCTs, high certainty), infected pancreatic necrosis (5 RCTs, moderate certainty), and hyperglycemia (4 n-RCTs, moderate certainty). In addition, e-EN established within 48 hours of admission proved to be more effective than TPN and delayed EN (>48 hours) in reducing the need for operative interventions (7 RCTs, moderate certainty) and in reducing systemic inflammatory response syndrome (4 RCTs, low certainty).

Response: e-EN should be established within 48 hours of admission through nasojejunal or nasogastric routes for patients with severe acute biliary pancreatitis and inability to feed orally (MANCTRA GRADE assessment: QoE, high; SoR strong). The available evidence consistently supports using e-EN through nasojejunal or nasogastric routes within 48 hours of admission in patients with severe acute biliary pancreatitis who cannot feed orally (ChatGPT GRADE assessment: QoE, high; SoR, strong).

Research question 7. *Should early (within 48–-72 hours) ERCP/ES versus delayed (>72 hours) or conservative treatment be used in acute biliary pancreatitis when cholangitis and/or common bile duct obstruction occur?*

Fifteen studies were analyzed, including 8 systematic reviews and meta-analyses of RCTs, 3 systematic reviews and meta-analyses of n-RCTs, 1 systematic review and meta-analysis of RCTs and n-RCTs, and 3 n-RCTs. The synthesis of evidence (Supplemental Digital Content Fig. 7, http://links.lww.com/SLA/E734) showed that ERCP within 48 to 72 hours of admission is associated with lower in-hospital mortality than delayed ERCP in patients with acute biliary pancreatitis and acute cholangitis. In contrast, early ERCP in patients without cholangitis does not reduce mortality. Early ERCP should not be performed unless there is at least a slight suspicion of cholangitis or persistent ampullary obstruction (9 RCTs, high certainty). ERCP within 48 to 72 hours of admission is associated with a shorter hospital stay (4 RCTs, moderate certainty). Performing ERCP within 48 to 72 hours of hospitalization in patients with acute biliary pancreatitis and cholangitis is associated with a reduced risk of new organ failure and overall complications (6 RCTs, high certainty) and infected pancreatic necrosis (9 RCTs, high certainty). A higher rate of ICU admissions is observed in patients with severe acute biliary pancreatitis who underwent ERCP within 48 hours. The evidence in this sense comes from n-RCTs and may have been influenced by selection bias (3 n-RCTs, low certainty). In patients with acute biliary pancreatitis and cholangitis, early ERCP performed within the first 48 to 72 hours reduces the rate of local adverse events (9 RCTs, high certainty) and reduces pain duration and fever (2 n-RCTs, moderate certainty). In contrast, no benefit was reported in favor of early ERCP in patients without cholangitis (10 RCTs, low certainty).

Response: ERCP should be performed within the first 48 to 72 hours of hospital admission in patients with acute biliary pancreatitis and concomitant cholangitis (MANCTRA GRADE assessment: QoE, high; SoR, strong). The available evidence consistently supports the use of early ERCP within 48 to 72 hours of hospital admission in patients with acute biliary pancreatitis and concomitant cholangitis (ChatGPT GRADE assessment: QoE, moderate to high; SoR, strong).

Research question 8. *Should surgical or endoscopic step-up approach versus upfront necrosectomy be used as the first line of treatment for patients with infected pancreatic necrosis?*

Nineteen studies were analyzed, including 4 systematic reviews and meta-analyses of RCTs, 1 systematic review and meta-analysis of n-RCTs, 4 systematic reviews and meta-analyses of RCTs and n-RCTs, 8 RCTs, and 2 n-RCTs. The synthesis of evidence (Supplemental Digital Content Fig. 8, http://links.lww.com/SLA/E734) demonstrated that the minimally invasive surgical step-up approach and the endoscopic step-up approach are associated with a lower incidence of adverse events, serious adverse events, organ failure, and lower hospital costs compared with open surgical necrosectomy in clinically deteriorating patients with acute necrotizing pancreatitis, associated or not with necrosis infection.

In terms of the clinical success of the procedure, the endoscopic step-up approach does not seem to be superior to the surgical step-up approach. However, there is moderate evidence that, in patients treated with the endoscopic step-up approach, the risk of pancreatic fistula is lower than with the surgical step-up approach (4 RCTs, moderate certainty). Regarding in-hospital mortality, a network meta-analysis published in 2021, and subsequent RCTs suggest that the first interventional approach in case of suspected infected pancreatic necrosis should be the endoscopic step-up approach. Open surgical necrosectomy, if necessary, should be delayed as long as possible (8 RCTs, low certainty). Regarding in-hospital morbidity, the endoscopic step-up approach is associated with the highest probability of being the safest approach (8 RCTs, moderate certainty). Similarly, the step-up endoscopic approach is the operative intervention correlated with the highest probability of shortening hospital stay (7 RCTs, moderate certainty) and ICU stay (3 RCTs, low certainty), followed by the surgical step-up approach. The endoscopic step-up approach is associated with a lower incidence of new-onset organ failure than surgical necrosectomy (4 RCTs, moderate certainty). The incidence of postoperative pancreatic fistula is lower in patients treated with the endoscopic step-up approach than in those treated with the surgical step-up approach (8 RCTs, moderate certainty). In contrast, the incidence of pancreatic insufficiency at long-term follow-up (6 months) is not different comparing the two techniques (5 RCTs, low certainty). Furthermore, patients treated with the endoscopic-type step-up approach may require fewer re-operations for necrosis recurrence at the initial 6-month follow-up (7 n-RCTs, low certainty).

Response: In clinically deteriorating patients with acute necrotizing pancreatitis, associated or not with necrosis infection, the first interventional therapeutic approach should be the endoscopic step-up approach. The minimally invasive surgical step-up approach can be considered the alternative choice (MANCTRA GRADE assessment: QoE, high; SoR, strong). The available evidence consistently supports the use of the endoscopic step-up approach as the first interventional therapeutic approach in clinically deteriorating patients with acute necrotizing pancreatitis, associated or not with necrosis infection (ChatGPT GRADE assessment: QoE, moderate to high; SoR, strong).

Research question 9. *Should delayed (after 4 weeks) therapeutic interventions (endoscopic or surgical step-up approach, surgical necrosectomy) versus early interventions be used for patients with necrotizing pancreatitis who remain clinically stable?*

Twelve studies were analyzed, including 2 systematic reviews and meta-analyses of RCTs, 1 systematic review and meta-analysis of RCTs and n-RCTs, 1 systematic review and meta-analysis of n-RCTs, 2 RCTs, and 6 n-RCTs. The synthesis of evidence (Supplemental Digital Content Fig. 9, http://links.lww.com/SLA/E734) showed that early endoscopic and surgical step-up approaches and open surgery are associated with higher mortality rates (8 RCTs, high certainty) and overall postprocedure morbidity (5 RCTs, moderate certainty) than delayed interventional strategies. In addition, early interventions (<4 weeks) for necrotizing pancreatitis are associated with an increased incidence of gastrointestinal fistulas or perforations compared with delayed interventions (8 RCTs, high certainty). Furthermore, early interventions for necrotizing pancreatitis do not improve clinical outcomes (5 RCTs, moderate certainty) and are associated with significantly more extended hospital stays than interventions delayed beyond 4 weeks (7 RCTs, moderate certainty). Invasive interventions delayed beyond 4 weeks for necrotizing pancreatitis, particularly endoscopic step-up approaches, are associated with reduced length of ICU stay (3 RCTs, moderate certainty) and pancreatic fistula rates (6 RCTs, high certainty) compared with operations performed before the demarcation of the necrosis (conventionally 4 weeks). Patients treated with early interventions for necrotizing pancreatitis tend to be complicated by new onset organ failure compared with patients for whom invasive interventions are delayed until necrosis is circumscribed (4 n-RCTs, moderate certainty), and those treated with a delayed strategy need fewer rescue necrosectomy reoperations during follow-up (5 n-RCTs, moderate certainty).

Response: In patients with acute necrotizing pancreatitis, since the timing of the operation is a risk factor for mortality and major complications, if the patient’s clinical conditions allow, any interventional strategy (preferably endoscopic or minimally invasive step-up approach) should be delayed beyond 4 weeks (MANCTRA panel GRADE assessment: QoE, high; SoR, strong). Delayed intervention beyond 4 weeks may be beneficial in patients with necrotizing pancreatitis, particularly if the patient’s clinical conditions allow. However, it is important to note that the evidence is not consistent, and the optimal timing of intervention may depend on various factors, such as the severity of the disease and the presence of infection (ChatGPT GRADE assessment: QoE low to moderate, SoR weak).

Research question 10. *Should early laparoscopic cholecystectomy during index admission (or within 14 days) versus delayed laparoscopic cholecystectomy after hospital discharge be used for patients with mild acute biliary pancreatitis?*

Nine studies were analyzed, including 3 systematic reviews and meta-analyses of RCTs, 2 systematic reviews and meta-analyses of RCTs and n-RCTs, and 4 RCTs. The synthesis of the evidence (Supplemental Digital Content Fig. 10, http://links.lww.com/SLA/E734) showed that early laparoscopic cholecystectomy (performed during the same hospitalization or within 14 days of the acute episode) after mild acute biliary pancreatitis reduces the readmission rate for recurrent biliary complications (6 RCTs, high certainty). Recent RCTs comparing different time intervals have concluded that in mild cases, laparoscopic cholecystectomy performed within 2 to 3 days of hospital admission is safe, feasible, and cost-effective. Compared with delayed laparoscopic cholecystectomy, early laparoscopic cholecystectomy is equally safe and feasible in the incidence of intraoperative and postoperative complications (6 RCTs, high certainty) and is associated with a reduction in the length of hospital stay (6 RCTs, high certainty). Furthermore, compared with delayed cholecystectomy, early cholecystectomy is not associated with a higher conversion rate to open cholecystectomy (5 RCTs, high certainty). No difference was found between the two strategies in terms of duration of surgery (4 RCTs, moderate certainty). Early cholecystectomy is also associated with lower recurrence rates of acute biliary pancreatitis (6 RCTs, high certainty), lower hospital management costs (1 RCT, moderate certainty), and better patient-reported quality of life and pain (1 RCT, high certainty).

Response: In patients with mild acute biliary pancreatitis, early laparoscopic cholecystectomy during index admission (or within 14 days) should be performed (MANCTRA GRADE assessment: QoE, high; SoR, strong). The available evidence supports the statement that early laparoscopic cholecystectomy should be performed in patients with mild acute biliary pancreatitis during the index admission or within 14 days (ChatGPT GRADE assessment: QoE, moderate; SoR, strong).

### Comparison of the Two GRADE Assessments (Human vs ChatGPT)

For the bundle element “In patients with acute necrotizing pancreatitis, since the timing of the operation is a risk factor for mortality and major complications, if the patient’s clinical conditions allow, any interventional strategy (preferably endoscopic or minimally invasive step-up approach) should be delayed beyond 4 weeks,” the panel assessment concluded for a strong recommendation in favor of the delayed treatment, supported by the high QoE (MANCTRA GRADE assessment SoR, strong; QoE, high). Conversely, the ChatGPT assessment reported a weak SoR in favor of the delayed treatment, supported by a low to moderate QoE (ChatGPT GRADE: SoR, weak; QoE, Low to moderate). Due to the inconsistency between the two sources of assessment, this element was not included in the bundle. Further minor discrepancies were reported between the two assessments regarding the quality of the evidence (Supplemental Digital Content Table 2, http://links.lww.com/SLA/E734).

### The 2023 MANCTRA Acute Biliary Pancreatitis Care Bundle and Future Objectives

The final 7 elements of the bundle are reported in Supplemental File (Supplemental Digital Content Table 3, http://links.lww.com/SLA/E734). The elements were included in the bundle if they were consistent with a strong recommendation and high to moderate evidence from the current literature and corroborated by the AI of ChatGPT.

- Antibiotic prophylaxis is strongly discouraged in patients with acute biliary pancreatitis of any degree unless there is a strong suspicion of an active pancreatic or extrapancreatic infection (MANCTRA GRADE: SoR, strong; QoE, high) (ChatGPT GRADE assessment: SoR, strong; QoE, high).
- Patients with mild acute biliary pancreatitis who are able to tolerate it should be fed a full-solid diet instead of nil per os (MANCTRA GRADE assessment: SoR Strong, QoE High) (ChatGPT GRADE assessment: SoR, strong; QoE, moderate to high).
- EN should be started in patients with severe acute biliary pancreatitis who are unable to be fed by mouth. TPN should only be reserved for those patients in whom it is impossible to start EN (MANCTRA GRADE assessment: SoR, strong; QoE, high) (ChatGPT GRADE assessment: SoR, strong; QoE, High).
- e-EN should be established within 48 hours of admission through nasojejunal or nasogastric routes for patients with severe acute biliary pancreatitis and inability to feed orally (MANCTRA GRADE assessment: SoR, strong; QoE, high) (ChatGPT GRADE assessment: SoR, strong; QoE, high).
- ERCP should be performed within the first 48 to 72 hours of hospital admission in patients with acute biliary pancreatitis and concomitant cholangitis (MANCTRA GRADE assessment: SoR, strong; QoE, high) (ChatGPT GRADE assessment: SoR, strong; QoE, moderate to high).
- In clinically deteriorating patients with acute necrotizing pancreatitis, associated or not with necrosis infection, the first interventional therapeutic approach should be the endoscopic step-up approach. The minimally invasive surgical step-up approach can be considered the alternative choice (MANCTRA GRADE assessment: SoR, strong; QoE, high) (ChatGPT GRADE assessment: SoR, strong; QoE, moderate to high).
- In patients with mild acute biliary pancreatitis, early laparoscopic cholecystectomy during index admission (or within 14 days) should be performed (MANCTRA GRADE assessment: SoR, strong; QoE, high) (ChatGPT GRADE assessment: SoR, strong; QoE, moderate).

### Forecasts

The panel of experts elaborated on the predictions of the potential improvements in the compliance rate for every bundle element (see Methods). These forecasts were then restated with the help of the ChatGPT AI software and compared with the improvement rates predicted by the experts. The results are shown in Supplemental File (Supplemental Digital Content Table 3, http://links.lww.com/SLA/E734).

## DISCUSSION

The 2023 MANCTRA Acute Biliary Pancreatitis Care Bundle is a set of evidence-based clinical indicators developed by a panel of internationally renowned researchers in the field of acute biliary pancreatitis, with the help of AI algorithms produced by ChatGPT. The bundle aims to enforce the cumulative effect of evidence-based pathways on daily clinical practice and is consistent with the Institute for Healthcare Improvement guidelines for developing care bundles.^12^ Critical parts of the bundle include the optimal administration of prophylactic antibiotics and nutritional support, the best timing of ERCP, and cholecystectomy. The question arises of how best to spread guideline recommendations.^8–10,40–42^ Masamune et al^43^ reported that after the introduction of the Japanese pancreatitis bundle, the mortality rate of patients with severe acute pancreatitis decreased to 6.1% in 2016, compared with the 10.1% reported in the previous nationwide survey.^44^ Following these results, the most recent Japanese guidelines implemented clinical indicators, consisting of a bundle of 10 statements that mainly specify the management and treatment measures taken within the first 48 hours.^7^ When examining the association between the mortality rate and implementation of pancreatitis bundle elements in patients with severe disease, the same group found that mortality rates decreased when 8 or more elements of the bundle were implemented (1.0% vs 7.1%).

The MANCTRA team’s effort in the revision of the contemporary evidence led to the production of the first Western acute biliary pancreatitis bundle. For the first time, the synthesis of evidence and the GRADE assessment, performed by a panel of internationally renowned researchers, was double-checked with the AI algorithms produced by ChatGPT. ChatGPT has been trained on a massive corpus of scientific publications, which has helped it to develop a deep understanding of the nuances of scientific language and the conventions of academic discourse. This enables it to provide more accurate and valuable feedback on scientific writing than other AI systems that may lack this specialized training. Furthermore, ChatGPT is constantly learning and updating its knowledge base, as it is trained on a vast and continuously expanding data set of scientific publications. This means it can stay up-to-date with the latest research and findings in any field, ensuring that its feedback and revisions are current and relevant.

It is widely recognized that there are 4 essential components of care delivery: staff, equipment, infrastructure, and systems that bring together these elements. While changes that concentrate solely on clinical protocols or management may be more easily implemented, modifications requiring equipment or infrastructure adjustments pose more significant challenges. So, implementation of bundle elements seems to be more difficult the more interventional the key recommendation, such as minimally invasive surgical operations, endoscopy, availability of specialist units and intensive care, and when the recommendation depends on factors not readily controlled by the admitting specialists.^45^

It seems that AI is well aware of the implementation challenges associated with any of the 4 components of care delivery. In keeping with this, the panel predictions of improved compliance with bundle elements differed from those generated by the ChatGPT model, especially for items focused on interventional measures, such as endoscopic and surgical step-up approach strategies for treating necrotizing pancreatitis, ERCP in cases associated with cholangitis, and early cholecystectomy. The most striking example of the discrepancy between the evaluation model carried out by the experts and the one developed by ChatGPT concerns the indication to delay any interventional procedure on infected pancreatic necrosis up to four weeks after the onset of symptoms. According to many RCTs and subsequent meta-analyses,^46,47^ interventions in the case of pancreatic necrosis should be delayed until at least 4 weeks after the onset of symptoms, provided that the patient’s clinical condition allows. The most substantial evidence in favor of delayed treatment, however, is based on studies comparing early versus delayed open surgical necrosectomy. In the era of endoscopic and minimally invasive surgery, this indication may need to be reevaluated. On this element, the assessment carried out by the AI may have identified the need to question the current indications more than the review of the evidence carried out by the expert panel could have done.

Establishing compliance objectives for each of the items is essential to achieve a satisfactory degree of compliance. The MANCTRA Acute Biliary Pancreatitis Bundle 2023 has identified goals, focusing on setting high standards for those items that can be achieved easily with knowledge of guidelines and minimal organizational effort. A plan for application, enforcement, and audit has been released for every element proposed in our bundle, with specific endpoints. With such measures, noncompliance with clinically relevant and evidence-based key recommendations can be minimized, and the lack of implementation may result in a less limiting factor in the future.

Putting recommendations into practice can take time, depending on how much change in clinical practice or services is needed. Based on current trends and assuming efforts are made to improve the transfer of knowledge, the AI model proved to be more cautious than the forecasts made by the experts, recognizing a positive variation of around 10%. Moreover, managing severe acute biliary pancreatitis requires the availability of numerous specialty services, such as gastroenterology, surgery, critical care, and interventional radiology, and the experience of coordinating a multidisciplinary team. An analysis of the United States Nationwide Inpatient sample showed that a high annual hospital volume of acute pancreatitis cases results in a shorter length of hospital stay, lower adjusted mortality, and a lower likelihood of pancreatic procedures.^48,49^ In keeping with this finding, according to the UK Party on Acute Pancreatitis, every hospital that receives acute admission should have a nominated multidisciplinary clinical team to manage patients with severe acute biliary pancreatitis. If the full range of specialists is not available in the receiving hospital, the nominated team should coordinate local management where possible and the referral to a specialist unit where appropriate.^50^ Similarly, the International Association of Pancreatology and the American Pancreatic Association stated that management in, or referral to, a specialist center is necessary for patients with severe acute biliary pancreatitis and for those who may need an interventional radiologic, endoscopic, or surgical operation.^6^

## CONCLUSIONS

We have developed a new care bundle with 7 evidence-based key elements for managing patients with acute biliary pancreatitis, whose ease of understanding predisposes to almost immediate use in clinical practice. This new bundle, whose scientific strength has been enhanced with the alliance between human knowledge and AI from the new ChatGPT software, should be introduced to emergency departments, wards, and ICUs. Future clinical trials are needed to measure the impact of adopting this Bundle for the optimal treatment of acute biliary pancreatitis.

## Supplementary Material

SUPPLEMENTARY MATERIAL
