# Supplementary material for: The 2023 MANCTRA Acute Biliary Pancreatitis Care Bundle: A Joint Effort Between Human Knowledge and Artificial Intelligence (ChatGPT) to Optimize the Care of Patients With Acute Biliary Pancreatitis in Western Countries
Source: Ann Surg. 2023 Jul 17;279(2):203–12. doi: 10.1097/SLA.0000000000006008 (PMC10782931; doi:10.1097/SLA.0000000000006008)

**SUPPLEMENTAL FILES**

**Table Legends**

**Table 1.** Summary of the MANCTRA-1 (coMpliAnce with evideNce-based cliniCal guidelines in the managemenT of acute biliaRy pancreAtitis) Audit.

**Figure Legends**

**Figure 1.** The 2023 MANCTRA Acute Biliary Pancreatitis Care Bundle.

**Figure 2.** The MANCTRA Project Goals for 2025.

**List of Supplemental Files (with Legends)**

**1. Supplemental Files Appendix 1.** Search strategies implemented for the PICO research questions.

**2. Supplemental Files Table 1.** Definitions adopted for “mild acute pancreatitis”, “severe acute pancreatitis”, “predicted severe acute pancreatitis”, “acute cholangitis”, “pancreatic necrosis”, “infected pancreatic necrosis”.

**3. Supplemental Files Table 2.** The 2023 MANCTRA Acute Biliary Pancreatitis Care Bundle development.

**4. Supplemental Files Table 3.** The 2023 MANCTRA Acute Biliary Pancreatitis Care Bundle and future objectives in compliance level.

**5. Supplemental Files Table 4.** Supplementary Table Report Template Question 1.

**6. Supplemental Files Table 5.** Supplementary Table Report Template Question 2.

**7. Supplemental Files Table 6.** Supplementary Table Report Template Question 3.

**8. Supplemental Files Table 7.** Supplementary Table Report Template Question 4.

**9. Supplemental Files Table 8.** Supplementary Table Report Template Questions 5-6.

**10. Supplemental Files Table 9.** Supplementary Table Report Template Question 7.

**11. Supplemental Files Table 10.** Supplementary Table Report Template Question 8.

**12. Supplemental Files Table 11.** Supplementary Table Report Template Question 9.

**13. Supplemental Files Table 12.** Supplementary Table Report Template Question 10.

**14. Supplemental Files Figure 1.** GRADE Assessment PICO 1. Should routine enhanced CT scan at the time of hospital admission vs. contrast-enhanced CT performed 72-96 hours after onset of symptoms be used for the diagnosis of local complications (fluid collections, pancreatic necrosis) in patients with severe acute biliary pancreatitis?

**15. Supplemental Files Figure 2.** GRADE Assessment PICO 2. Should routine prophylactic antibiotics vs. no routine prophylactic antibiotics be used for patients with acute biliary pancreatitis in the absence of infectious complications?

**16. Supplemental Files Figure 3.** GRADE Assessment PICO 3. Should serum measurements of procalcitonin (PCT) vs. other sepsis markers be used for the early diagnosis of infected pancreatic necrosis in patients with severe acute biliary pancreatitis?

**17. Supplemental Files Figure 4.** GRADE Assessment PICO 4. Should early (within 24 hours) oral feeding as tolerated vs. keeping the patient nil per os be used for patients with mild acute biliary pancreatitis (if tolerated)?

**18. Supplemental Files Figure 5.** GRADE Assessment PICO 5. Should enteral nutrition (EN) vs. total parenteral nutrition (TPN) be used for the prevention of gut failure and infectious complications in patients with acute biliary pancreatitis and the inability to feed orally?

**19. Supplemental Files Figure 6.** GRADE Assessment PICO 6. Should early enteral nutrition (eEN) within 48 hours vs. delayed enteral nutrition (dEN) beyond 48 hours be used in patients with severe acute biliary pancreatitis and inability to feed orally?

**20. Supplemental Files Figure 7.** GRADE Assessment PICO 7. Should early (within 48-72 hours) ERCP/ES vs. delayed (> 72 hours) or conservative treatment be used in gallstone-induced acute biliary pancreatitis when cholangitis and/or common bile duct obstruction occur?

**21. Supplemental Files Figure 8.** GRADE Assessment PICO 8. Should surgical or endoscopic step-up approach vs. upfront necrosectomy be used as the first line of treatment for patients with pancreatic necrosis?

**22. Supplemental Files Figure 9.** GRADE Assessment PICO 9. Should delayed (after 4 weeks) therapeutic interventions (endoscopic or surgical step-up approach, surgical necrosectomy) vs. early interventions be used for patients with acute biliary necrotizing pancreatitis who remain clinically stable?

**23. Supplemental Files Figure 10.** GRADE Assessment PICO 10. Should early laparoscopic cholecystectomy during index admission (or within 14 days) vs. delayed laparoscopic cholecystectomy after hospital discharge be used for patients with mild acute biliary pancreatitis?

**1. Supplemental Files Appendix 1.** Search strategies implemented for the PICO research questions.

1. ***Should routine enhanced CT scan at the time of hospital admission vs. contrast-enhanced CT performed 72-96 hours after onset of symptoms be used for the diagnosis of local complications (fluid collections, pancreatic necrosis) in patients with severe acute pancreatitis)?***

acute pancreatitis OR severe pancreatitis OR necrotizing pancreatitis OR infected pancreatic necrosis OR infected necrotizing pancreatitis AND early CT scan OR early computed tomography OR early imaging

1. ***Should routine prophylactic antibiotics vs. no routine prophylactic antibiotics be used for patients with acute pancreatitis in the absence of infectious complications?***

acute pancreatitis OR severe pancreatitis OR necrotizing pancreatitis AND antibiotic prophylaxis OR prophylactic antibiotic

1. ***Should serum measurements of procalcitonin (PCT) vs. other sepsis markers be used for the early diagnosis of infected pancreatic necrosis in patients with severe acute pancreatitis?***

acute pancreatitis OR severe pancreatitis OR necrotizing pancreatitis OR infected pancreatic necrosis OR infected necrotizing pancreatitis AND procalcitonin

1. ***Should early (within 24 hours) oral feeding as tolerated vs. keeping the patient nil per os be used for patients with mild acute pancreatitis (if tolerated)?***

acute pancreatitis OR severe pancreatitis OR necrotizing pancreatitis OR infected pancreatic necrosis OR infected necrotizing pancreatitis AND oral feeding OR refeeding OR early refeeding

1. ***Should enteral nutrition (EN) vs. total parenteral nutrition (TPN) be used for the prevention of gut failure and infectious complications in patients with acute pancreatitis and the inability to feed orally?***

acute pancreatitis OR severe pancreatitis OR necrotizing pancreatitis OR infected pancreatic necrosis OR infected necrotizing pancreatitis AND enteral nutrition OR enteral feeding AND total parenteral nutrition OR nil per os OR fast

1. ***Should early enteral nutrition (eEN) within 48 hours vs. delayed enteral nutrition (dEN) beyond 48 hours be used in patients with severe acute pancreatitis and inability to feed orally?***

acute pancreatitis OR severe pancreatitis OR necrotizing pancreatitis OR infected pancreatic necrosis OR infected necrotizing pancreatitis AND total parenteral nutrition AND enteral nutrition OR enteral feeding

1. ***Should early (within 48-72 hours) ERCP/ES vs. delayed (> 72 hours) or conservative treatment be used in gallstone-induced acute pancreatitis when cholangitis and/or common bile duct obstruction occur?***

acute pancreatitis OR severe pancreatitis OR acute cholangitis OR common bile duct obstruction OR biliary obstruction AND early ERCP OR early endoscopic retrograde cholangiopancreatography OR 72 hours ERCP OR 72 hours endoscopic retrograde cholangiopancreatography OR 24 hours ERCP OR 24 hours endoscopic retrograde cholangiopancreatography OR 48 hours ERCP OR 48 hours endoscopic retrograde cholangiopancreatography

1. ***Should surgical or endoscopic step-up approach vs. upfront necrosectomy be used as the first line of treatment for patients with pancreatic necrosis?***

infected pancreatic necrosis OR infected necrotizing pancreatitis AND percutaneous drainage OR percutaneous step-up approach OR surgical step-up approach OR endoscopic drainage OR endoscopic step-up approach AND necrosectomy OR surgery OR surgical necrosectomy

1. ***Should delayed (after 4 weeks) therapeutic interventions (endoscopic or surgical step-up approach, surgical necrosectomy) vs. early interventions be used for patients with necrotizing pancreatitis who remain clinically stable?***

infected pancreatic necrosis OR infected necrotizing pancreatitis AND early drainage OR early necrosectomy AND delayed drainage OR delayed necrosectomy

1. ***Should early laparoscopic cholecystectomy during index admission (or within 14 days) vs. delayed laparoscopic cholecystectomy after hospital discharge be used for patients with mild acute biliary pancreatitis?***

mild acute pancreatitis OR mild pancreatitis AND early cholecystectomy OR index admission cholecystectomy AND delayed cholecystectomy

**2. Supplemental Files Table 1.** Definitions adopted for “mild acute pancreatitis”, “severe acute pancreatitis”, “predicted severe acute pancreatitis”, “acute cholangitis”, “pancreatic necrosis”, “infected pancreatic necrosis”.

| **Definition** |  |
| --- | --- |
| Acute Biliary Pancreatitis^ç^ | Gallstone-related acute pancreatitis, with transient or persistent ampullary obstruction by stones. |
| Mild Acute Pancreatitis* | Mild acute pancreatitis is characterized by the absence of organ failure and the absence of local or systemic complications. |
| Moderately-Severe Acute Pancreatitis* | Moderately severe acute pancreatitis is characterized by the presence of transient organ failure (≤ 48 hours) or local or systemic complications (peripancreatic collection resulting in prolonged abdominal pain, leucocytosis and fever) in the absence of persistent organ failure (respiratory, cardiovascular, renal). |
| Severe Acute Pancreatitis* | Severe acute pancreatitis is characterized by persistent single or multiple organ failure (respiratory, cardiovascular, renal). |
| Predicted Severe Acute Pancreatitis^@^ | Predicted severe acute pancreatitis is characterized by APACHE II score ≥ 8, C-reactive Protein (CRP) ≥ 150 mg/L, Peripancreatic liquid shown on CT. |
| Acute Cholangitis^§^ | Acute cholangitis is defined according to the Tokyo Guidelines 2013/2018 Criteria: *A.* Systemic inflammation (A-1. Fever and/or shaking chills, A-2. Laboratory data: evidence of inflammatory response), *B.* Cholestasis (B-1. Jaundice, B-2. Laboratory data: abnormal liver function tests), *C.* Imaging (C-1. Biliary dilatation, C-2. Evidence of the etiology on imaging (stricture, stone, stent etc.).  *Suspected diagnosis:* one item in A + one item in either B or C; *Definite diagnosis:* one item in A, one item in B and one item in C. |
| Pancreatic Necrosis* | Necrosis involving both the pancreas and peripancreatic tissues, or necrosis of only the peripancreatic tissue, or necrosis of the pancreatic parenchyma alone. |
| Infected Pancreatic Necrosis* | Presence of necrosis infection can be presumed when there is extraluminal gas in the pancreatic and/or peripancreatic tissues on contrast-enhanced CT scan or when percutaneous, image-guided, fine-needle aspiration (FNA) is positive for bacteria and/or fungi on Gram stain and culture. |

^Ç^ van Geenen EJ, van der Peet DL, Bhagirath P, Mulder CJ, Bruno MJ. Etiology and diagnosis of acute biliary pancreatitis. Nat Rev Gastroenterol Hepatol. 2010 Sep;7(9):495-502. doi: 10.1038/nrgastro.2010.114. Epub 2010 Aug 10. PMID: 20703238.

* Banks PA, Bollen TL, Dervenis C, Gooszen HG, Johnson CD, Sarr MG, Tsiotos GG, Vege SS; Acute Pancreatitis Classification Working Group. Classification of acute pancreatitis--2012: revision of the Atlanta classification and definitions by international consensus. Gut. 2013 Jan;62(1):102-11. doi: 10.1136/gutjnl-2012-302779.

^@^ Eckerwall GE, Axelsson JB, Andersson RG. Early nasogastric feeding in predicted severe acute pancreatitis: A clinical, randomized study. Ann Surg. 2006 Dec;244(6):959-65; discussion 965-7. doi: 10.1097/01.sla.0000246866.01930.58. PMID: 17122621; PMCID: PMC1856625.

^§^ Miura F, Okamoto K, Takada T, Strasberg SM, Asbun HJ, Pitt HA, Gomi H, Solomkin JS, Schlossberg D, Han HS, Kim MH, Hwang TL, Chen MF, Huang WS, Kiriyama S, Itoi T, Garden OJ, Liau KH, Horiguchi A, Liu KH, Su CH, Gouma DJ, Belli G, Dervenis C, Jagannath P, Chan ACW, Lau WY, Endo I, Suzuki K, Yoon YS, de Santibañes E, Giménez ME, Jonas E, Singh H, Honda G, Asai K, Mori Y, Wada K, Higuchi R, Watanabe M, Rikiyama T, Sata N, Kano N, Umezawa A, Mukai S, Tokumura H, Hata J, Kozaka K, Iwashita Y, Hibi T, Yokoe M, Kimura T, Kitano S, Inomata M, Hirata K, Sumiyama Y, Inui K, Yamamoto M. Tokyo Guidelines 2018: initial management of acute biliary infection and flowchart for acute cholangitis. J Hepatobiliary Pancreat Sci. 2018 Jan;25(1):31-40. doi: 10.1002/jhbp.509. Epub 2018 Jan 8. PMID: 28941329.

**3. Supplemental Files Table 2.** The 2023 MANCTRA Acute Biliary Pancreatitis Care Bundle development.

|  | **MANCTRA Bundle Element** | **MANCTRA QoE** | **MANCTRA**  **SoR** | **Prompts to ChatGPT** | **ChatGPT Bundle Element** | **ChatGPT**  **QoE** | **ChatGPT**  **SoR** |
| --- | --- | --- | --- | --- | --- | --- | --- |
| **1** | The experts did not rate the certainty of the evidence obtained from the analysis of this research question high enough to produce a bundle element. | Low | Weak | 1.Perform a literature review with GRADE assessment of studies that investigate the optimal timing of CT scan in acute pancreatitis (early <72 hours or delayed ≥ 72 hours).  2.Rate the QoE and SoR. | Some studies suggest that early imaging with contrast-enhanced CT at the time of hospital admission may be useful for identifying local complications such as fluid collections and pancreatic necrosis, while others suggest that a delay of 72-96 hours may be more appropriate to allow time for the development of these complications. The decision of when to perform imaging and what type of imaging to use should be individualized based on the patient's clinical condition and the availability of resources. Ultimately, the choice of imaging strategy should be guided by clinical judgment. | Low | Weak |
| **2** | Antibiotic prophylaxis is strongly discouraged in patients with acute pancreatitis of any degree unless there is a strong suspicion of an active pancreatic or extra-pancreatic infection. | High | Strong | 1.Perform a literature review with GRADE assessment of studies that investigate the role of antibiotic prophylaxis in acute pancreatitis of any degree without suspicion of an active pancreatic or extra-pancreatic infection.  2.Rate the QoE and SoR. | The evidence suggests that the routine use of antibiotic prophylaxis in patients with acute pancreatitis is not recommended unless there is a strong suspicion of an active pancreatic or extra-pancreatic infection. | High | Strong |
| **3** | The experts did not rate the certainty of the evidence obtained from the analysis of this research question high enough to produce a bundle item. | Moderate | Weak | 1.Perform a literature review with GRADE assessment of studies that investigate the role of procalcitonin (PCT) in acute pancreatitis.  2.Rate the QoE and SoR. | The use of serial PCT measurements in the context of diagnostic and therapeutic pathways of patients with severe acute pancreatitis and infected pancreatic necrosis appears to be supported by the evidence. However, the optimal timing and frequency of PCT measurements, as well as the cutoff values for diagnosing infected pancreatic necrosis and predicting treatment response, are not yet well established. | Moderate to Low | Weak |
| **4** | Patients with mild acute pancreatitis, who are able to tolerate it, should be fed a full-solid diet instead of nil per os. | High | Strong | 1.Perform a literature review with GRADE assessment on the advantages of full-solid diet in patients with mild to moderately-severe acute pancreatitis patients who tolerate it compared to nil per os.  2.Rate the QoE and SoR. | The evidence supports the use of solid diets in patients with mild acute pancreatitis who can tolerate it. | Moderate to High | Strong |
| **5** | Enteral nutrition should be started in patients with severe acute pancreatitis unable to feed by mouth. Conversely, total parenteral nutrition should only be reserved for those patients in whom it is impossible to start enteral nutrition. | High | Strong | 1.Perform a literature review with GRADE assessment of enteral nutrition in patients with severe acute pancreatitis.  2.Rate the QoE and SoR. | The available evidence consistently supports the use of EN as the preferred method of nutritional support in patients with severe acute pancreatitis, and the potential risks associated with TPN support its limited use. | High | Strong |
| **6** | Early enteral nutrition should be established within 48 hours of admission via nasojejunal or nasogastric routes for patients with severe acute pancreatitis and inability to feed orally. | High | Strong | 1.Perform a literature review with GRADE assessment of early (within 48 hours) enteral nutrition in patients with severe acute pancreatitis.  2.Rate the QoE and SoR. | The available evidence consistently supports using EEN via nasojejunal or nasogastric routes within 48 hours of admission in patients with severe acute pancreatitis who cannot feed orally. | High | Strong |
| **7** | ERCP should be performed within the first 48-72 hours of hospital admission in patients with acute biliary pancreatitis and concomitant cholangitis. | High | Strong | 1.Perform a literature review and GRADE assessment of studies that investigate the advantages of early ERCP (within the first 48-72 hours) in patients with acute biliary pancreatitis and concomitant cholangitis.  2.Rate the QoE and SoR. | The available evidence consistently supports the use of early ERCP within 48-72 hours of hospital admission in patients with acute biliary pancreatitis and concomitant cholangitis. | Moderate to High | Strong |
| **8** | In clinically deteriorating patients with acute necrotizing pancreatitis, associated or not with necrosis infection, the first interventional therapeutic approach should be the endoscopic step-up approach.  The minimally invasive surgical step-up approach can be considered the alternative choice. | High | Strong | 1.Perform a literature review and GRADE assessment of studies that investigate the superiority of the step-up approach (endoscopic or surgical step-up) compared to other treatments in patients with necrotizing pancreatitis.  2.Rate the QoE and SoR. | The available evidence consistently supports the use of the endoscopic step-up approach as the first interventional therapeutic approach in clinically deteriorating patients with acute necrotizing pancreatitis, associated or not with necrosis infection | Moderate to High | Strong |
| **9** | In patients with acute necrotizing pancreatitis, since the timing of the operation is a risk factor for mortality and major complications, if the patient's clinical conditions allow, any interventional strategy (preferably endoscopic or minimally invasive step-up approach) should be delayed beyond 4 weeks. | High | Strong | 1.Perform a literature review and GRADE assessment of studies that investigate the most appropriate timing of intervention in patients with necrotizing pancreatitis.  2.Rate the QoE and SoR. | Delayed intervention beyond 4 weeks may be beneficial in patients with ANP, particularly if the patient's clinical conditions allow. However, it is important to note that the evidence is not consistent, and the optimal timing of intervention may depend on various factors, such as the severity of disease and the presence of infection. | Low to Moderate | Weak |
| **10** | In patients with mild acute biliary pancreatitis, early laparoscopic cholecystectomy during index admission (or within 14 days) should be performed. | High | Strong | 1.Perform a literature review and GRADE assessment of studies that investigate the most appropriate timing of laparoscopic cholecystectomy in mild acute pancreatitis.  2.Rate the QoE and SoR. | The available evidence supports the statement that early laparoscopic cholecystectomy should be performed in patients with mild acute biliary pancreatitis during the index admission or within 14 days. | Moderate | Strong |

**QoE=** Quality of Evidence; **SoR=** Strength of Recommendation.

**4. Supplemental Files Table 3.** The 2023 MANCTRA Pancreatitis Care Bundle and future objectives in compliance level.

* Increase in compliance level compared with the MANCTRA-1 International Audit (Podda M., *et al.* Pancreatology. 2022 Nov;22(7):902-916)

|  | **Bundle element of the 2023 MANCTRA Pancreatitis Care Bundle** | **Patient population** | **Expected compliance level (objectives)**  **in the MANCTRA-2 study 2025**  **according to the experts’ panel** | **Prompts to ChatGPT** | **Expected compliance level in the MANCTRA-2 study 2025 according to ChatGPT** |
| --- | --- | --- | --- | --- | --- |
| **1** | Antibiotic prophylaxis is strongly discouraged in patients with acute biliary pancreatitis of any degree unless there is a strong suspicion of an active pancreatic or extra-pancreatic infection | Patients with acute biliary pancreatitis of any degree without signs of active infection | 80.0% (+35.8%*) | Based on current trends, what are the possible compliance levels with the recommendation against antibiotic prophylaxis in acute biliary pancreatitis by 2025? | Based on current trends, it is possible that compliance levels with the recommendation against antibiotic prophylaxis in acute biliary pancreatitis could increase to around 60-70% by 2025. |
| **2** | Patients with mild acute biliary pancreatitis, who are able to tolerate it, should be fed a full-solid diet instead of nil per os. | Patients with mild to moderately-severe acute biliary pancreatitis who are able to tolerate a full-solid diet | 80.0% (+35.3%*) | Based on historical trends, what are the possible compliance levels with the recommendation in favor of a full-solid diet (instead of nil per os) in patients with mild acute biliary pancreatitis by 2025? | Based on historical trends, it's possible that the compliance rate with the recommendation in favor of a full-solid diet (instead of nil per os) in patients with mild acute biliary pancreatitis could increase by 15-20% over the next few years, resulting in a compliance rate of around 60-70% by 2025. |
| **3** | Total parenteral nutrition (TPN) should only be reserved for those patients with acute biliary pancreatitis in whom it is impossible to start enteral nutrition. | Patients with severe acute biliary pancreatitis unable to feed by mouth | 90.0% (+53.8%*) | Based on historical trends, what are the possible compliance levels with the recommendation against Total parenteral nutrition (TPN) for patients with severe acute biliary pancreatitis who are able to tolerate enteral nutrition by 2025? | Based on historical trends, it's possible that the compliance rate with the recommendation against Total Parenteral Nutrition (TPN) for patients with severe acute biliary pancreatitis who are able to tolerate enteral nutrition could increase by 5-10% over the next few years, resulting in a compliance rate of around 70-75% by 2025. |
| **4** | Early enteral nutrition should be established within 48 hours of admission via nasojejunal or nasogastric routes for patients with severe acute biliary pancreatitis and inability to feed orally. | Patients with severe acute biliary pancreatitis unable to feed by mouth | 75.0% (+41.8%*) | Based on historical trends, what are the possible compliance levels with the recommendation in favor of early start (within 48 hours of admission) of enteral nutrition via nasojejunal or nasogastric routes for patients with severe acute biliary pancreatitis and inability to feed orally by 2025? | Based on historical trends, it's possible that the compliance rate with the recommendation in favor of early start (within 48 hours of admission) of enteral nutrition via nasojejunal or nasogastric routes for patients with severe acute biliary pancreatitis and inability to feed orally could increase by 10-15% over the next few years, resulting in a compliance rate of around 45-50% by 2025. |
| **5** | ERCP should be performed within the first 48-72 hours of hospital admission in patients with acute biliary pancreatitis and concomitant cholangitis. | Patients with acute biliary pancreatitis and concomitant cholangitis | 80.0% (+34.0%*) | Based on current trends, what are the possible compliance levels with the recommendation in favor of ERCP performed within the first 48-72 hours of hospital admission in patients with acute biliary pancreatitis and concomitant cholangitis by 2025? | Based on historical trends, it's possible that the compliance rate with the recommendation in favor of ERCP performed within the first 48-72 hours of hospital admission in patients with acute biliary pancreatitis and concomitant cholangitis could increase by 5-10% over the next few years, resulting in a compliance rate of around 51-56% by 2025. |
| **6** | In clinically deteriorating patients with acute necrotizing pancreatitis, associated or not with necrosis infection, the first interventional therapeutic approach should be the endoscopic step-up approach.  The minimally invasive surgical step-up approach can be considered the alternative choice. | Patients with acute biliary necrotizing pancreatitis (with or without necrosis infection) and deteriorating clinical conditions | 75.0% (+41.3%*) | Based on current trends, what are the possible compliance levels with the recommendation in favor of the endoscopic step-up approach as the first interventional therapeutic approach in patients with acute necrotizing pancreatitis and deteriorating clinical conditions, associated or not with necrosis infection by 2025? | Based on historical trends, it's possible that the compliance rate with the recommendation in favor of the endoscopic step-up approach as the first interventional therapeutic approach in patients with acute necrotizing pancreatitis and deteriorating clinical conditions, associated or not with necrosis infection, could increase by 5-10% over the next few years, resulting in a compliance rate of around 39-44% by 2025. |
| **7** | In patients with mild acute biliary pancreatitis, early laparoscopic cholecystectomy during index admission (or within 14 days) should be performed. | Patients with mild acute biliary pancreatitis | 70.0% (+41.0%*) | Based on current trends, what are the possible compliance levels with the recommendation in favor of early laparoscopic cholecystectomy during index admission (or within 14 days) in patients with mild acute biliary pancreatitis by 2025? | Considering the available evidence and potential strategies for improving compliance, it's reasonable to expect that the compliance rates with the recommendation in favor of early laparoscopic cholecystectomy during index admission (or within 14 days) in patients with mild acute biliary pancreatitis could increase by 5-15% over the next few years, resulting in a compliance rate of around 34-44% by 2025. |

**5. Supplemental Files Table 4. Supplementary Table Report Template Question 1. Research question 1. *Should routine enhanced CT scan at the time of hospital admission vs. contrast-enhanced CT performed 72-96 hours after onset of symptoms be used for the diagnosis of local complications (fluid collections, pancreatic necrosis) in patients with severe acute biliary pancreatitis?***

| **Paper:** Accuracy of early CT findings for predicting disease course in patients with acute pancreatitis  Japanese Journal of Radiology 2017  Taydas O, *et al.* | | | |
| --- | --- | --- | --- |
| **Study type/ evidence level** | **Study details/limitations** | **Patients’ characteristics** | **Interventions** |
| **Study type:** Retrospective cohort study  **Evidence level:** Low | ***Countries:*** Turkey  ***Centers:*** School of Medicine, Department of Radiology, Hacettepe University, Ankara, Turkey  ***Setting:*** Acute pancreatitis  ***Funding sources*:** No funding from agencies in the public, commercial, no-for-profit sectors  ***Dropout rates:*** 0 (0%)  ***Limitations*:** retrospective study design, retrospective analysis of a prospectively collected database, no inter-observer variability analysis | Between October 2007 and December 2015, 194 patients diagnosed with acute pancreatitis were accepted onto the study. The diagnosis of acute pancreatitis was established according to the Revised Atlanta Classification. Five patients were excluded from the study since CT scans were not obtained within 72 hours of symptoms onset. As a result, 189 patients enrolled in the study consisted of 97 men (51.3%) and 92 women (48.7%) with a median age of 59 years (21-93 years) | CT examinations were performed within 72 hours of symptoms onset (median 43 hours, range 3-72 hours). CT images were reviewed retrospectively by a radiologist with 12 hours of experience in abdominal radiology. The reviewer was blinded to the physical examination findings, laboratory results, and radiology reports. |
| **Outcome Measurement/results** | Presence of any complication, either limited to the pancreas or involving peripancreatic structures; mortality; need for surgery or percutaneous intervention in the management of patients.  Culture positivity following percutaneous aspiration was the criterion for the diagnosis of infected collections | Significant associations between each CT-based scoring system (Balthazar, CT severity index – CTSI -) and the development of pancreatic and extrapancreatic complications (p< 0.0001). A cutoff value of >6 for CTSI and >9 for the modified version of the CTSI achieved a specificity of 98.7% and 99.2% for predicting pancreatic and extrapancreatic complications with areas under the curve (AUC) of 0.96 and 0.96, respectively. Balthazar grade of >C yielded a sensitivity of 98.4% for predicting pancreatic and extrapancreatic complications with an AUC of 0.95. The modified version of CTSI had the most significant association with pancreatic and extrapancreatic complications (HR: 3.22; p= 0.002 and HR: 2.99, p= 0.003, respectively). Pancreatic necrosis was the only parameter significantly associated with mortality (HR: 5.83, p= 0.0045) | |
| **Conclusions** | Early CT scan (within 72 hours) has a role in prediction of complications and the management of acute pancreatitis | | |

| **Paper:** Early nonenhanced abdominal computed tomography can predict mortality in severe acute pancreatitis  Journal of gastrointestinal surgery 2005  Spitzer AL, *et al.* | | | |
| --- | --- | --- | --- |
| **Study type/ evidence level** | **Study details/limitations** | **Patients’ characteristics** | **Interventions** |
| **Study type:** Post-hoc analysis of a randomized trial (Lexipafant study)  **Evidence level:** Low | ***Countries:*** USA  ***Centers:*** Departments of Surgery, University of California, San Francisco and East Bay, USA  ***Setting:*** Acute pancreatitis  ***Funding sources*:** Not declared  ***Dropout rates:*** Not applicable  ***Limitations*:** inclusion criteria established for a different study design; the association between mortality from acute pancreatitis and a normal CT might be confounded by different APACHE II scores; non-contrast-enhanced CT scan | Data from patients with acute pancreatitis enrolled in the international phase III study of the platelet-activating factor-inhibitor Lexipafant was used to analyze non-contrast CT versus acute pancreatitis mortality | Non-enhanced CT examinations of the abdomen from the trial were classiﬁed by disease severity (Balthazar grades A–E) and then correlated with patient survival; 477 patients underwent CT within 48 hours of admission and 220 patients did so over the subsequent 6 days. Six hundred ninety-seven subjects (63% men; age, 59±16 years) underwent abdominal CT examination within 8 days of hospital admission. Of these, 477 had their examination within the ﬁrst 2 days of hospital admission and 220 had their examination within 3–8 days following admission |
| **Outcome Measurement/results** | Mortality | Higher CT grades were associated with increased mortality. Each unit increase in Balthazar grade during the initial 48 hours was associated with an estimated increase in the risk of mortality of 33%, and this trend increased to 50% if pancreatic enlargement and peripancreatic stranding (grades B and C) were combined (P< 0.05). CT grade correlated minimally with Ranson, Glasgow, or APACHE II score during the initial 48 hours; however, this correlation improved over 3–8 days. | |
| **Conclusions** | Early non-enhanced abdominal CT in patients with acute pancreatitis is a valuable prognostic indicator of mortality in acute pancreatitis, even among patients without clinical features of severe acute pancreatitis. | | |

| **Paper:** The value of performing early non-enhanced CT in developing strategies for treating acute gallstone pancreatitis  Journal of gastrointestinal surgery 2016  Zhang J, *et al.* | | | |
| --- | --- | --- | --- |
| **Study type/ evidence level** | **Study details/limitations** | **Patients’ characteristics** | **Interventions** |
| **Study type:** Randomized controlled trial  **Evidence level:** Moderate | ***Countries:*** China  ***Centers:*** Department of general surgery, Pudong New Area Gongli Hospital, Shangai, China  ***Setting:*** Mild acute pancreatitis  ***Funding sources*:** Shangai Municipal Health Bureau No 201104369  ***Dropout rates***: 94 (48%)  ***Limitations*:** High risk of imprecision (small size cohort), single-center, decision to perform an early cholecystectomy was highly dependent on surgeon’s choice (presence or absence of peripancreatic fluid collections) | A total of 196 consecutive patients with suspected acute biliary pancreatitis admitted to the hospital between May 1, 2010 and April 30, 2014 were assessed for their eligibility to enroll in this study | Each patient underwent abdominal non-enhanced CT scan within 48 h after their onset of symptoms to assess the CT grade of their pancreatitis and reveal the presence or absence of gallbladder stones, CBD stones, and CBD dilation |
| **Outcome Measurement/results** | Laparoscopic cholecystectomy-related complications; increased severity of acute pancreatitis following laparoscopic cholecystectomy; mean lengths of hospital stay; laparoscopic cholecystectomy operation time; non-enhanced CT sensitivity, specificity, and accuracy to detect gallbladder and common bile duct stones | Non-enhanced CT was 89.2 % and 87.8 % accurate in detecting gallbladder stones and common bile duct stones, respectively. Totals of 49 and 53 patients were assigned to an early laparoscopic cholecystectomy (ELC) and late laparoscopic cholecystectomy (LLC) group, respectively. All patients in both groups were cured, no LC-related complications occurred, and no acute pancreatitis increased in severity following LC. The mean lengths of hospital stay and LC operation time were significantly shorter in the ELC group than in the LLC group (P <0.05) | |
| **Conclusions** | Non-enhanced CT can accurately detect peripancreatic fluid collection and biliary obstructions. Early abdominal non-enhanced CT is valuable when developing strategies for treating acute biliary pancreatitis. Patients with mild pancreatitis without organ failure or peripancreatic fluid collection can safely undergo early laparoscopic cholecystectomy without waiting for the complete resolution of their pancreatitis. | | |

| **Paper:** Analysis of the power of common diagnostic tools in the management of acute pancreatitis  Gastroenterology research and practice 2014  Nistal M, *et al.* | | | |
| --- | --- | --- | --- |
| **Study type/ evidence level** | **Study details/limitations** | **Patients’ characteristics** | **Interventions** |
| **Study type:** Retrospective cohort study  **Evidence level:** Very low | ***Countries:*** Germany  ***Centers:*** University medical center at Hamburg-Eppendorf, Hamburg, Germany  ***Setting:*** Acute pancreatitis  ***Funding sources*:** Not reported  ***Dropout rates:*** 0 (0%)  ***Limitations*:** Imprecision (small sample size), outdated Atlanta Classification, cohort heterogeneity | 154 acute pancreatitis patients with a threefold increase in plasma lipase. The mean age was 49 years; 33.8% were female and 66.2% were male. 35.7% of them had a biliary etiology, and 28.6% were caused by ongoing alcohol abuse. | A total of 50 out of 283 patients got a contrast-enhanced CT scan within 48 hours. In 11 cases (22%) the CT was followed by endoscopic or surgical interventions as therapeutic consequences compared with 19 out of 50 control cases (38%). In patients with a CT beyond 48 hours after admittance, in 6 out of 24 (25%) cases, therapeutic consequences occurred after the scan. 69 out of 283 patients (24,3%) had CRP >150 mg/dL within 48 hours after admission. 32 of them (46.4%) had severe acute pancreatitis following Atlanta classification |
| **Outcome Measurement/results** | CRP sensitivity and specificity compared with contrast-enhanced sensitivity and specificity in establishing the occurrence of severe pancreatitis | In 11 cases the CT was followed by endoscopic or surgical interventions as therapeutic consequences compared with 19 out of 50 control cases. 69 out of 283 patients (24,3%) had CRP >150 mg/dl within 48 hours after admission. 32 of them had severe acute pancreatitis. The CRP cutoff of 150 mg/L had a sensitivity of 80% and a specificity of 65%. The positive predictive value for severe pancreatitis in patients beyond the cutoff is 46.4%. The negative predictive value for severe pancreatitis in patients below the cutoff was 89.5%. | |
| **Conclusions** | Early CE-CT is usually not indicated. CRP helps to assess the course of acute pancreatitis; levels below 150 mg/dl between the first 48 h indicate a mild course in most cases. | | |

| **Paper:** Serial computed tomography is rarely necessary in patients with acute pancreatitis: a prospective study in 102 patients  Journal of the American College of Surgeons 2001  Munoz-Bongrand N*, et al.* | | | |
| --- | --- | --- | --- |
| **Study type/ evidence level** | **Study details/limitations** | **Patients’ characteristics** | **Interventions** |
| **Study type:** Prospective cohort study  **Evidence level:** Low | ***Countries:*** France  ***Centers*:** Departments of digestive surgery, body and vascular imaging, and anesthesiology, Lariboisière Hospital, Paris, France  ***Setting:*** Acute pancreatitis  ***Funding sources*:** Not reported  ***Dropout rates:*** 0%  ***Limitations*:** Imprecision (small study cohort) | 183 consecutive patients admitted the center for acute pancreatitis between 1990 and 1996. Among them, 81 patients were excluded from the study for the following reasons: 18 patients (22%) were referred from other institutions after initial management, 47 patients (58%) did not have serial CT as planned in the study, 2 patients (2%) died before the second CT, and 14 patients (17%) left the hospital without medical consent. The remaining 102 patients were included in this prospective study. | Contrast-enhanced CT was performed on the admission day and 7 days after admission. The extent of pancreatic inflammation was classified according to Balthazar grade, and intrapancreatic necrosis on these examinations was prospectively assessed and compared with clinical and biologic data and patient outcomes. |
| **Outcome Measurement/results** | Acute pancreatitis complications stratified according to Ranson’s and Balthazar's grades. | Complications developed in only 8% of patients with Ranson’s score 2. For the patients with Ranson’s score 2 and Balthazar grades A and B on day 1 CT, late CT seemed to be useless. Complication was suspected by clinical and biological tests before day 7 in 22 of 24 complicated patients (92%), suggesting that CT could be proposed only in cases of clinical or biological deterioration. Late CT was correlated with a complicated course in patients with Balthazar grades D and E or intrapancreatic necrosis >50%. Late CT was predictive of complications in cases of intrapancreatic necrosis enlarging since the first examination. | |
| **Conclusions** | There is little justification for systematic early CT, especially in patients with Ranson’s score 2. Late CT does not need to be performed routinely, but only in cases of clinical or biologic worsening. | | |

| **Paper:** Early detection of low enhanced pancreatic parenchyma by contrast-enhanced computed tomography predicts poor prognosis of patients with acute pancreatitis  Pancreas 2012  Hirota M, *et al.* | | | |
| --- | --- | --- | --- |
| **Study type/ evidence level** | **Study details/limitations** | **Patients’ characteristics** | **Interventions** |
| **Study type:** National survey  **Evidence level:** Low | ***Countries:*** Japan  ***Centers:*** National survey  ***Setting:*** Acute pancreatitis  ***Funding sources*:** Research committee of the intractable pancreatic diseases, the Ministry of Health, Labour and Welfare of Japan  ***Dropout rates:*** Not applicable  ***Limitations*:** Heterogeneity in the criteria for defining low-enhanced pancreatic parenchyma (244 different institutes) | The target subjects were patients who received a diagnosis of acute pancreatitis and treated for AP in 2007 in Japan. 983 patients (male n = 686, female n = 297) with AP were analyzed retrospectively | On admission, contrast-enhanced CT scan (CECT) was examined in 1018 patients of 2256 patients with acute pancreatitis. Because the CECT findings were not complete in the clinical records of 35 of the 1018 patients, they were excluded from this study |
| **Outcome Measurement/results** | Incidence of organ failure, infectious complications, and mortality. Infection was defined as acute pancreatitis accompanied by sepsis, infections, pancreatic necrosis, pancreatic abscess, or abdominal abscess during the clinical course | Low enhanced pancreatic parenchyma was associated with the incidence of organ failure and infectious complications as well as mortality (P <0.0001). When patients were further divided into 4 groups according to the CECT findings, the low-enhanced pancreatic parenchyma/peri-pancreatic collection (+/+) group was characterized as high morbidity and high mortality. The incidence of organ failure (28.2%), multiple organ failure (15.5%), and mortality (11.4%) in patients assigned to the (+/+) group was significantly higher than in those assigned to the other groups. The incidence of infectious complications was significantly higher in patients assigned to the (+/+) group (16.7%), the (+/-) group (9.0%), and the (-/+) group (7.0%) than those assigned to the (-/-) group (1.8%). | |
| **Conclusions** | The detection of low-enhanced pancreatic parenchyma and peri-pancreatic collections is a useful CECT finding for the early assessment of the severity of acute pancreatitis | | |

| **Paper:** Acute pancreatitis: a comparison of intervention rates precipitated by early vs guideline CT scan  Clinical Radiology 2016  Dobbs NW, *et al.* | | | |
| --- | --- | --- | --- |
| **Study type/ evidence level** | **Study details/limitations** | **Patients’ characteristics** | **Interventions** |
| **Study type:** Retrospective cohort study  **Evidence level:** Low | ***Countries:*** Scotland (UK)  ***Centers*:** Ninewells Hospital, Dundee, UK  ***Setting:*** Acute pancreatitis  ***Funding sources*:** Not reported  ***Dropout rates:*** 0%  ***Limitations*:** Single-center retrospective cohort study (sampling bias, misclassification, lack of info on the time onset of acute pancreatitis); small sample size (imprecision); intervention rate as the only outcome measure | 100 patients in the “too early” (<6 days) CT scan and 103 patients “within guideline” scans (≥6 days) | CT scan <6 days versus CT scan ≥6 days for patients with acute pancreatitis |
| **Outcome Measurement/results** | Intervention rates | No intervention was precipitated by performing CT before the sixth day of admission in AP. A statistically signiﬁcant larger number of interventions were precipitated when CT was performed on the sixth day or later (p<0.05). | |
| **Conclusions** | Performing CT before the sixth day of admission does not lead to earlier intervention. | | |

| **Paper:** Effects of early contrast-enhanced computed tomography on clinical course and complications in patients with acute pancreatitis  Z Gastroenterol 2016  Demir M, *et al.* | | | |
| --- | --- | --- | --- |
| **Study type/ evidence level** | **Study details/limitations** | **Patients’ characteristics** | **Interventions** |
| **Study type:** Retrospective cohort study  **Evidence level:** Very low | ***Countries:*** Germany  ***Centers:*** Clinic for Gastroenterology and Hepatology, University Hospital of Cologne, Germany  ***Setting:*** Acute pancreatitis  ***Funding sources*:** Not reported  ***Dropout rates:*** 0%  ***Limitations*:** Small single-center retrospective cohort study (selection bias, imprecision) | Data of 111 consecutive adult patients (≥ 18 years old) with at least 1 CECT during their hospital stay between May 2006 and January 2011 were extracted from the electronic Radiology Information System (RIS) registry of the University Hospital of Cologne with “pancreatitis” given as indication for the clinical request of a CECT. | Patients were divided into two groups: an early group (CECT within the first 48 h after the onset of symptoms, n = 32) and a late group (CECT > 48 h after the onset of symptoms, n = 26) |
| **Outcome Measurement/results** | Development of any of the following severe complications alone or in combination: death, respiratory failure requiring ventilation, acute renal failure requiring dialysis, and the need for invasive interventions (surgery or endoscopic or CT-guided puncture/drainage) | No statistically significant differences between both groups concerning baseline characteristics, CT scan severity index (CTSI), and modified CT scan severity index (MCTSI). Complications occurred more often in the early CECT group (p = 0.008). Multivariate logistic regression analysis identified an early CECT and a severe MCTSI as independent risk factors for the occurrence of severe complications (p= 0.02 and p= 0.002, respectively). | |
| **Conclusions** | CECT performed within the first 48 h after the onset of symptoms is associated with an unfavorable outcome in acute pancreatitis | | |

| **Paper:** Prognostic value of CT in the early assessment of patients with acute pancreatitis  American Journal of Radiology 2004  Casas JD, *et al.* | | | |
| --- | --- | --- | --- |
| **Study type/ evidence level** | **Study details/limitations** | **Patients’ characteristics** | **Interventions** |
| **Study type:** Retrospective cohort study  **Evidence level:** Low | ***Countries:*** Spain  ***Centers:*** Department of Radiology, Autonomous University of Barcelona, Spain  ***Setting:*** first episode of acute pancreatitis  ***Funding sources*:** Not reported  ***Dropout rates:*** 0%  ***Limitations*:** Retrospective cohort study (selection bias, imprecision) | 375 patients diagnosed with acute pancreatitis during a 4-year period (January 1999–December 2002). Two hundred twenty-seven patients were excluded from the study population on the basis of the exclusion criteria. A total of 148 patients were included in the study group, 82 men and 66 women, who were 22–93 years old (mean, 57 years) | Unenhanced and contrast-enhanced helical CT within the ﬁrst 24 hours after hospitalization and less than 72 hours after the onset of symptoms and the CT study |
| **Outcome Measurement/results** | Sensitivity and specificity of early CT scan. Morbidity and mortality prediction | Early CT grade had a sensitivity and speciﬁcity of 100% and 61.6%, respectively, for predicting morbidity and 100% and 56.9% for predicting mortality. Necrosis detection on early CT had a sensitivity and speciﬁcity of 53.3% and 90.2%, respectively, for predicting morbidity and 75% and 83.8% for mortality | |
| **Conclusions** | Early unenhanced CT is a good indicator of severity of acute pancreatitis. CT grade is sensitive for predicting outcome in acute pancreatitis. Pancreatic necrosis, estimated on early, contrast-enhanced CT and seen only in patients having severe disease, is a speciﬁc predictor of morbidity and mortality | | |

| **Paper:** Potential harmful effect of iodinated intravenous contrast medium on the clinical course of mild acute pancreatitis  Archives of surgery 2000  Carmona-Sanchez R, *et al.* | | | |
| --- | --- | --- | --- |
| **Study type/ evidence level** | **Study details/limitations** | **Patients’ characteristics** | **Interventions** |
| **Study type:** Retrospective cohort study  **Evidence level:** Very low | ***Countries:*** Mexico  ***Centers*:** Departments of gastroenterology and radiology, Instituto Nacional de la Nutricion Salvador Zubiran, Tlalpan, Mexico  ***Setting:*** Mild pancreatitis  ***Funding sources*:** Not reported  ***Dropout rates:*** 0%  ***Limitations*:** Retrospective analysis (selection bias), small cohort (imprecision), outdated classification of severity | 126 patients with mild acute pancreatitis | 52 patients underwent contrast-enhanced CT to establish acute pancreatitis diagnosis, and the remaining 74 did not |
| **Outcome Measurement/results** | Survival and development of local or systemic complications during the hospital stay | Local and systemic complications were more frequently observed in patients who underwent CT (OR, 11.4; 95% CI, 2.064.8; P= 0.008). Six patients, all in the CT group, developed a pancreatic abscess (OR, 20.8; P= 0.004). In 5 of them, a second CT showed more severe pancreatitis changes. The association between CT and abscess development was more apparent in patients with a body mass index of 25 or more and/or nasogastric suction. Six patients in the CT group and 1 in the no-CT group had systemic complications (OR, 9.5; P= 0.01). There were no deaths. | |
| **Conclusions** | The observed increased incidence of local and systemic complications in patients with mild pancreatitis who undergo contrast-enhanced CT, particularly in those with a body mass index of 25 or more, suggests a potentially harmful effect of intravenous contrast medium. It seems reasonable to restrict the use of contrast-enhanced CT to patients with severe pancreatitis, protracted clinical course, or suspected local septic complication. | | |

| **Paper:** A comparative evaluation of radiologic and clinical scoring system in the early prediction of severity in acute pancreatitis  The American Journal of Gastroenterology 2012  Bollen TL, *et al.* | | | |
| --- | --- | --- | --- |
| **Study type/ evidence level** | **Study details/limitations** | **Patients’ characteristics** | **Interventions** |
| **Study type:** Retrospective analysis of a prospectively collected database  **Evidence level:** Low | ***Countries:*** USA  ***Centers:*** Division of abdominal imaging & intervention, department of radiology, Brigham and Women’s Hospital, Harvard medical school, Boston, Massachusetts, USA  ***Setting:*** Acute pancreatitis  ***Funding sources*:** National Pancreas Foundation  ***Dropout rates:*** 0%  ***Limitations*:** Not all patients underwent a CT on the day of admission (CT performed based on the discretion of the treating physician for severity assessment), relatively small number of severe cases | A retrospective analysis of the abdominal CT data of 346 patients with acute pancreatitis was performed | Seven CT scoring systems (CT severity index (CTSI), modiﬁed CT severity index (MCTSI), pancreatic size index (PSI), extrapancreatic score (EP), ‘‘extrapancreatic inﬂammation on CT’’ score (EPIC), ‘‘mesenteric oedema and peritoneal ﬂuid’’ score (MOP), and Balthazar grade), as well as two clinical scoring systems: Acute Physiology, Age, and Chronic Health Evaluation (APACHE)-II and Bedside Index for Severity in AP (BISAP), were comparatively evaluated with regard to their ability to predict the severity of AP on admission (ﬁrst 24 h of hospitalization) |
| **Outcome Measurement/results** | Acute pancreatitis severity and mortality | Of 346 consecutive episodes of acute pancreatitis, there were 159 (46%) episodes in 150 patients (84 men, 66 women; mean age, 54 years; age range, 21–91 years) who were evaluated with a contrast-enhanced CT scan (n = 131 episodes) or an unenhanced CT scan (n = 28 episodes) on the ﬁrst day of admission. Clinically severe pancreatitis was diagnosed in 29/159 (18%) episodes; 9 (6%) patients died. Overall, the Balthazar grading system (any CT technique) and CTSI (contrast-enhanced CT only) demonstrated the highest accuracy among the CT scoring systems for predicting severity, but this was not statistically signiﬁcant | |
| **Conclusions** | A CT on admission solely for severity assessment in acute pancreatitis is not recommended | | |

| **Paper:** Influence of contrast-enhanced computed tomography on course and outcomes in patients with acute pancreatitis  Pancreas 2002  Uhl W, *et al.* | | | |
| --- | --- | --- | --- |
| **Study type/ evidence level** | **Study details/limitations** | **Patients’ characteristics** | **Interventions** |
| **Study type:** Retrospective cohort study  **Evidence level:** Low | ***Countries:*** Germany  ***Centers:*** Department of general surgery, University of Heidelberg, Germany  ***Setting:*** Moderate to severe acute pancreatitis  ***Funding sources*:** Not reported  ***Dropout rates:*** 0%  ***Limitations*:** Retrospective design (selection bias), recall bias, study populations were unbalanced because of the initial intent of the study to evaluate the effects of octreotide in a randomized design | 302 patients with moderate to severe acute pancreatitis | 264 underwent contrast-enhanced CT within 96 hours of the onset of symptoms and again during the course, but in 38 patients, no serial contrast-enhanced CT was performed. |
| **Outcome Measurement/results** | Hospital stay and mortality rate. Influences of contrast medium on the severity of disease were detected by monitoring complications during the course of treatment, C-reactive protein, and APACHE II score. | The 1-month mortality rate was less in patients with contrast-enhanced CT (6.4% versus 15.8%, p< 0.05). There were no significant differences considering the incidence of additional complications, and hospital stay was not significantly longer (29 ± 36 versus 19 ± 13 days). C-reactive protein and APACHE II scores had similar time courses. | |
| **Conclusions** | Contrast-enhanced computed tomography remains crucial in identifying patients with acute pancreatitis at high risk to develop necrosis of the pancreas and systemic complications | | |

| **Paper:** The prognosis of severe acute pancreatitis varies according to the segment presenting with low enhanced pancreatic parenchyma on early contrast-enhanced computed tomography  Pancreas 2017  Kitamura K, *et al.* | | | |
| --- | --- | --- | --- |
| **Study type/ evidence level** | **Study details/limitations** | **Patients’ characteristics** | **Interventions** |
| **Study type:** Retrospective cohort study  **Evidence level:** Moderate | ***Countries:*** Japan  ***Centers:*** Multicenter (44 centers in Japan)  ***Setting:*** Severe acute pancreatitis  ***Funding sources*:** Not reported  ***Dropout rates:*** 5.4%  ***Limitations*:** retrospective design, and evaluation of the distribution of low-enhanced pancreatic parenchyma may vary depending on the institution | 1097 patients diagnosed as having severe acute pancreatitis according to the Japanese Severity Score between January 2009 and December 2013. | Prognostic factor scores and/or contrast-enhanced CT grades within 48 hours after admission were evaluated |
| **Outcome Measurement/results** | Effect of low-enhanced pancreatic parenchyma in each segment on mortality | The numbers of patients with low-enhanced pancreatic parenchyma (LEPP) in the pancreatic head (Ph), body (Pb), or tail (Pt) were 272, 273, and 204 (with some overlaps), respectively. In multivariate analysis, LEPP in Ph and Pt was significantly related to mortality (OR, 1.94; 95% CI, 1.11–3.40, P< 0.05), for LEPP in Ph; OR, 2.44; 95% CI, 1.27–4.67, P< 0.05], for LEPP in Pt), but LEPP in Pb was unrelated to mortality (OR, 0.70; 95% CI, 0.35–1.37; P= 0.30) | |
| **Conclusions** | Presence of low-enhanced pancreatic parenchyma in Ph and Pt on early contrast-enhanced computed tomography was independently associated with increased mortality in SAP | | |

| **Paper:** Substraction color map of contrast-enhanced and unenhanced CT for the prediction of pancreatic necrosis in early stage of acute pancreatitis  American Journal of Radiology 2014  Tsuji Y, *et al.* | | | |
| --- | --- | --- | --- |
| **Study type/ evidence level** | **Study details/limitations** | **Patients’ characteristics** | **Interventions** |
| **Study type:** Retrospective cohort study  **Evidence level:** Very low | ***Countries:*** USA  ***Centers:*** Department of radiology, Mayo Clinic, Rochester, USA  ***Setting:*** Acute pancreatitis and pancreatic necrosis  ***Funding sources*:** Not reported  ***Dropout rates:*** 0%  ***Limitations*:** retrospective cohort study, small sample size (imprecision), variable CT protocol, readers had no prior training in the use of color-map images | Forty-eight patients with acute pancreatitis underwent unenhanced CT and contrast-enhanced CT within 72 hours from the onset of acute pancreatitis | Subtraction color-map images were created from unenhanced and contrast-enhanced CT using a 3D nonrigid registration method. Three radiologists reviewed two image sets: contrast-enhanced CT alone and subtraction color-map images in conjunction with contrast-enhanced CT. Readers evaluated each image set for the presence of pancreatic necrosis. The reference standard for pancreatic necrosis was CT or MRI 1 week or more after the onset of acute pancreatitis |
| **Outcome Measurement/results** | Sensitivity, specificity, and accuracy for predicting pancreatic necrosis | Eleven of the 48 patients developed pancreatic necrosis. The sensitivity, specificity, and accuracy for predicting pancreatic necrosis with contrast-enhanced CT were 64%, 97%, and 90%, respectively, for reader 1; 73%, 87%, and 83% for reader 2; and 73%, 87%, and 83% for reader 3. The sensitivity, specificity, and accuracy for predicting pancreatic necrosis with the subtraction color maps were 100%, 100%, and 100%, respectively, for reader 1; 100%, 95%, and 96% for reader 2; and 82%, 92%, and 90% for reader 3. Accuracy significantly improved with the addition of subtraction color maps compared with contrast-enhanced CT alone for reader 1 (p= 0.03) and reader 2 (p= 0.02) but not for reader 3 (p= 0.37) | |
| **Conclusions** | Subtraction color map is accurate in the diagnosis of pancreatic necrosis in the early stage of acute pancreatitis | | |

**6. Supplemental Files Table 5. Supplementary Table Report Template Question 2. Research question 2. *Should routine prophylactic antibiotics vs. no routine prophylactic antibiotics be used for patients with acute biliary pancreatitis in the absence of infectious complications?***

| **Paper:** Meta-analysis of prophylactic antibiotic use in acute necrotizing pancreatitis  British Journal of Surgery 2006  Mazaki T, *et al.* | | | |
| --- | --- | --- | --- |
| **Study type/ evidence level** | **Study details/limitations** | **Patients’ characteristics** | **Interventions** |
| **Study type:** Meta-analysis of RCTs  **Evidence level:** Moderate | ***Countries:*** Not applicable (meta-analysis)  ***Centers:*** Not applicable  ***Setting:*** Severe pancreatitis – acute necrotizing pancreatitis  ***Funding sources*:** Not reported  ***Dropout rates:*** Not applicable  ***Limitations*:** Low-quality studies were incorporated, small sample size in included studies, crossover rates in selected studies, variability in treatment (fluid resuscitation, nutritional support, type and duration of antibiotics) | There were 329 patients with acute necrotizing pancreatitis included in the meta-analysis | Six studies: 167 patients received prophylactic antibiotics and 162 were allocated to a control group |
| **Outcome Measurement/results** | Primary outcome measures were infected necrosis and death. Secondary outcome measures were non-pancreatic infections, surgical intervention and length of hospital stay | Prophylactic antibiotic use was not associated with a statistically signiﬁcant reduction in infected necrosis (RR 0.77, 95% CI 0.54 to 1.12, P= 0.173), mortality (RR 0.78, 95% 0.44 to 1.39, P= 0.404), non-pancreatic infections (RR 0.71, 95% CI 0.32 to 1.58, P= 0.402) and surgical intervention (RR 0.78, 95% CI 0.55 to 1.11, P= 0.167). It was, however, associated with a statistically signiﬁcant reduction in hospital stay (P= 0.040) | |
| **Conclusions** | Prophylactic antibiotics do not prevent infected necrosis or death in acute necrotizing pancreatitis | | |

| **Paper:** Role of antibiotic prophylaxis in necrotizing pancreatitis: a meta-analysis  Journal of gastrointestinal surgery 2015  Lim CLL, *et al.* | | | |
| --- | --- | --- | --- |
| **Study type/ evidence level** | **Study details/limitations** | **Patients’ characteristics** | **Interventions** |
| **Study type:** Meta-analysis of RCTs and cohort studies  **Evidence level:** Moderate | ***Countries:*** Not applicable (meta-analysis)  ***Centers:*** Not applicable  ***Setting:*** Necrotizing pancreatitis  ***Funding sources*:** No funding sources  ***Dropout rates:*** Not applicable  ***Limitations*:** Risk of imprecision, heterogeneity | Eleven studies involving 864 patients with necrotizing pancreatitis | Four hundred fifty-one patients were included in the antibiotic prophylaxis arm, with 413 patients in the control arm (different antibiotic schemes) |
| **Outcome Measurement/results** | Infected pancreatic necrosis, mortality, surgical interventions, fungal infection | No significant differences in the incidence of infected pancreatic necrosis were observed with prophylactic antibiotic use in all groups. Prophylactic antibiotic use was not associated with significant differences in all-cause mortality in Group 2 -RCTs- (RR=0.75; p= 0.24) but was associated with a reduction in Groups 1 -all studies- (RR=0.66, p=0.02) and -cohort studies- (RR=0.55, p=0.04). There was no statistical difference in the incidence of fungal infections and surgical interventions | |
| **Conclusions** | Antibiotic prophylaxis does not significantly reduce the incidence of infected pancreatic necrosis but may affect all-cause mortality in acute necrotizing pancreatitis. | | |

| **Paper:** Antibiotic prophylaxis is not protective in severe acute pancreatitis: a systematic review and meta-analysis  The American Journal of Surgery 2009  Jafri NS, *et al.* | | | |
| --- | --- | --- | --- |
| **Study type/ evidence level** | **Study details/limitations** | **Patients’ characteristics** | **Interventions** |
| **Study type:** Systematic review and meta-analysis of RCTs  **Evidence level:** Moderate | ***Countries:*** Not applicable (meta-analysis)  ***Centers*:** Not applicable  ***Setting:*** Severe acute pancreatitis  ***Funding sources*:** Not reported  ***Dropout rates:*** Not applicable  ***Limitations*:** Risk of imprecision | 502 patients from 8 studies | There were 253 patients with severe acute pancreatitis who received prophylactic antibiotics, and 249 patients were randomized to the placebo arm |
| **Outcome Measurement/results** | Mortality, infected necrosis, surgical intervention, non-pancreatic infections | There was no protective effect of antibiotic treatment with respect to mortality (RR 0.76, 95% CI, 0.49–1.16). With respect to morbidity, antibiotic prophylaxis did not protect against infected necrosis (RR 0.79, 95% CI, 0.56–1.11) or surgical intervention (RR 0.88; 95% CI, 0.65–1.20). There was, however, an apparent beneﬁt in regards to nonpancreatic infections (RR 0.60; 95% CI, 0.44–0.82), with a RR reduction of 40% (95% CI, 18%–56%), absolute risk reduction of 15% (95% CI, 6%–23%), and number needed to treat of 7 (95% CI, 4–17) | |
| **Conclusions** | Antibiotic prophylaxis in patients with severe acute pancreatitis does not reduce mortality or protect against infected necrosis, or frequency of surgical intervention | | |

| **Paper:** Assessment of prophylactic carbapenem antibiotics administration for severe acute pancreatitis: an updated systematic review and meta-analysis  Digestion 2022  Guo D, *et al.* | | | |
| --- | --- | --- | --- |
| **Study type/ evidence level** | **Study details/limitations** | **Patients’ characteristics** | **Interventions** |
| **Study type:** Systematic review and meta-analysis of RCTs and observational studies  **Evidence level:** Moderate | ***Countries:*** Not applicable (meta-analysis)  ***Centers:*** Not applicable  ***Setting:*** Severe acute pancreatitis  ***Funding sources*:** No fund support  ***Dropout rates:*** Not applicable  ***Limitations*:** Selection bias (different diagnostic and prognostic criteria), no sub-group analyses for cohort studies, methodological heterogeneity | Seven articles (5 randomized controlled trials and 2 retrospective observational studies) including 3.864 severe pancreatitis patients |  |
| **Outcome Measurement/results** | Incidence of overall infections, infected pancreatic necrosis, peripancreatic necrosis, extrapancreatic infection, pulmonary infection, bloodstream infection, urinary tract infection, pancreatic pseudocyst, fluid collection, organ failure, acute respiratory distress syndrome, surgical intervention, dialysis, use of respirator or ventilator, intensive care unit treatment | Prophylactic carbapenem antibiotics in severe acute pancreatitis were associated with a statistically significant reduction in the incidence of infections (OR: 0.27; p= 0.03) and complications (OR: 0.48; p= 0.009). Nevertheless, no statistically significant difference was demonstrated in the incidence of infected pancreatic or peripancreatic necrosis (OR: 0.74; p= 0.24), mortality (OR: 0.69; p= 0.17), extrapancreatic infection (OR: 0.64, p= 0.54), pulmonary infection (OR: 1.23; p= 0.69), blood infection (OR: 0.60; p= 0.35), urinary tract infection (OR: 0.97; p= 0.97), pancreatic pseudocyst (OR: 0.59; p= 0.28), fluid collection (OR: 0.91; p= 0.76), organ failure (OR: 0.63; p= 0.19), acute respiratory distress syndrome (OR: 0.80; p= 0.61), surgical intervention (OR: 0.97; p= 0.93), dialysis (OR: 2.34; p= 0.57), use of respirator or ventilator (OR: 1.90; p= 0.40), intensive care unit treatment (OR: 2.97; p= 0.18), and additional antibiotics (OR: 0.59; p= 0.28) between the experimental and control groups | |
| **Conclusions** | It is not recommended to administer routine prophylactic carbapenem antibiotics in severe acute pancreatitis | | |

| **Paper:** Assessment of prophylactic antibiotics administration for acute pancreatitis: a meta-analysis of randomized controlled trials  Chinese medical journal 2020  Ding N, *et al.* | | | |
| --- | --- | --- | --- |
| **Study type/ evidence level** | **Study details/limitations** | **Patients’ characteristics** | **Interventions** |
| **Study type:** Meta-analysis of RCTs  **Evidence level:** Moderate | ***Countries:*** Not applicable (meta-analysis)  ***Centers:*** Not applicable  ***Setting:*** Severe acute pancreatitis – Necrotizing pancreatitis  ***Funding sources*:** Not reported  ***Dropout rates:*** Not applicable  ***Limitations*:** Necrotizing and severe non-necrotizing pancreatitis were analyzed together without sub-group analyses, heterogeneity (different antibiotics, dosages, medical levels, duration and severity of the disease) | 11 RCTs involving 747 patients with pancreatic necrosis and/or severe acute pancreatitis | Intervention group (prophylactic use of antibiotics, n = 376), control group (n = 371) |
| **Outcome Measurement/results** | Infected pancreatic necrosis, surgical intervention, mortality, non-pancreatic infection | No signiﬁcant differences were found regarding antibiotic prophylaxis with respect to the incidence of infected pancreatic necrosis (OR, 0.74; 95% CI, 0.50–1.09; P= 0.13), surgical intervention (OR, 0.92; 95% CI, 0.62–1.38; P= 0.70), and mortality (OR, 0.71; 95% CI, 0.44–1.15; P= 0.16). Antibiotic prophylaxis was associated with a statistically signiﬁcant reduction in the incidence of non-pancreatic infection (OR, 0.59; 95% CI, 0.42–0.84; P= 0.004) | |
| **Conclusions** | Prophylactic antibiotics can reduce the incidence of non-pancreatic infection in patients with acute pancreatitis, but it does not impact the incidence of infected pancreatic necrosis | | |

| **Paper:** Prophylactic antibiotics cannot reduce infected pancreatic necrosis and mortality in acute necrotizing pancreatitis: evidence from a meta-analysis of randomized controlled trials  American journal of gastroenterology 2008  Bai Y, *et al.* | | | |
| --- | --- | --- | --- |
| **Study type/ evidence level** | **Study details/limitations** | **Patients’ characteristics** | **Interventions** |
| **Study type:** Meta-analysis of RCTs  **Evidence level:** High | ***Countries:*** Not applicable (meta-analysis)  ***Centers:*** Not applicable  ***Setting:*** Severe acute pancreatitis with CT-proven pancreatic necrosis  ***Funding sources*:** Financial support: none  ***Dropout rates:*** Not applicable  ***Limitations*:** no subgroup analyses based on etiology and age, timing of initiation of antibiotics, crossover rates | Seven trials involving 467 patients were included | A total of 467 patients were included in the 7 trials comparing antibiotics with controls on the prevention of infected pancreatic necrosis (236 versus 231) |
| **Outcome Measurement/results** | Infected necrosis, mortality | Infected pancreatic necrosis rates were not signiﬁcantly different (antibiotics 17.8%, controls 22.9%), RR 0.81 (95% CI 0.54–1.22). There was non-signiﬁcantly decreased mortality with antibiotics (9.3%) versus controls (15.2%), RR 0.70 (95% CI 0.42–1.17). Subsequent subgroup analysis conﬁrmed antibiotics were not statistically superior to controls in reducing infected necrosis and mortality | |
| **Conclusions** | Prophylactic antibiotics cannot reduce infected pancreatic necrosis and mortality in patients with acute necrotizing pancreatitis | | |

| **Paper:** Prophylactic antibiotics reduce pancreatic necrosis in acute necrotizing pancreatitis: a meta-analysis of randomized trials  Digestive surgery 2010  Yao L, *et al.* | | | |
| --- | --- | --- | --- |
| **Study type/ evidence level** | **Study details/limitations** | **Patients’ characteristics** | **Interventions** |
| **Study type:** Meta-analysis of RCTs  **Evidence level:** Low | ***Countries:*** Not applicable (meta-analysis)  ***Centers:*** Not applicable  ***Setting:*** Acute necrotizing pancreatitis with CT-confirmed necrosis  ***Funding sources*:** Not reported  ***Dropout rates:*** Not applicable  ***Limitations*:** High heterogeneity regarding the types of antibiotics (beta-lactam, quinolone, cephalosporin), different etiology | Patients with CT-proven pancreatic necrosis | 564 patients included in the nine trials comparing antibiotics with placebo or no treatment on prevention of infected pancreatic necrosis (287 versus 277) |
| **Outcome Measurement/results** | Infected pancreatic necrosis (IPN), mortality, nonpancreatic infection (NPN) and need for surgical intervention | Infected pancreatic necrosis was significantly reduced by treatment with antibiotics (RR 0.73, 95% CI 0.540.98, p= 0.04). Mortality (p= 0.1), NPN (p= 0.07), and need for surgical intervention (p= 0.17) were not significantly reduced by antibiotic treatment. Subsequent subgroup analysis confirmed that antibiotics were statistically superior to controls in reducing infected necrosis (p= 0.003) and also mortality (p= 0.02) in single-blinded randomized controlled trials | |
| **Conclusions** | Prophylactic antibiotic treatment reduces the occurrence of infected pancreatic necrosis but does not affect mortality, non-pancreatic necrosis, or surgical intervention in patients with acute necrotizing pancreatitis | | |

| **Paper:** Systematic review and meta-analysis of antibiotic prophylaxis in severe acute pancreatitis  Scandinavian journal of gastroenterology 2011  Wittau M, *et al.* | | | |
| --- | --- | --- | --- |
| **Study type/ evidence level** | **Study details/limitations** | **Patients’ characteristics** | **Interventions** |
| **Study type:** Meta-analysis of RCTs  **Evidence level:** High | ***Countries:*** Not applicable (meta-analysis)  ***Centers:*** Not applicable  ***Setting:*** Severe acute pancreatitis  ***Funding sources*:** Not reported  ***Dropout rates:*** Not applicable  ***Limitations*:** Risk of population heterogeneity, timing of antibiotic prophylaxis | 841 patients with severe acute pancreatitis (SAP). SAP was diagnosed with the following: Ranson score, Acute Physiology, and Chronic Health Evaluation II score, Imrie score, C-reactive protein levels >120 mg/L, Balthazar CT grade E, Atlanta criteria or according to the Bangkok Working Party Report 2002 | 420 patients were randomized to the prophylaxis group and 421 to the control group |
| **Outcome Measurement/results** | Mortality, incidence of infected pancreatic necrosis, incidence of non-pancreatic infections, surgical interventions | The use of antibiotic prophylaxis was not associated with a statistically signiﬁcant reduction in mortality (RR 0.74, 95% CI 0.501.07), in the incidence of infected pancreatic necrosis (RR 0.78, 95% CI 0.60–1.02), in the incidence of non-pancreatic infections (RR 0.70, 95% CI 0.46–1.06), and in surgical interventions (RR 0.93, 95% CI 0.72–1.20) | |
| **Conclusions** | There is no evidence that supports the routine use of antibiotic prophylaxis in patients with severe acute pancreatitis | | |

| **Paper:** Antibiotic therapy for prophylaxis against infection of pancreatic necrosis in acute pancreatitis  Cochrane 2010  Villatoro E, *et al.* | | | |
| --- | --- | --- | --- |
| **Study type/ evidence level** | **Study details/limitations** | **Patients’ characteristics** | **Interventions** |
| **Study type:** Systematic review and meta-analysis  **Evidence level:** Moderate | ***Countries:*** Not applicable (meta-analysis)  ***Centers:*** Not applicable  ***Setting:*** Acute pancreatitis with CT-proven pancreatic necrosis  ***Funding sources*:** None  ***Dropout rates:*** Not applicable  ***Limitations*:** None of the studies included was adequately powered | Seven evaluable studies randomised 404 patients | 203 patients received antibiotic prophylaxis and 201 patients did not. Subgroup analyses were performed for antibiotic regimen (betalactam, quinolone, and imipenem) |
| **Outcome Measurement/results** | Mortality, pancreatic infection. Secondary end-points included non-pancreatic infection, all sites infection, operative rates, fungal infections, and antibiotic resistance | There was no statistically signiﬁcant effect on the reduction of mortality with therapy: 8.4% versus controls 14.4%, and infected pancreatic necrosis rates: 19.7% versus controls 24.4%. Non-pancreatic infection rates and the incidence of overall infections were not signiﬁcantly reduced with antibiotics: 23.7% versus 36%; 37.5% versus 51.9%, respectively. Operative treatment and fungal infections were not signiﬁcantly different. With beta-lactam antibiotic prophylaxis, there was less mortality (9.4% treatment, 15% controls) and less infected pancreatic necrosis (16.8% treatment group, 24.2% controls), but this was not statistically signiﬁcant. The incidence of non-pancreatic infections was non-signiﬁcantly different (21% versus 32.5%), as was the incidence of overall infections (34.4% versus 52.8%) and operative treatment rates. No signiﬁcant differences were seen with quinolone plus imidazole in any of the endpoints measured. Imipenem on its own showed no difference in the incidence of mortality, but there was a signiﬁcant reduction in the rate of pancreatic infection (p= 0.02; RR 0.34, 95% CI 0.13 to 0.84) | |
| **Conclusions** | No beneﬁt of antibiotics in preventing infection of pancreatic necrosis or mortality was found, except for when imipenem (a betalactam) was considered on its own, where a signiﬁcantly decrease in pancreatic infection was found | | |

| **Paper:** Early prophylactic antibiotic administration for acute necrotizing pancreatitis: a meta-analysis of randomized controlled trials  Journal of hepatobiliary pancreatic science 2015  Ukai T, *et al.* | | | |
| --- | --- | --- | --- |
| **Study type/ evidence level** | **Study details/limitations** | **Patients’ characteristics** | **Interventions** |
| **Study type:** Systematic review and meta-analysis of RCTs  **Evidence level:** Moderate | ***Countries:*** Not applicable (meta-analysis)  ***Centers:*** Not applicable  ***Setting:*** Acute necrotizing pancreatitis  ***Funding sources*:** Endowment from the Department of Community Medicine, Mie University School of Medicine, Japan  ***Dropout rates:*** Not applicable  ***Limitations*:** Small sample size (high risk of imprecision), mean Jadad scale 3.0, timing of administration of antibiotics heterogenous, different regimens | 397 patients with acute necrotizing pancreatitis | All trials compared patients treated with antibiotics as the intervention group to patients treated without antibiotics as the control group. These groups comprised a total of 202 and 195 patients, respectively |
| **Outcome Measurement/results** | Mortality, incidence of infected pancreatic necrosis | The mortality rates were signiﬁcantly different for those taking antibiotics (7.4%), and controls (14.4%) (OR, 0.48; 95% CI, 0.25–0.94). Early prophylactic antibiotics use was associated with reduced incidence of infected pancreatic necrosis (antibiotics 16.3%, controls 25.1%; OR, 0.55; 95% CI, 0.33–0.92) | |
| **Conclusions** | Early use of prophylactic antibiotics for acute necrotizing pancreatitis is associated with reduced mortality and lower incidence of infected pancreatic necrosis | | |

| **Paper:** Prevention of infectious complications in acute pancreatitis  Pancreas 2019  Poropat G, *et al.* | | | |
| --- | --- | --- | --- |
| **Study type/ evidence level** | **Study details/limitations** | **Patients’ characteristics** | **Interventions** |
| **Study type:** Randomized Controlled Trial  **Evidence level:** High | ***Countries:*** Croatia  ***Centers:*** Department of Gastroenterology, Clinical Hospital Center Rijeka, Faculty of Medicine, University of Rijeka  ***Setting:*** Eligible patients were at least 18 years of age with the first episode of acute pancreatitis and a calculated Acute Physiology and Chronic Health Evaluation II (APACHE II) score of ≥ 8, regardless of etiology, who presented at the hospital within 72 hours of symptoms onset  ***Funding sources*:** Foundation of the University of Rijeka  ***Dropout rates:*** 2.9%  ***Limitations*:** Small sample size | After excluding 211 patients, 101 patients with acute pancreatitis were randomized, with 51 being allocated to the imipenem group and 50 to the placebo group. Two patients were excluded from the imipenem group because of a miscalculation of the APACHE II score, and 1 patient was excluded from the placebo group because of having chronic pancreatitis. A total of 49 patients per group were finally analyzed | A total of 49 patients per group (imipenem versus placebo) were finally analyzed |
| **Outcome Measurement/results** | Infectious complications, infected pancreatic necrosis, pneumonia, urinary tract infection, positive blood culture, sepsis | Infectious complications were present in 10 versus 12 of 49 patients (RR 0.833; 95% CI, 0.398–1.747). There were no significant differences in infected pancreatic necrosis (RR 1.5; 95% CI, 0.262–8.588), pneumonia (RR 1.5; 95% CI, 0.262–8.588), urinary tract infection (RR 0.6; 95% CI, 0.152–2.374), positive blood cultures (RR 0.5; 95% CI, 0.047–5.336), sepsis (RR 0.333; 95% CI, 0.036–3.095), and other (RR 1.333; 95% CI, 0.315–5.648) | |
| **Conclusions** | There is currently no ground to support prophylactic use of antibiotics in predicted severe acute pancreatitis | | |

**7. Supplemental Files Table 6.** **Supplementary Table Report Template Question 3.**

| **Paper:** Predictors of severe and critical pancreatitis: a systematic review  Digestive and liver disease 2014  Yang CJ, *et al.* | | | |
| --- | --- | --- | --- |
| **Study type/ evidence level** | **Study details/limitations** | **Patients’ characteristics** | **Interventions** |
| **Study type:** Systematic review of prospective clinical studies  **Evidence level:** Moderate | ***Countries:*** Not applicable (Systematic review)  ***Centers:*** Not applicable  ***Setting:*** Severe and critical acute pancreatitis  ***Funding sources*:** Not reported  ***Dropout rates:*** Not applicable  ***Limitations*:** Different populations (heterogeneity), different timings for the tests, some predictors were investigated in only few studies | Patients with severe (organ dysfunction) and critical (organ dysfunction plus infected pancreatic necrosis) acute pancreatitis. | Assessment of different severity scales in the prediction of outcomes |
| **Outcome Measurement/results** | Predictors of persistent organ failure, infected pancreatic necrosis, or both, with strict eligibility criteria. | The best predictors of persistent organ failure were the Japanese Severity Score and Bedside Index of Severity in Acute Pancreatitis when the evaluation was performed within 48 h of admission, and blood urea nitrogen and Japanese Severity Score after 48 h of admission. Systemic Inﬂammation Response Syndrome was a poor predictor of persistent organ failure. The best predictor of infected pancreatic necrosis was procalcitonin. | |
| **Conclusions** | Procalcitonin is the best predictor of infected pancreatic necrosis | | |

**Research question 3.** ***Should serum measurements of procalcitonin (PCT) vs. other sepsis markers be used for the early diagnosis of infected pancreatic necrosis in patients with severe acute biliary pancreatitis?***

| **Paper:** The evaluation of inflammatory biomarkers in predicting progression of acute pancreatitis to pancreatic necrosis: a diagnostic test accuracy review  Healthcare 2022  Riaz HMA, *et al.* | | | |
| --- | --- | --- | --- |
| **Study type/ evidence level** | **Study details/limitations** | **Patients’ characteristics** | **Interventions** |
| **Study type:** Systematic review of non-randomized cohort studies  **Evidence level:** Moderate | ***Countries:*** Not applicable (Systematic review)  ***Centers:*** Not applicable  ***Setting:*** Acute pancreatitis within two weeks of the onset of symptoms  ***Funding sources*:** No external fundings  ***Dropout rates:*** Not applicable  ***Limitations*:** Reliability of measuring or timing | The five primary clinical studies pooled 645 participants and provided data on the diagnostic accuracy of the inflammatory tests | Dosage of inﬂammatory biomarkers (C-reactive protein (CRP), procalcitonin (PCT), and lactate dehydrogenase (LDH)) |
| **Outcome Measurement/results** | Diagnostic accuracy of inﬂammatory biomarkers in detecting pancreatic necrosis in adults with conﬁrmed acute pancreatitis within 14 days of symptom onset and without organ failure | CRP had the best cutoff at 279 mg/L (χ 2 = 47.43, p < 0.001), followed by 200 mg/L (χ 2 = 36.54, p < 0.001). LDH had the cutoff at 290 units/L (χ 2 = 51.6, p < 0.001), whereas PCT did not display the most reliable results at 0.05 ng/mL | |
| **Conclusions** | PCT’s threshold was 0.5 ng/mL for all the studies | | |

| **Paper:** The diagnostic value of procalcitonin in patients with severe acute pancreatitis: a meta-analysis  Turkish journal of gastroenterology 2022  Chen L, *et al.* | | | |
| --- | --- | --- | --- |
| **Study type/ evidence level** | **Study details/limitations** | **Patients’ characteristics** | **Interventions** |
| **Study type:** Meta-analysis of observational studies  **Evidence level:** Moderate | ***Countries:*** Not applicable (Meta-analysis)  ***Centers:*** Not applicable  ***Setting:*** Severe acute pancreatitis  ***Funding sources*:** Supported by Hainan Province Clinical Medical Center  ***Dropout rates:*** Not applicable  ***Limitations*:** Limited number of included studies and high heterogeneity | Patients with severe acute pancreatitis | Sensitivity, specificity, and other measures of the accuracy of procalcitonin in the diagnosis of severe acute pancreatitis were pooled |
| **Outcome Measurement/results** | Sensitivity, specificity, and other measures of the accuracy of procalcitonin in predicting severe pancreatitis | A total of 18 studies meeting the inclusion criteria were included, containing 1764 patients. The pooled sensitivity, specificity, positive likelihood ratio, negative likelihood ratio, diagnostic odds ratio, and area under the receiver operating characteristic curve of procalcitonin for diagnosing severe acute pancreatitis were as follows: 0.80 (95% CI: 0.73-0.86), 0.84 (95% CI: 0.78-0.88), 4.95 (95% CI: 3.46-7.09), 0.23 (95% CI: 0.16-0.34), 21.26 (95% CI: 11.09-40.74), 0.89 (95% CI: 0.86-0.92) | |
| **Conclusions** | Procalcitonin has good sensitivity and diagnostic accuracy for severe acute pancreatitis | | |

| **Paper:** Serum C-reactive protein, procalcitonin, and lactate dehydrogenase for the diagnosis of pancreatic necrosis  Cochrane database of systematic reviews 2017  Komolafe O, *et al.* | | | |
| --- | --- | --- | --- |
| **Study type/ evidence level** | **Study details/limitations** | **Patients’ characteristics** | **Interventions** |
| **Study type:** Systematic review and meta-analysis  **Evidence level:** High | ***Countries:*** Not applicable  ***Centers*:** Not applicable  ***Setting:*** Patients with necrotizing pancreatitis  ***Funding sources*:** University College London, UK; National Institute for Health Research, UK.  ***Dropout rates:*** Not applicable  ***Limitations*:** Paucity of data and methodological deficiencies | Patients with acute necrotizing pancreatitis, confirmed by radiological features of pancreatic necrosis (contrast-enhanced CT or MRI), surgeon's judgment of pancreatic necrosis during surgery, or histological confirmation of pancreatic necrosis | Studies that evaluated the diagnostic test accuracy of CRP, procalcitonin, and LDH for the diagnosis of pancreatic necrosis were included |
| **Outcome Measurement/results** | Sensitivity, specificity, the post-test probability of a positive and negative index test along with 95% confidence interval (CI) on each of the different days of admission and measured at  different cut-off levels | Three studies, including 242 participants, met the inclusion criteria for this review. One study reported the diagnostic performance of CRP for two threshold levels (> 200 mg/L and > 279 mg/L) without stating the day the CRP was measured. One study reported the diagnostic performance of procalcitonin on day 1 (1 day after admission) using a threshold level of 0.5 ng/mL. One study reported the diagnostic performance of CRP on day 3 (3 days after admission) using a threshold level of 140 mg/L and LDH on day 5 (5 days after admission) using a threshold level of 290 U/L. The sensitivities and specificities varied: the point estimate of the sensitivities ranged from 0.72 to 0.88, while the point estimate of the specificities ranged from 0.75 to 1.00 for the different index tests on different days of hospital admission. However, the confidence intervals were wide: confidence intervals of sensitivities ranged from 0.51 to 0.97, while those of specificities ranged from 0.18 to 1.00 for the different tests on different days of hospital admission | |
| **Conclusions** | None of the tests assessed in this review were sufficiently accurate to suggest that they could be useful in clinical practice | | |

| **Paper:** Inflammatory mediators in the diagnosis and treatment of acute pancreatitis: pentraxin-3, procalcitonin and myeloperoxidase  Arch Med Sci 2015  Simsek O, *et al.* | | | |
| --- | --- | --- | --- |
| **Study type/ evidence level** | **Study details/limitations** | **Patients’ characteristics** | **Interventions** |
| **Study type:** Retrospective cohort study  **Evidence level:** Very low | ***Countries:*** *Turkey*  ***Centers:*** Cerrahpasa Medical Faculty, Istanbul University, Istanbul, Turkey  ***Setting:*** Acute pancreatitis  ***Funding sources*:** Not reported  ***Dropout rates:*** 0%  ***Limitations*:** Small sample size, pre-clinical study | The study group comprised 44 acute pancreatitis patients (22 male, 22 female; age: 49.3 ±16.9 years) and a control group of 30 healthy volunteers (18 male, 12 female; age: 50.8 ±12.6 years) | Dosage of leukocytes, glucose, aspartate aminotransferase (AST (SGOT)), alanine aminotransferase (ALT (SGPT)), alkaline phosphatase (ALP), total and direct bilirubin, CRP, PTX-3, MPO, and PCT levels |
| **Outcome Measurement/results** | See “Interventions” | Leukocytes, glucose, aspartate aminotransferase (AST (SGOT)), alanine aminotransferase (ALT (SGPT)), alkaline phosphatase (ALP), total and direct bilirubin levels were significantly higher in the AP group (p < 0.05, all). CRP, PTX-3, MPO and PCT were considerably higher in the AP group (p < 0.001, all), and after treatment, CRP, PTX-3, MPO and PCT levels were significantly lower (p < 0.001, all) | |
| **Conclusions** | The CRP, PTX-3, MPO, and PCT levels increase in patients with AP and hence these indicators can be used as diagnostic factors to predict inflammation severity in AP | | |

| **Paper:** Dynamics of serum procalcitonin can predict outcome in patients of infected pancreatic necrosis: a prospective analysis  Digestive diseases and sciences 2022  Samanta J, *et al.* | | | |
| --- | --- | --- | --- |
| **Study type/ evidence level** | **Study details/limitations** | **Patients’ characteristics** | **Interventions** |
| **Study type:** Prospective cohort study  **Evidence level:** Moderate | ***Countries:*** India  ***Centers:*** Post Graduate Institute of Medical Education and Research, India, Chandigarh  ***Setting:*** Acute pancreatitis with infected pancreatic necrosis  ***Funding sources*:** No funding  ***Dropout rates:*** 0%  ***Limitations*:** Small sample size (imprecision) | 242 patients of acute pancreatitis with Infected Pancreatic Necrosis (IPN) without prior intervention | PCT was measured at baseline, prior to intervention, and thereafter every 72 h. Patients were grouped into those having baseline PCT < 1.0 ng/mL and those with PCT ≥ 1.0 ng/mL |
| **Outcome Measurement/results** | Severe disease, ICU admission, mortality | Of the 242 patients screened, 103 cases (66 males; 64.1%) with IPN were grouped into 2: PCT < 1.0 ng/mL (n = 29) and PCT ≥ 1.0 ng/mL (n = 74). Patients with baseline PCT ≥ 1.0 ng/mL had significantly more severe disease scores. 16 out of 19 patients with rise in PCT on day-7 post-intervention expired. PCT ≥ 1.0 ng/mL group had higher need for ICU (p = 0.001) and mortality (p = 0.044). PCT > 2.25 ng/mL (aOR 22.56; p = 0.013) at baseline and failure in reduction of PCT levels to < 60% of baseline at day-7 post-intervention (aOR 53.76; p = 0.001) were significant mortality predictors | |
| **Conclusions** | Baseline PCT > 1.0 ng/mL is associated with poor outcome. PCT > 2.25 ng/mL and failure in reduction of PCT levels to < 60% of its baseline at day-7 post-intervention can identify high-mortality risk patients | | |

| **Paper:** Comparison of predictive systems in severe acute pancreatitis according to the Revised Atlanta Classification  Pancreas 2016  Lee KJ, *et al.* | | | |
| --- | --- | --- | --- |
| **Study type/ evidence level** | **Study details/limitations** | **Patients’ characteristics** | **Interventions** |
| **Study type:** Prospective cohort study  **Evidence level:** Moderate | ***Countries:*** *Republic of Korea*  ***Centers:*** Myongji Hospital, Yonsei University, Korea  ***Setting:*** Acute pancreatitis  ***Funding sources*:** Supported by Gachon University Gil Medical Center and Basic Science Research Program (National research foundation of Korea, Ministry of education, science and technology)  ***Dropout rates:*** 0%  ***Limitations*:** Non-randomized study design, limited number of deaths, CRP was the only parameter repeated after the admission | 146 patients with acute pancreatitis (mean age, 50.6 ± 18.3 years; 63% male), of which 43 patients (29.5%) received a diagnosis of moderately severe AP, and 17 patients (11.6%) received a diagnosis of severe acute pancreatitis | C-reactive protein (CRP) and procalcitonin obtained on admission; CRP level 24 hours after admission (CRP2). Ranson’s score, Acute Physiology and Chronic Health Examination (APACHE II), the Bedside Index for Severity in Acute Pancreatitis, and Computed Tomography Severity Index (CTSI) were calculated |
| **Outcome Measurement/results** | Prognostic value of various predictors and complex scoring systems for prediction of severe acute pancreatitis according to the revised Atlanta classification | In patients with moderately severe acute pancreatitis to severe acute pancreatitis, CTSI (OR 10.46; 95% CI, 4.3–25.43; P< 0.001), APACHE II (OR 3.87; 95% CI, 1.18–12.64; P= 0.025), and CRP2 (OR 4.5; 95% CI, 1.53–13.1; P= 0.006) were strongly related to moderately severe acute pancreatitis and severe acute pancreatitis. In patients with severe acute pancreatitis compared with mild to moderately severe acute pancreatitis, procalcitonin (OR 4.36; 95% CI, 1.01–18.96; P= 0.049) was the only factor strongly associated with severe acute pancreatitis | |
| **Conclusions** | Procalcitonin is the best predictor for patients with severe acute pancreatitis; CTSI, APACHE II, and CRP2 are valuable predictors for patients with moderately severe acute pancreatitis and SAP. | | |

| **Paper:** Comparison of Ranson, Glasgow, MOSS, SIRS, BISAP, APACHE-II, CTSI Scores, IL-6, CRP, and procalcitonin in predicting severity, organ failure, pancreatic necrosis, and mortality in acute pancreatitis  HPB Surgery 2013  Khanna AK, *et al.* | | | |
| --- | --- | --- | --- |
| **Study type/ evidence level** | **Study details/limitations** | **Patients’ characteristics** | **Interventions** |
| **Study type:** Prospective cohort study  **Evidence level:** Low | ***Countries:*** India  ***Centers***: Department of general surgery, Institute of Medical Sciences, Banaras Hindu University, Varanasi. Ultra Pradesh, India  ***Setting:*** *Acute pancreatitis*  ***Funding sources*:** No financial assistance from any source which requires to be mentioned  ***Dropout rates:*** 0%  ***Limitations*:** Small sample size (imprecision) | 72 patients with acute pancreatitis; 31 patients had organ failure and local complications classified as severe acute pancreatitis, 17 had pancreatic necrosis, and 9 died (12.5%) | BISAP, APACHE-II, MOSS, and SIRS scores were calculated using data within 24 hours of admission, whereas Ranson and Glasgow scores after 48 hours of admission; CTSI was calculated on day 4 whereas IL-6 and CRP values at end of study |
| **Outcome Measurement/results** | Predictive accuracy of scoring systems, sensitivity, specificity, and positive and negative predictive values of various markers in prediction of severe acute pancreatitis, organ failure, pancreatic necrosis, admission to intensive care units and mortality | Area under curves for Ranson, Glasgow, MOSS, SIRS, APACHE-II, BISAP, CTSI, IL-6, and CRP in predicting SAP were 0.85, 0.75, 0.73, 0.73, 0.88, 0.80, 0.90, and 0.91, respectively, for pancreatic necrosis 0.70, 0.64, 0.61, 0.61, 0.68, 0.61, 0.75, 0.86, and 0.90, respectively, and for mortality 0.84, 0.83, 0.77, 0.76, 0.86, 0.83, 0.57, 0.80, and 0.75, respectively. Procalcitonin showed 100% sensitivity, 51.4% specificity, 29.2% PPV, 100% NPV and 59.5% accuracy for mortality   \|  \| \| --- \| | |
| **Conclusions** | Procalcitonin has 100% sensitivity for prediction of organ failure and mortality, with a sensitivity of 86.4% for prediction of severe acute pancreatitis | | |

| **Paper:** Comparison of multifactor scoring systems and single serum markers for the early prediction of the severity of acute pancreatitis  Journal of gastroenterology and hepatology 2017  He WH, *et al.* | | | |
| --- | --- | --- | --- |
| **Study type/ evidence level** | **Study details/limitations** | **Patients’ characteristics** | **Interventions** |
| **Study type:** Prospective cohort study  **Evidence level:** Moderate | ***Countries:*** China  ***Centers:*** Department of gastroenterology, the first affiliated hospital of Nanchang University, Nanchang, China  ***Setting:*** Acute pancreatitis  ***Funding sources*:** Supported by the National Clinical Key Specialty Construction Project 2011 and the Graduate Special Fund for Innovative Projects in Jiangxi  ***Dropout rates:*** 0%  ***Limitations*:** - | 708 consecutive patients with acute pancreatitis | The severity was classiﬁed using the revised Atlanta and determinant-based classiﬁcation systems |
| **Outcome Measurement/results** | The predictive accuracies for moderately severe AP (MSAP), severe acute pancreatitis (SAP), critically severe acute pancreatitis (CAP), infected pancreatic necrosis (IPN), and mortality were measured using area under the receiver operating characteristic curves | The APACHE-II score had the highest accuracy in predicting severe acute pancreatitis, with area under the curve (AUC) values of 0.75 (95% CI = 0.71–0.79) and 0.77 (95% CI = 0.73–0.81) at 24 and 48 hours after admission, respectively. Procalcitonin was the most accurate predictor for CAP and IPN, with respective AUCs of 0.86 (95% CI = 0.82–0.89) and 0.83 (95% CI = 0.78–0.87) at 48 h after admission. In predicting mortality, both the APACHE II score and blood urea nitrogen had the highest accuracy | |
| **Conclusions** | The APACHE-II score had the highest predictive accuracy for SAP and mortality as deﬁned by the revised Atlanta classiﬁcation, whereas procalcitonin was the most accurate predictor for CAP and IPN | | |

| **Paper:** Procalcitonin levels predict acute kidney injury and prognosis in acute pancreatitis: a prospective study  PLOS ONE 2013  Huang HL, *et al.* | | | |
| --- | --- | --- | --- |
| **Study type/ evidence level** | **Study details/limitations** | **Patients’ characteristics** | **Interventions** |
| **Study type:** Prospective cohort study  **Evidence level:** Moderate | ***Countries:*** China  ***Centers:*** Department of laboratory medicine, West China Hospital, Sichuan University, Chengdu, China  ***Setting:*** Acute pancreatitis  ***Funding sources*:** Sichuan province-supporting technology project  ***Dropout rates:*** 0%  ***Limitations*:** Small sample size (imprecision) | 305 patients with acute pancreatitis admitted to ICU | Serum levels of PCT, serum amyloid A (SAA), interleukin-6 (IL-6), and C reactive protein (CRP) were determined on admission. Serum PCT was tested in patients who developed AKI on the day of AKI occurrence and on either day 28 after occurrence (for survivors) or on the day of death (for those who died within 28 days) |
| **Outcome Measurement/results** | Acute kidney injury (AKI) | Serum PCT levels were 100-fold higher in the AKI group than in the non-AKI group on the day of ICU admission (p< 0.05). The area under the receiver-operating characteristic (ROC) curve of PCT for predicting AKI was 0.986, which was superior to SAA, CRP, and IL-6 (p< 0.05). ROC analysis revealed all variables tested had lower predictive performance for AKI prognosis. The average serum PCT level on day 28 (2.67 (0.89, 7.99) ng/ml) was significantly (p< 0.0001) lower than on the day of AKI occurrence (43.71 (19.24,65.69) ng/ml) in survivors, but the serum PCT level on death (63.73 (34.22,94.30) ng/ml) was higher than on the day of AKI occurrence (37.55 (18.70,74.12) ng/ml) in non-survivors, although there was no significant difference between the two days in the latter group (p= 0.1365) | |
| **Conclusions** | Serum PCT is superior to CRP, IL-6, and SAA for predicting the development of AKI in patients with AP, and it can be used for dynamic evaluation of AKI prognosis | | |

| **Paper:** The predictive value of procalcitonin combined with C-reactive protein and D dimer in moderately severe and severe acute pancreatitis  European Journal of Gastroenterology & Hepatology 2022  He QY, *et al.* | | | |
| --- | --- | --- | --- |
| **Study type/ evidence level** | **Study details/limitations** | **Patients’ characteristics** | **Interventions** |
| **Study type:** Retrospective cohort study  **Evidence level:** Moderate | ***Countries:*** China  ***Centers:*** The first affiliated hospital of Fujian Medical University, Golou District, Fuzhou, Fujian, China  ***Setting:*** Moderately-severe and severe acute pancreatitis  ***Funding sources*:** Natural Science Foundation of Fujian Province  ***Dropout rates:*** 0%  ***Limitations*:** Small sample size (imprecision), retrospective, single-center study, no long dynamic observation of procalcitonin (PCT) and C-reactive protein (CRP) values | 238 patients were enrolled, of which 170 patients with moderately severe and severe acute pancreatitis (MSAP and SAP) | Concentrations of procalcitonin, CRP and D-dimer within 48 h after admission |
| **Outcome Measurement/results** | Predictive value of the parametric model, modified computed tomography severity index (MCTSI), bedside index for severity in acute pancreatitis (BISAP), Ranson score, Acute Physiology and Chronic Health Evaluation II (APACHE II) score, modified Marshall score and systemic inflammatory response syndrome (SIRS) score of all patients | The area under receiver operator characteristic curve (AUROC), sensitivity, specificity, Youden index, and critical value of the parametric model for predicting MSAP and SAP were 0.853 (95% CI, 0.804–0.903), 84.71%, 70.59%, 55.30% and 0.2833, respectively. The sensitivity of the parametric model was higher than that of MCTSI (84.00%), Ranson score (73.53%), BISAP (56.47%), APACHE II score (27.65%), modified Marshall score (17.06%) and SIRS score (78.24%); the specificity of it was higher than that of MCTSI (52.94%) and Ranson score (67.65%), but lower than BISAP (73.53%), APACHE II score (76.47%), modified Marshall score (100%) and SIRS score (100.00%) | |
| **Conclusions** | The parametric model constructed by using procalcitonin at 48 hours, CRP at 48 hours, and D dimer at 48 hours can be regarded as an evaluation model for predicting moderately severe and severe acute pancreatitis | | |

| **Paper:** A procalcitonin-based guideline promotes shorter duration of antibiotic use safely in acute pancreatitis  Journal of infection 2014  Cai Y, *et al.* | | | |
| --- | --- | --- | --- |
| **Study type/ evidence level** | **Study details/limitations** | **Patients’ characteristics** | **Interventions** |
| **Study type:** Research letter – Retrospective cohort study  **Evidence level:** Low | ***Countries:*** Singapore  ***Centers:*** Singapore General Hospital  ***Setting:*** Patients with acute pancreatitis  ***Funding sources*:** Not reported  ***Dropout rates:*** Not applicable (retrospective study)  ***Limitations*:** Retrospective design; adequately powered only for the primary outcome measure | Patients admitted from January to December 2011 with a primary diagnosis of acute pancreatitis (AP). Patients were excluded if they were severely immunosuppressed; for patients with recurrent AP, only the ﬁrst episode was included. Included patients were segregated into two groups: adherence (Group I) and non-adherence to protocol (Group II) (see interventions) | Group I (procalcitonin-guide antibiotic treatment); Group II (non-adherence to the protocol) |
| **Outcome Measurement/results** | The primary outcome was the difference in intravenous antibiotic use (days of therapy). Secondary outcomes included differences in 30-day crude mortality, days to enteral feeding, days to the resolution of fever, and white blood cell (WBC) count | A total of 225 patients were included, with less than half managed in adherence to the guideline (43.1% in Group I, 56.9% in Group II). The mean unadjusted duration of antibiotic prescription in Group I was signiﬁcantly shorter [mean difference -3.03 days (p < 0.001)].  There were no signiﬁcant differences in the incidence of crude 30-day mortality, ICU stay, and days to enteral feeding. There were no signiﬁcant differences in days to the resolution of fever or WBC count | |
| **Conclusions** | The management of AP patients in adherence to a PCT-based guideline resulted in a signiﬁcantly shorter duration of antibiotic use without compromising outcomes | | |

| **Paper:** Correlation between procalcitonin and intra-abdominal pressure and their role in prediction of the severity of acute pancreatitis  Pancreatology 2012  Bezmarevic M, *et al.* | | | |
| --- | --- | --- | --- |
| **Study type/ evidence level** | **Study details/limitations** | **Patients’ characteristics** | **Interventions** |
| **Study type:** Prospective cohort study  **Evidence level:** Moderate | ***Countries:*** Serbia  ***Centers:*** Military Medical Academy, Belgrade, Serbia  ***Setting:*** Patients with acute pancreatitis  ***Funding sources*:** None  ***Dropout rates:*** 0%  ***Limitations*:** Small sample size (Imprecision) | 51 patients with acute pancreatitis (AP), of which 29 had severe AP (SAP) | Patients were evaluated with the Acute Physiology And Chronic Health Evaluation (APACHE II) score, C-reactive protein (CRP) and PCT serum concentrations, and intra-abdominal pressure (IAP) at 24 h from admission. PCT was measured three times in the 1st week of the disease and three times afterward, while IAP was measured daily. PCT and IAP values correlated with each other and also compared with APACHE II score and CRP values |
| **Outcome Measurement/results** | Sensitivity/specificity of PCT, IAP, CRP, and APACHE II for predicting SAP | PCT, IAP, CRP values and APACHE II score at 24 h after hospital admission were signiﬁcantly elevated in patients with SAP. There was signiﬁcant correlation between PCT and IAP values measured at 24 h of admission, and between maximal PCT and IAP values. Sensitivity/speciﬁcity for predicting AP severity at 24 h after admission was 89%/69% for APACHE II score, 75%/86% for CRP, 86%/63% for PCT and 75%/77% for IAP | |
| **Conclusions** | Increased IAP was accompanied by increased PCT serum concentration in patients with AP. PCT and IAP can both be used as early markers of AP severity | | |

| **Paper:** The role of procalcitonin as a prognostic factor for acute cholangitis and infections in acute pancreatitis: a prospective cohort study from a European single center  HPB 2022  Alberti P, *et al.* | | | |
| --- | --- | --- | --- |
| **Study type/ evidence level** | **Study details/limitations** | **Patients’ characteristics** | **Interventions** |
| **Study type:** Prospective cohort study  **Evidence level:** Moderate | ***Countries:*** Spain  ***Centers:*** Hospital Val D’Hebron, Barcelona, Spain  ***Setting:*** Patient with acute pancreatitis  ***Funding sources*:** None  ***Dropout rates:*** 0%  ***Limitations*:** Small sample size (imprecision); PCT levels were only measured upon admission; changes of PCT were not taken into consideration; patients with acute kidney injury were include (false positives?) | 152 patients diagnosed with acute pancreatitis | PCT determination was tested on admission (first 72 h) |
| **Outcome Measurement/results** | Infections (biliary, extrapancreatic and infected pancreatic necrosis), need for antibiotics, urgent ERCP, and severity scores for AP | PCT >0.68 mg/dL had higher incidence of global infection, acute cholangitis, bacteraemia, infected pancreatic necrosis, use of antibiotics in general, and need for urgent ERCP. In the multivariate regressions analysis, PCT >0.68 mg/dL at admission demonstrated to be a strong risk factor for complications in acute pancreatitis | |
| **Conclusions** | PCT levels can be used as a reliable laboratory test to predict infections and the clinical severity of acute pancreatitis | | |

| **Paper:** A procalcitonin-based algorithm to guide antibiotic use in patients with acute pancreatitis (PROCAP): a single-centre, patient-blinded, randomized trial  Lancet gastroenterol hepatol 2022  Siriwardena AK, *et al.* | | | |
| --- | --- | --- | --- |
| **Study type/ evidence level** | **Study details/limitations** | **Patients’ characteristics** | **Interventions** |
| **Study type:** Randomized Controlled Trial  **Evidence level:** High | ***Countries:*** UK  ***Centers:*** Manchester Royal Infirmary, UK  ***Setting:*** Adult patients with a clinical diagnosis of acute pancreatitis  ***Funding sources*:** None  ***Dropout rates:*** 0%  ***Limitations*:** Single-center study | Eligible participants were aged 18 years or older and had a clinical diagnosis of acute pancreatitis | Participants were randomly assigned (1:1) to procalcitonin-guided care or usual care using web-based randomisation software. The randomisation sequence was stratified by disease severity and admission pathway, using variable block sizes of 4, 6, or 8. Patients, but not clinicians, were masked to group assignment. In the procalcitonin-guided care group, procalcitonin testing was conducted on days 0, 4, 7, and weekly thereafter. Guidance was to stop or not start antibiotics following a test value of less than 1.0 ng/mL and to start or continue antibiotics following a test value of 1.0 ng/mL or more |
| **Outcome Measurement/results** | The primary outcome was use of antibiotics during the index admission to hospital | 260 patients were enrolled and randomly assigned to a treatment group (132 to procalcitonin-guided care and 128 to usual care). 59 (45%) of patients in the procalcitonin-guided care group were prescribed antibiotics compared with 79 (63%) in the usual care group (adjusted risk difference -15.6% [95% CI -27.0 to -4.2]; p= 0.0071). The odds ratio for the treatment effect was 0.49 (95% CI 0.29 to 0.83; p= 0.0077). There was no significant difference between groups in terms of the number of clinical infections or hospital-acquired infections per patient. Four (3%) patients in the procalcitonin-guided care group and three (2%) patients in the usual care group died; all deaths were related to underlying severe pancreatitis. There was no difference in adverse events between the groups | |
| **Conclusions** | Procalcitonin-guided care can reduce antibiotic use without increasing infection or harm in patients with acute pancreatitis | | |

| **Paper:** The effect of procalcitonin and immature granulocyte ratio in predicting the development of acute necrotizing pancreatitis: evidence from 582 cases  European review for medical and pharmacological sciences 2022  Ugurlu ET, *et al.* | | | |
| --- | --- | --- | --- |
| **Study type/ evidence level** | **Study details/limitations** | **Patients’ characteristics** | **Interventions** |
| **Study type:** Not specified  **Evidence level:** Very low | ***Countries:*** Turkey  ***Centers:*** General Surgery Clinic of S.B. Şanliurfa Mehmet Akif Inan Training and Research Hospital, Turkey  ***Setting:*** Patients with acute pancreatitis  ***Funding sources*:** None  ***Dropout rates:*** Not applicable  ***Limitations*:** Retrospective design | 582 patients were divided into two groups according to CECT results; 525 (90.2%) were diagnosed with AEP, and 57 (9.7%) with ANP. Of the patients diagnosed with ANP, 33 (57.8%) were female, and 24 (42.1%) were male. The mean age was 57.9±21.05 in women and 58.06±17.34 in men | Procalcitonin, IG%, WBC, amylase, lipase, CRP, albumin, CRP/albumin levels were measured at the admission of the patient and at 48, 72 and 96 hours |
| **Outcome Measurement/results** | Predictive value of CRP and Procalcitonin for diagnosing acute necrotizing pancreatitis | CRP, procalcitonin, IG and CRP/ albumin were found to be significantly higher in ANP patients when compared to AEP (p<0.0001). According to the ROC analysis result, procalcitonin (AUROC: 0.999), IG% (AUROC: 0.995), WBC count (AUROC: 0.841), CRP (AUROC: 0.947), albumin (AUROC: 0.862), and CRP/albumin (AUROC: 0.946) ratio were markers that could be used for early prediction of ANP | |
| **Conclusions** | Procalcitonin levels can be easily accessible and effective markers in the early diagnosis of ANP and in the planning of treatment | | |

| **Paper:** Procalcitonin is a good tool to guide the duration of antibiotic therapy in patients with severe acute pancreatitis  Saudi Med J 2012  Qu R, *et al.* | | | |
| --- | --- | --- | --- |
| **Study type/ evidence level** | **Study details/limitations** | **Patients’ characteristics** | **Interventions** |
| **Study type:** Randomized controlled trial  **Evidence level:** Low | ***Countries:*** China  ***Centers:*** Guangdong Huizhou Municipal Central Hospital, China  ***Setting:*** Patients with severe acute pancreatitis  ***Funding sources*:** None  ***Dropout rates:*** 0%  ***Limitations*:** No methods reported for allocation, randomization and statistical power calculation | 71 patients with confirmed severe acute pancreatitis (SAP) | Patients were randomly assigned into 2 groups, including a PCT-guided group (study group) and a prophylactic antibiotic therapy (control group). Antibiotic therapy in the study group was not applied until the PCT value was >0.5ng/ml). Antibiotic therapy was discontinued if clinical signs and symptoms of infection improved and PCT was <0.5ng/ml over 3 days. In the control group, antibiotic therapy was administrated for 2 weeks, or antibiotic therapy was continued because of confirmed infection until clinical signs and symptoms of infection disappeared over 3 days |
| **Outcome Measurement/results** | Duration of intensive care unit stay, antibiotic treatment, length of hospitalization, cost of hospitalization, multi-organ dysfunction syndrome (MODS), mortality | In the study group (35 patients), the duration of antibiotic therapy and hospitalization was significantly shorter than the control group (36 patients) (10.89±2.85 versus 16.06±2.48 days, p<0.001, and 16.66±4.02 days versus 23.81±7.56 days, p<0.001) without negative clinical effects; the cost of hospitalization was significantly lower | |
| **Conclusions** | Procalcitonin is a helpful tool for guiding the duration of antibiotic treatment in patients with severe acute pancreatitis | | |

**8. Supplemental Files Table 7.** **Supplementary Table Report Template Question 4.**

| **Paper:** Three initial diets for management of mild acute pancreatitis: a meta-analysis  World journal of gastroenterology 2011  Meng WB, *et al.* | | | |
| --- | --- | --- | --- |
| **Study type/ evidence level** | **Study details/limitations** | **Patients’ characteristics** | **Interventions** |
| **Study type:** Meta-analysis of Randomized Controlled Trials (RCTs)  **Evidence level:** High | ***Countries:*** Not applicable  ***Centers:*** Not applicable  ***Setting:*** Patients with mild acute pancreatitis  ***Funding sources*:** Not reported  ***Dropout rates:*** Not applicable  ***Limitations*:** Small sample size | Patients with mild acute pancreatitis | Randomized controlled trials (RCTs) that compared non-liquid with clear-liquid diets in patients with mild acute pancreatitis were included |
| **Outcome Measurement/results** | Length of hospital stay; recurrence of the pain after refeeding | Three RCTs involving a total of 362 participants were included in the final analysis. Compared to the liquid diet, the non-liquid diet significantly decreased the length of hospitalization [mean difference (MD) 1.18, 95% CI 0.82-1.55; P ﹤0.00001] and total length of hospitalization (MD 1.31, 95% CI 0.45-2.17; P = 0.003). The subgroup analysis showed solid diet was more favorable than a clear liquid diet in the length of hospitalization, with a pooled MD being -1.05 (95% CI -1.43 to -0.66; P ﹤0.00001). However, compared with the clear liquid diet, both soft and solid diets did not show any significant differences in the recurrence of pain after refeeding, either alone [relative risk (RR) 0.95; 95% CI 0.51-1.87; P = 0.88] and (RR 1.22; 95% CI 0.69-2.16; P = 0.49), respectively, or analyzed together as non-liquid diet (RR 0.80; 95% CI 0.47-1.36; P = 0.41) | |
| **Conclusions** | The non-liquid soft or solid diet did not increase pain recurrence after re-feeding, compared with the clear-liquid diet. The non-liquid diet reduced hospitalization | | |

**Research question 4. *Should early (within 24 hours) oral feeding as tolerated vs. keeping the patient nil per os be used for patients with mild acute biliary pancreatitis (if tolerated)?***

| **Paper:** Optimal initial diet in mild acute pancreatitis: a comprehensive meta-analysis of randomized controlled trials  Pancreatology 2022  Chowdhury AR, *et al.* | | | |
| --- | --- | --- | --- |
| **Study type/ evidence level** | **Study details/limitations** | **Patients’ characteristics** | **Interventions** |
| **Study type:** Meta-analysis of RCTs  **Evidence level:** High | ***Countries:*** Not applicable  ***Centers:*** Not applicable  ***Setting:*** Patients with mild acute pancreatitis  ***Funding sources*:** The authors acknowledged support from Elizabeth and Jerry Paul  ***Dropout rates:*** Not applicable  ***Limitations*:** A relatively small number of RCTs, significant heterogeneity, differences in diet composition | 305 patients with mild acute pancreatitis | Patients were randomized to an immediate full solid diet (FSD) or sequential advancement |
| **Outcome Measurement/results** | Length of hospital stay (LOHS), post-refeeding abdominal pain, tolerance of diet | Patients who were initiated on an FSD had a signiﬁcant reduction in length of hospital stay (Standardized Mean Difference (SMD) 0.52 [95% CI -0.69, 0.36]). There was no difference in post-refeeding abdominal pain, tolerance of diet, or necessity to cease diet between the two groups. Sub-analysis of three studies that initiated FSD early reduced total length of hospital stay (OR -0.95 [95% CI -1.26, 0.65]) compared to those who received graded diet advancement as well as a higher likelihood of tolerating the assigned diet (OR 6.8 [95% CI 1.2, 39.2]) | |
| **Conclusions** | Initiation of FSD reduces total LOHS in patients with mild AP and does not increase post-refeeding abdominal pain. These ﬁndings support an initial solid diet for AP and consideration of feeding within the ﬁrst 24 h | | |

| **Paper:** Effects of immediate or early oral feeding on acute pancreatitis: a systematic review and meta-analysis  Pancreatology 2022  Yao Q, *et al.* | | | |
| --- | --- | --- | --- |
| **Study type/ evidence level** | **Study details/limitations** | **Patients’ characteristics** | **Interventions** |
| **Study type:** Meta-analysis of RCTs  **Evidence level:** High | ***Countries:*** Not applicable  ***Centers:*** Not applicable  ***Setting:*** Patients with mild and moderate acute pancreatitis  ***Funding sources*:** National Natural Science Foundation of China under Grant 81860123  ***Dropout rates:*** Not applicable  ***Limitations*:** Included seven single-center studies; timing of oral refeeding was not available in two studies | 748 patients with mild to moderate acute pancreatitis (AP) | Immediate or early versus delayed oral feeding on mild and moderate AP |
| **Outcome Measurement/results** | Costs, length of hospital stay, mortality, pain relapse, feeding intolerance, AP progression, complications | Patients in IOR (Immediate or early Oral Refeeding) group had fewer costs [SMD -0.83, 95%CI (-1.17, -0.5), P < 0.001] and shorter LOS [SMD -1.01, 95%CI (-1.17, -0.85), P < 0.001] than the DOR (Delayed Oral Refeeding) group patients. There was no difference in mortality [RR 0.54, 95%CI (0.11, 2.62), P =0.44], pain relapse rate [RR 0.58, 95%CI (0.25, 1.35), P =0.27], feeding intolerance rate [RR 0.61, 95%CI (0.28, 1.3), P =0.2], AP progression rate [RR 0.21, 95%CI (0.04, 1.07), P =0.06] and overall complications rate [RR 0.41, 95%CI (0.17, 1.01), P =0.05] between the IOR and DOR groups | |
| **Conclusions** | Immediate oral refeeding could reduce the length of hospital stay and costs without increasing adverse events in mild to moderate AP | | |

| **Paper:** Incidence and predictors of oral feeding intolerance in acute pancreatitis: a systematic review, meta-analysis, and meta-regression  Clinical nutrition 2016  Bevan MG, *et al.* | | | |
| --- | --- | --- | --- |
| **Study type/ evidence level** | **Study details/limitations** | **Patients’ characteristics** | **Interventions** |
| **Study type:** Meta-analysis of RCTs and observational studies  **Evidence level:** Moderate | ***Countries:*** Not applicable  ***Centers:*** Not applicable  ***Setting:*** Patients with acute pancreatitis  ***Funding sources*:** Not reported  ***Dropout rates:*** Not applicable  ***Limitations*:** Heterogeneity | 2024 patients met the inclusion criteria, 1550 of which were suitable for meta-analysis | Incidence and predictor data were meta-analysed and possible confounders were investigated by meta-regression analysis |
| **Outcome Measurement/results** | Incidence of oral feeding intolerance, the effect of confounders, and the best predictors of oral feeding intolerance | The incidence of oral feeding intolerance was 16.3%, and was not affected by WHO region, age, sex, or aetiology of acute pancreatitis. Nine of the 22 studies investigated a total of 62 different predictors of oral feeding intolerance. Serum lipase level prior to refeeding, pleural effusions, (peri)pancreatic collections, Ranson score, and Balthazar score were found to be statistically signiﬁcant in meta-analyses | |
| **Conclusions** | Oral feeding intolerance affects approximately 1 in 6 patients with acute pancreatitis worldwide. Serum lipase levels are potentially a clinically useful threshold to identify patients at high risk of developing oral feeding intolerance | | |

| **Paper:** A meta-analysis of early oral refeeding and quickly increased diet for patients with mild acute pancreatitis  Saudi Journal of Gastroenterology 2018  Zhang J, *et al.* | | | |
| --- | --- | --- | --- |
| **Study type/ evidence level** | **Study details/limitations** | **Patients’ characteristics** | **Interventions** |
| **Study type:** Meta-analysis of observational studies  **Evidence level:** Low | ***Countries:*** Not applicable  ***Centers:*** Not applicable  ***Setting:*** Patients with mild acute pancreatitis  ***Funding sources*:** None  ***Dropout rates:*** Not applicable  ***Limitations*:** Non-randomized studies | The four analyzed trials included 388 patients with mild acute pancreatitis | Early oral refeeding (EORF) and quickly increasing diet (QID) compared with a traditional oral refeeding strategy. The criteria for traditional opinion for starting oral refeeding were as follows: abdominal pain was controlled, the gastrointestinal function was recovered, and pancreatic enzymes became normalized (conventional oral refeeding [CORF]); a clear liquid diet was first performed for the stepwise refeeding protocol (SID). For refeeding, according to the latest opinion, oral refeeding was performed earlier (EORF) and started with a full solid diet or a soft diet (quickly increasing diet [QID]) |
| **Outcome Measurement/results** | Relapse of abdominal pain, nausea/vomiting, and length of hospital stay (LOHS) | EORF significantly decreased the LOHS (mean deviation [MD] −1.97; 95% confidence interval (CI) −3.32 to −0.62; P = 0.004), and there was no significant difference in the relapse of abdominal pain (relative risk [RR] 1.17; 95% CI 0.69–2.00; P = 0.56) or nausea/vomiting (RR 1.30; 95% CI 0.19–8.82; P = 0.79) when compared with conventional oral refeeding. For the oral refeeding material group, there was no significant difference for relapse of abdominal pain (RR 0.86; 95% CI 0.53–1.40; P = 0.54), nausea/vomiting (risk difference −0.01; 95% CI −0.19–0.18; P = 0.94), or LOHS (MD −0.88; 95% CI −2.24–0.48; P = 0.20) between the QID and stepwise increasing diet groups | |
| **Conclusions** | Pure EORF or QID caused no damage to patients with mild acute pancreatitis, and EORF could significantly decrease the LOHS | | |

| **Paper:** Optimal timing of oral refeeding in mild acute pancreatitis. Results of an open randomized multicenter trial  Pancreas 2010  Teich N, *et al.* | | | |
| --- | --- | --- | --- |
| **Study type/ evidence level** | **Study details/limitations** | **Patients’ characteristics** | **Interventions** |
| **Study type:** Randomized Controlled Trial  **Evidence level:** Moderate | ***Countries:*** Germany  ***Centers:*** Multicenter study (all the centers were German)  ***Setting:*** Patients with mild acute pancreatitis  ***Funding sources*:** Novartis Research Award BGraduiertenstipendium derNovartis-Stiftung fur therapeutische Forschung  ***Dropout rates:*** 10% in the study group and 32% in the control group  ***Limitations*:** Imprecision | 143 patients with mild acute pancreatitis | Patients were randomized to the Lipase directed (LIP) (n = 74) and the self-selected PAT (n = 69) group. In the PAT group, the patients restarted eating through self-selection. In the LIP group, serum lipase had to normalize before eating |
| **Outcome Measurement/results** | Time to oral nutrition (fasting time), length of hospital stay | The mean time between admission and oral nutrition was 2 days (interquartile range [IQR], 1-3) in the PAT group and 3 days (IQR, 2-4) in the LIP group (P< 0.005). Before and after the ﬁrst meal, the mean visual analog scale (VAS) was +3.14 mm (±11.5 mm) in the PAT group and +2.85 mm (±16.4) in the LIP group (P= 0.597). The length of hospital stay was 7 days (median; IQR, 5-10.5) in the PAT group and 8 days (median; IQR, 5.75-12) in the LIP group (P= 0.315) | |
| **Conclusions** | Patients with self-selected eating were able to restart eating 1 day earlier, with no significant difference in postprandial abdominal pain or in the length of hospital stay | | |

| **Paper:** Clinical trial: oral feeding with a soft diet compared with clear liquid diet as initial meal in mild acute pancreatitis  Alimentary Pharmacology & Therapeutics 2008  Sathiaraj E, *et al.* | | | |
| --- | --- | --- | --- |
| **Study type/ evidence level** | **Study details/limitations** | **Patients’ characteristics** | **Interventions** |
| **Study type:** Randomized Controlled Trial  **Evidence level:** High | ***Countries:*** India  ***Centers:*** Asian Institute of Gastroenterology, Somajiguda, Hyderabad, India  ***Setting:*** Mild acute pancreatitis  ***Funding sources*:** None  ***Dropout rates:*** 0%  ***Limitations*:** - | One hundred and one patients with mild acute pancreatitis | Patients were randomized to receive either a clear liquid diet or soft diet when oral feeding was initiated |
| **Outcome Measurement/results** | Frequency of pain, total and post-refeeding length of hospitalization, and dietary intake were monitored. Hospital discharge was decided by the medical team without input from the study coordinators | A statistically signiﬁcant decrease in the length of hospitalization (total and post-refeeding) of a median of 2 days was seen in patients receiving a soft diet (P < 0.001). No signiﬁcant difference in the need for cessation of diet because of pain was observed between the two groups. Patients initiated on a soft diet consumed signiﬁcantly more calories and fats on study day 1 (P < 0.001) | |
| **Conclusions** | Oral refeeding with a soft diet in patients with mild acute pancreatitis can be considered safe and can result in shorter length of hospitalization | | |

| **Paper:** Hunger-based versus conventional oral feeding in moderate and severe acute pancreatitis: a randomized controlled trial  Digestive disease and science 2021  Rai A, *et al.* | | | |
| --- | --- | --- | --- |
| **Study type/ evidence level** | **Study details/limitations** | **Patients’ characteristics** | **Interventions** |
| **Study type:** Randomized Controlled Trial  **Evidence level:** High | ***Countries:*** India  ***Centers:*** Department of Surgery, Jawaharlal Institute of Postgraduate Medical Education and Research, Pondicherry, India  ***Setting:*** Patients with moderate and severe acute pancreatitis  ***Funding sources*:** Not reported  ***Dropout rates:*** 0%  ***Limitations*:** Low fat diet and probiotics were not investigated | 110 patients with moderate and severe acute pancreatitis | Patients were randomized into hunger-based feeding and conventional feeding groups. Patients in the hunger-based feeding group commenced feeding once they felt hungry, and in the conventional feeding group after normalization of biochemical parameters and resolution of symptoms |
| **Outcome Measurement/results** | Length of hospitalization, fasting duration, feed intolerance, incidence of infective morbidities and invasive procedures | Hunger-based feeding and conventional feeding group included 56 and 54 patients, respectively. Hunger-based feeding led to a decrease in the length of hospitalization (6.3 days in hunger-based feeding vs. 7.3 days in the conventional feeding group, P= 0.041) and fasting duration (1.6 days in hunger-based feeding vs. 2.7 days in the conventional feeding group, P= 0.001). The incidence of feed intolerance (P= 0.098), infective morbidities, and invasive non-surgical procedures were similar in both the groups | |
| **Conclusions** | Hunger-based feeding significantly reduces length of hospitalization and fasting duration in cases of moderate and severe acute pancreatitis without any significant rise in the incidence of complications | | |

| **Paper:** A full solid diet as the initial meal in mild acute pancreatitis is safe and results in a shorter length of hospitalization. Results from a prospective, randomized, controlled, double-blind clinical trial  Journal of clinical gastroenterology 2010  Moraes JMM, *et al.* | | | |
| --- | --- | --- | --- |
| **Study type/ evidence level** | **Study details/limitations** | **Patients’ characteristics** | **Interventions** |
| **Study type:** Randomized controlled trial  **Evidence level:** Moderate | ***Countries:*** Brazil  ***Centers:*** Division of Gastroenterology, Department of Medicine, University Hospital of the Federal University of Juiz de Fora, University of Juiz de Fora School of Medicine, Minas Gerais, Brazil  ***Setting:*** Patients with mild acute pancreatitis  ***Funding sources*:** Partly supported by a clinical research fund from the CNPq and FAPEMIG, Brazil  ***Dropout rates:*** 0%  ***Limitations*:** Doubts on the allocation concealment | 210 patients with mild acute pancreatitis | Patients were randomized to receive 1 of 3 diets (clear liquid, soft, or full solid) as the initial meal during oral refeeding |
| **Outcome Measurement/results** | Relapse of pain (primary endpoint), dietary intake, length of hospital stay (LOH- secondary endpoint), and 7 days post-discharge to record pain relapse rates | There was no diﬀerence in pain relapse rates during refeeding between the 3 diet arms (P= 0.80). Patients initiated on a full solid diet consumed signiﬁcantly more calories and fats on trial days 1 and 2 (P< 0.001). A shorter LOH (median of –1.5 d) was observed among patients receiving a full solid diet without abdominal pain relapse (P= 0.000) | |
| **Conclusions** | Oral refeeding with a full solid diet in mild acute pancreatitis is well tolerated and results in a shorter length of hospital stay, without abdominal pain relapse | | |

| **Paper:** Early oral refeeding wisdom in patients with mild acute pancreatitis  Pancreas 2013  Li J, *et al.* | | | |
| --- | --- | --- | --- |
| **Study type/ evidence level** | **Study details/limitations** | **Patients’ characteristics** | **Interventions** |
| **Study type:** Randomized Controlled Trial  **Evidence level:** Low | ***Countries:*** China  ***Centers:*** Pancreatic Diseases Research Group, Department of Integrated Traditional and Western Medicine, West China Hospital, Sichuan University, Chengdu, China  ***Setting:*** Patients with mild acute pancreatitis  ***Funding sources*:** National Natural Science Foundation of China No. 30973711 and National Institute for Health Research of UK and UK/China Postgraduate Scholarships for Excellence  ***Dropout rates:*** 0%  ***Limitations*:** No allocation method was reported, and no information about the statistical power | 149 eligible patients with mild acute pancreatitis were included in the study | 75 Patients with EORF (started oral feeding once they subjectively felt hungry) were compared with 74 patients receiving routine oral refeeding (RORF) |
| **Outcome Measurement/results** | Time interval between disease onset and initiation of oral refeeding, total length of hospitalization (LOH), post-refeeding LOH, and adverse gastrointestinal events | Patients in the EORF group started refeeding significantly earlier than those in the RORF group (4.56±1.53 vs 6.75±2.29 days; P< 0.05). Moreover, patients in the EORF group had significantly shorter total (6.8±2.1 vs 10.4±4.1 days; P< 0.01) and post-refeeding LOH (2.24±0.52 vs 3.27±0.61 days; P< 0.01). There was no significant difference in adverse gastrointestinal events between the 2 groups | |
| **Conclusions** | In patients with mild AP, EORF, with the subjective feeling of hunger, is safe, feasible, and reduces LOH | | |

| **Paper:** A prospective, randomized trial of clear liquids versus low-fat solid diet as the initial meal in mild pancreatitis  Clinical gastroenterology and hepatology 2007  Jacobson BC, *et al.* | | | |
| --- | --- | --- | --- |
| **Study type/ evidence level** | **Study details/limitations** | **Patients’ characteristics** | **Interventions** |
| **Study type:** Randomized Controlled Trial  **Evidence level:** High | ***Countries:*** USA  ***Centers:*** Boston University Medical Center; Brigham and Women’s Hospital; Harvard School of Public Health, Boston, Massachusetts  ***Setting:*** Patients with mild acute pancreatitis  ***Funding sources*:** Supported in part by an American College of Gastroenterology Clinical Research Award and by a National Institutes of Health/National Institute of Diabetes & Digestive & Kidney Diseases career development award (K08 DK070706 to B.C.J.)  ***Dropout rates:*** 0%  ***Limitations*:** Large number of excluded patients | 121 patients with mild acute pancreatitis | Low-fat solid diet (LFSD) versus clear liquid diet (CLD) |
| **Outcome Measurement/results** | Recurrence of pain, need to stop feeding, post-refeeding length of hospital stay (LOH) (primary endpoint), re-admission rates | 121 patients were randomized (66 to CLD and 55 to LFSD). The number of patients requiring cessation of feeding because of pain or nausea was similar in both groups (6% for CLD, 11% for LFSD; P= 0.51). The median LOH after refeeding was identical in both groups (1-day interquartile range, 1–2; P= 0.77). Patients in the LFSD arm consumed signiﬁcantly more calories and grams of fat than those in the CLD arm during their ﬁrst meal and on study day 1. There was no difference in the 28-day re-admission rates between the 2 arms | |
| **Conclusions** | Initiating oral nutrition after mild acute pancreatitis with an LFSD is safe and provides more calories than a CLD | | |

| **Paper:** Immediate oral refeeding in patients with mild and moderate acute pancreatitis. A multicenter, randomized controlled trial (PADI trial)  Annals of surgery 2021  Ramìrez-Maldonado E, *et al.* | | | |
| --- | --- | --- | --- |
| **Study type/ evidence level** | **Study details/limitations** | **Patients’ characteristics** | **Interventions** |
| **Study type:** Randomized Controlled Trial  **Evidence level:** High | ***Countries:*** Spain  ***Centers:*** Barcelona, Tarragona  ***Setting:*** Patients with mild and moderate acute pancreatitis  ***Funding sources*:** Research grant awarded from the ‘‘Societat Catalana de Cirurgia”  ***Dropout rates:*** 4.2% in the experimental group and 5% in the control group  ***Limitations*:** - | 131 patients with a mild and moderate acute pancreatitis | Patients were randomized into 2 treatment groups: immediate oral refeeding (IORF) and conventional oral refeeding (CORF). The IORF group (low-fat-solid diet initiated immediately after hospital admission) was compared to CORF group (progressive oral diet was restarted when clinical and laboratory parameters had improved) |
| **Outcome Measurement/results** | Length of hospital stay (LOS) (primary endpoint), pain relapse, diet intolerance, complications, hospital costs | The mean LOS for the IORF and CORF groups was 3.4 (SD 1.7) and 8.8 (SD 7.9) days, respectively (P< 0.001). In the CORF group alone, pain relapse rate was 16%. There were fewer complications (8% vs 26%), and health costs were twice as low, with a savings of 1325.7 Euro/patient in the IORF than CORF group | |
| **Conclusions** | IORF is safe and feasible in mild and moderate AP, resulting in significantly shorter LOS and cost savings, without causing adverse effects or complications | | |

| **Paper:** Efficacy and safety of immediate oral intake in patients with mild acute pancreatitis: a randomized controlled trial  Nutrition 2020  Horibe M, *et al.* | | | |
| --- | --- | --- | --- |
| **Study type/ evidence level** | **Study details/limitations** | **Patients’ characteristics** | **Interventions** |
| **Study type:** Randomized Controlled Trial  **Evidence level:** High | ***Countries:*** Japan  ***Centers:*** Keio University School of Medicine, Tokyo, Japan  ***Setting:*** Patients with mild acute pancreatitis  ***Funding sources*:** Keio University Grant-in-Aid for Encouragement of Young Medical Scientists  ***Dropout rates:*** 0%  ***Limitations*:** Single-institution study | 26 patients with mild acute pancreatitis | Immediate feeding (IMF) group was permitted with oral intake of low-fat (15 g/d) solid food. In the standard food (STF) group, patients received gradually increasing amounts of dietary fat. Twenty-six patients were randomized, with 13 allocated to each group |
| **Outcome Measurement/results** | The primary outcome was the period between diagnosis and recovery from AP. The cost and rate of progression to severe disease were evaluated as secondary outcomes | The IMF group (mean recovery days: 2±1) recovered signiﬁcantly earlier (mean difference in recovery days: 6.3; 95% conﬁdence interval [CI], 4.8-7.9; P< 0.001) than the STF group (mean recovery days: 8.3±2.3), with a lower overall treatment cost P= 0.034). The IMF group showed a lower (although not significant) rate of progression to severe AP (IMF, 0%; STF, 15.3%; P = 0.48) | |
| **Conclusions** | The initial treatment strategy for mild AP should be altered from the gradual introduction of oral feeding upon the absence of pain to immediate oral nutrition with opioid analgesics, to improve treatment efﬁcacy and reduce treatment cost | | |

| **Paper:** Immediate oral feeding in patients with mild acute pancreatitis is safe and may accelerate recovery – a randomized clinical study  Clinical nutrition 2007  Eckerwall GE, *et al.* | | | |
| --- | --- | --- | --- |
| **Study type/ evidence level** | **Study details/limitations** | **Patients’ characteristics** | **Interventions** |
| **Study type:** Randomized Controlled Trial  **Evidence level:** High | ***Countries:*** Sweden  ***Centers:*** Department of Surgery, Clinical Sciences Lund, Lund University Hospital, Sweden  ***Setting:*** Patients with mild acute pancreatitis  ***Funding sources*:** Swedish Nutrition Foundation, Swedish Research Council (Grant no. 11246), and Foundation for Gut and Intestinal Research  ***Dropout rates:*** 0%  ***Limitations*:** The design did not include blinding | 60 patients with mild acute pancreatitis | Two treatment groups, fasting (30 patients) or immediate oral feeding (30 patients) (patients were immediately allowed to drink and eat freely as tolerated) |
| **Outcome Measurement/results** | Pancreas-speciﬁc amylase, systemic inﬂammatory response, feasibility and length of hospital stay (LOHS) | No signiﬁcant differences were seen between the groups concerning levels of amylase, CRP, leukocytes, abdominal pain or number of gastrointestinal symptoms. The LOHS was signiﬁcantly shorter in the oral feeding group (4 vs. 6 days; p< 0.05) | |
| **Conclusions** | In mild acute pancreatitis, immediate oral feeding is feasible and safe and may accelerate recovery without adverse gastrointestinal events | | |

| **Paper:** Non-inferiority comparative clinical trial between early oral refeeding and usual oral refeeding in predicted mild acute biliary pancreatitis  BMC gastroenterology 2020  Lozada-Hernàndez EE, *et al.* | | | |
| --- | --- | --- | --- |
| **Study type/ evidence level** | **Study details/limitations** | **Patients’ characteristics** | **Interventions** |
| **Study type:** Randomized Controlled Trial  **Evidence level:** Moderate | ***Countries:*** Mexico  ***Centers:*** Department of Surgery and Clinical Research, Hospital Regional de Alta Especialidad del Bajío, Circuito Quinta los Naranjos # 145 B. Colonia Quinta los Naranjos, León, Guanajuato, Mexico  ***Setting:*** Patients with predicted mild acute pancreatitis  ***Funding sources*:** None  ***Dropout rates:*** 3.2%  ***Limitations*:** Unclear allocation concealment; lack of blinding | 124 patients with predicted mild acute pancreatitis were randomized | A total of 124 patients were randomized, and 120 were included (61 in the EOR group and 59 in the UOR group). EOR (Early Oral Refeeding), UOR (Usual Oral Refeeding) |
| **Outcome Measurement/results** | Pancreatic lipase levels, the systemic inflammatory response (concentrations of leukocytes), feasibility (evaluated by abdominal pain recurrence), the presence and recurrence of gastrointestinal symptoms and the length of hospital stay | Two patients in the EOR group experienced pain relapse (3.2%), and four patients in the UOR group experienced pain relapse (6.77%) after oral refeeding (p= 0.379). The presence of nausea or vomiting after the onset of oral refeeding was not different between the two groups (p= 0.293). The onset of oral refeeding was approximately 48 h later in the UOR group. The length of hospital stay was 5 days in the EOR group and 8 days in the UOR group (p= 0.042), and this difference was also manifested in higher hospital costs in the UOR group (p= 0.0235) | |
| **Conclusions** | Compared with usual oral refeeding, early oral refeeding is safe in predicted mild acute biliary pancreatitis patients, does not cause adverse gastrointestinal events, and reduces the length of hospital stay and costs | | |

| **Paper:** Early and/or immediately full caloric diet versus standard refeeding in mild acute pancreatitis: a randomized open-label trial  Pancreatology 2014  Larino-Noia J, *et al.* | | | |
| --- | --- | --- | --- |
| **Study type/ evidence level** | **Study details/limitations** | **Patients’ characteristics** | **Interventions** |
| **Study type:** Randomized Controlled Trial  **Evidence level:** High | ***Countries:*** Spain  ***Centers:*** University Hospital of Santiago de Compostela, Spain  ***Setting:*** Patients with mild acute pancreatitis  ***Funding sources*:** None  ***Dropout rates:*** 0%  ***Limitations*:** Lack of blinding | 72 patients with mild acute pancreatitis (AP) were randomized into four different refeeding protocols | Groups 1 and 2 received a stepwise increasing diet for three days while 3 and 4 received an immediately full caloric, low-fat diet. Groups 2 and 4 started refeeding early (once bowel sounds returned), and 1 and 3 started at a standard time (bowel sounds present, no abdominal pain, no fever, leucocytes and pancreatic enzymes decreasing) |
| **Outcome Measurement/results** | Main outcomes measurements were LOHS and tolerance (ability to ingest >50% of meals without severe pain, nausea or AP relapse) | 72 patients were randomized (median age 60 years, range 24-85, 33 male). LOHS was signiﬁcantly reduced after early refeeding (median 5 versus 7 days (p< 0.001)) but not in patients receiving an immediate full caloric diet, compared to standard management (6 versus 6 days (p< 0.12)). There was no difference in refeeding tolerance comparing immediately full caloric diet versus stepwise increasing diet (31/35 (89%) versus 33/37 (89%) patients tolerating the treatment, p= 1.00) or early versus standard time for refeeding (33/37 (89%) versus 31/35 (89%), (p= 1.00)) | |
| **Conclusions** | Refeeding after AP when bowel sounds are present with immediate full caloric diet is safe and well tolerated. Early refeeding shortens the length of hospital stay | | |

| **Paper:** Early oral refeeding based on hunger in moderate and severe acute pancreatitis. A prospective controlled, randomized clinical trial  Nutrition 2015  Zhao XL, *et al.* | | | |
| --- | --- | --- | --- |
| **Study type/ evidence level** | **Study details/limitations** | **Patients’ characteristics** | **Interventions** |
| **Study type:** Randomized Controlled Trial  **Evidence level:** Moderate | ***Countries:*** China  ***Centers:*** Pancreatic Diseases Research Group, Department of Integrative Medicine, West China Hospital, Sichuan University, Chengdu, China  ***Setting:*** Patients with moderate or severe acute pancreatitis  ***Funding sources*:** National Natural Science Foundation of China no. 81374042  ***Dropout rates:*** 4.3% in the experimental group and 6.6% in the control group  ***Limitations*:** No information on the allocation concealment and sample size calculation | 146 eligible patients with moderate or severe acute pancreatitis (AP) were included | Patients were randomized to the EORF (n= 70) or CORF (n= 76) group. EORF (Early Oral Refeeding), CORF (Conventional Oral Refeeding) |
| **Outcome Measurement/results** | Length of hospitalization, duration of fasting, adverse events | The total length of hospitalization (13.7±5.4 d versus 15.7±6.2 d; P= 0.0398) and duration of fasting (8.3±3.9 d versus 10.5±5.1 d; P= 0.0047) were shorter in the EORF group than in the CORF group. There was no difference in the number of adverse events or complications between the two groups. The mean blood glucose level after oral refeeding was higher in the EORF group than in the CORF group (P= 0.0030) | |
| **Conclusions** | This controlled, randomized clinical trial conﬁrmed the effectiveness and feasibility of EORF based on hunger in patients with moderate or severe AP. EORF could shorten the length of hospitalization in patients with moderate or severe AP | | |

**9. Supplemental Files Table 8.** **Supplementary Table Report Template Questions 5-6.**

| **Paper:** A systematic review and meta-analysis of the effect of total parenteral nutrition and enteral nutrition on the prognosis of patients with acute pancreatitis  Annals of palliative medicine 2021  Liu M, *et al.* | | | |
| --- | --- | --- | --- |
| **Study type/ evidence level** | **Study details/limitations** | **Patients’ characteristics** | **Interventions** |
| **Study type:** Systematic review and meta-analysis of Randomized Controlled Trials  **Evidence level:** High | ***Countries:*** Not applicable  ***Centers:*** Not applicable  ***Setting:*** Patients with severe acute pancreatitis  ***Funding sources*:** None  ***Dropout rates:*** Not applicable  ***Limitations*:** Heterogeneity (addressed through sensitivity analyses) | 699 patients with severe acute pancreatitis | Total parenteral nutrition (TPN) and enteral nutrition (TEN) |
| **Outcome Measurement/results** | Infection rates, multiple organ failure, mortality, length of hospital stay | The obtained statistic value [odds ratio (OR) 0.25, 95% confidence interval (CI): 0.10 to 0.62] showed TEN had less infection rate than TPN (P= 0.003). A total of 8 studies (654 participants) reported the incidence rate indicators of multiple organ failure rate indicator; the obtained statistic value (OR 0.50, 95% CI: 0.24 to 1.08) showed no statistical difference between TEN and TPN (P> 0.05). A total of 7 studies (550 participants) reported the mortality indicators. The obtained statistic value (OR 0.59, 95% CI: 0.37 to 0.94) showed TEN had less mortality than TPN (P= 0.03). A total of 3 studies reported the length of hospital stay indicators. The obtained statistic value [mean difference (MD) −4.18, 95% CI: −5.07 to −3.30] showed the length of hospital stay for TEN was shorter than TPN (P< 0.001) | |
| **Conclusions** | Compared with TPN, TEN can reduce the incidence of infection, reduce the development of multiple organ failure, reduce mortality, and shorten the length of hospital stay in patients with severe acute pancreatitis (SAP) | | |

**Research question 5. *Should enteral nutrition (EN) vs. total parenteral nutrition (TPN) be used for the prevention of gut failure and infectious complications in patients with acute biliary pancreatitis and the inability to feed orally?* Research question 6. *Should early enteral nutrition (eEN) within 48 hours vs. delayed enteral nutrition (dEN) beyond 48 hours be used in patients with severe acute biliary pancreatitis and inability to feed orally?***

| **Paper:** Meta-Analysis of Early Nutrition: The Beneﬁts of Enteral Feeding Compared to a Nil Per Os Diet Not Only in Severe, but Also in Mild and Moderate Acute Pancreatitis  International Journal of Molecular Science 2016  Màrta K*, et al.* | | | |
| --- | --- | --- | --- |
| **Study type/ evidence level** | **Study details/limitations** | **Patients’ characteristics** | **Interventions** |
| **Study type:** Systematic review and meta-analysis  **Evidence level:** Very low | ***Countries:*** Not applicable  ***Centers:*** Not applicable  ***Setting:*** Patients with mild, moderate, and severe acute pancreatitis  ***Funding sources*:** Hungarian Scientiﬁc Research Fund (K116634 to PH) and the Momentum Grant of the Hungarian Academy of Sciences (LP2014-10/2014 to PH)  ***Dropout rates:*** Not applicable  ***Limitations*:** Non-randomized studies, heterogeneity, lack of bias assessment | Patients with various degrees of acute pancreatitis | Enteral nutrition versus nil per os diet |
| **Outcome Measurement/results** | Mortality, multiorgan failure, and intervention | All of the primary endpoints investigated showed that EN is beneﬁcial vs. NPO in SAP. In MAP, all of the six articles found merit in EN. Analyses of the primary endpoints did not show signiﬁcant differences between the groups; however, analyzing the 17 endpoints together showed a signiﬁcant difference in favor of EN vs. NPO | |
| **Conclusions** | EN is beneﬁcial compared to a nil per os diet not only in severe, but also in mild and moderate AP | | |

| **Paper:** Early Enteral Nutrition within 24 Hours or between 24 and 72 Hours for Acute Pancreatitis: Evidence Based on 12 RCTs  Medical science monitor 2014  Li X, *et al.* | | | |
| --- | --- | --- | --- |
| **Study type/ evidence level** | **Study details/limitations** | **Patients’ characteristics** | **Interventions** |
| **Study type:** Systematic review and meta-analysis  **Evidence level:** Moderate | ***Countries:*** Not applicable  ***Centers:*** Not applicable  ***Setting:*** Patients with severe acute pancreatitis or predicted severe acute pancreatitis  ***Funding sources*:** Departmental sources  ***Dropout rates:*** Not applicable  ***Limitations*:** The analysis included also patients with mild pancreatitis | A total of 625 participants were included in the 12 studies, with 301 patients in the early enteral nutrition (EEN) group and 324 in the control group (delayed enteral nutrition and total parenteral nutrition – TPN -) | Studies included in this meta-analysis had to fulfill the following criteria: (1) randomized comparative trials (RCTs); (2) consecutive patients with acute pancreatitis; (3) patients were randomized assigned to experimental EEN group initiated within 72 h of admission or control group with TPN or DEN (beyond 72 h) |
| **Outcome Measurement/results** | Association between intervention and complications, including pancreatic infection, mortality, hyperglycemia, organ failure, and catheter-related septic complications | Pooled analysis showed that EEN, but not TPN or delayed enteral nutrition (DEN), is associated with reduced risk of pancreatic infection, mortality, organ failure, hyperglycemia, and catheter-related septic complications. EEN within 24 h of admission presented significantly better outcome in morality than EEN between 24 and 72 h | |
| **Conclusions** | If severe pancreatitis patients are reasonably expected to have high compliance to EN therapy, it could be considered as early as possible | | |

| **Paper:** Enteral versus parenteral nutrition for acute pancreatitis  The Cochrane 2010  Al-Omran M, *et al.* | | | |
| --- | --- | --- | --- |
| **Study type/ evidence level** | **Study details/limitations** | **Patients’ characteristics** | **Interventions** |
| **Study type:** Systematic review and meta-analysis of randomized controlled trials  **Evidence level:** High | ***Countries:*** Not applicable  ***Centers:*** Not applicable  ***Setting:*** Patients with acute pancreatitis requiring nutritional support  ***Funding sources*:** None  ***Dropout rates:*** Not applicable  ***Limitations*:** Imprecision (The majority of studies were of small sample size and with wide confidence intervals only one study) | Eight trials with a total of 348 participants were included | Enteral nutrition (EN) versus Parenteral nutrition (PN) |
| **Outcome Measurement/results** | Mortality, morbidity and length of hospital stay | Comparing EN to TPN for acute pancreatitis, the relative risk (RR) for death was 0.50 (95% CI 0.28 to 0.91), for multiple organ failure (MOF) was 0.55 (95% CI 0.37 to 0.81), for systemic infection was 0.39 (95% CI 0.23 to 0.65), for operative interventions was 0.44 (95% CI 0.29 to 0.67), for local septic complications was 0.74 (95% CI 0.40 to 1.35), and for other local complications was 0.70 (95% CI 0.43 to 1.13). Mean length of hospital stay was reduced by 2.37 days in EN vs TPN groups (95% CI -7.18 to 2.44). Furthermore, a subgroup analysis for EN vs TPN in patients with severe acute pancreatitis showed a RR for death of 0.18 (95% CI 0.06 to 0.58) and a RR for MOF of 0.46 (95% CI 0.16 to 1.29) | |
| **Conclusions** | In patients with acute pancreatitis, enteral nutrition signiﬁcantly reduces mortality, multiple organ failure, systemic infections, and the need for operative interventions compared to those who receive TPN. In addition, there is a trend towards a reduction in the length of hospital stay. These data suggest that EN should be considered the standard of care for patients with acute pancreatitis requiring nutritional support | | |

| **Paper:** Timing of enteral nutrition in acute pancreatitis: Meta-analysis of individuals using a single-arm of randomised trials  Pancreatology 2014  Bakker OJ, *et al.* | | | |
| --- | --- | --- | --- |
| **Study type/ evidence level** | **Study details/limitations** | **Patients’ characteristics** | **Interventions** |
| **Study type:** Meta-analysis of single-arm randomized trials  **Evidence level:** High | ***Countries:*** Not applicable  ***Centers:*** Not applicable  ***Setting:*** Patients with acute pancreatitis requiring nutritional support  ***Funding sources*:** The Netherlands Organization for Health Research and Development (ZonMw, grant number 17099.2902)  ***Dropout rates:*** Not applicable  ***Limitations*:** - | 165 patients from 8 randomized trials | 100 patients with enteral nutrition (EN) within 24 h and 65 patients with EN after 24 h of admission |
| **Outcome Measurement/results** | Composite endpoint: infected pancreatic necrosis, organ failure, or mortality | In the multivariable model, EN started within 24 h of admission compared to EN started after 24 h of admission, reduced the composite endpoint from 45% to 19% (adjusted odds ratio [OR] of 0.44; 95% conﬁdence interval [CI] 0.20-0.96). Within the composite endpoint, organ failure was reduced from 42% to 16% (adjusted OR 0.42; 95% CI 0.19-0.94) | |
| **Conclusions** | Starting EN within 24 h after hospital admission, compared with after 24 h, was associated with a reduction in complications | | |

| **Paper:** Efﬁcacy comparisons of enteral nutrition and parenteral nutrition in patients with severe acute pancreatitis: a meta-analysis from randomized controlled trials  Bioscience reports 2018  Wu P, *et al.* | | | |
| --- | --- | --- | --- |
| **Study type/ evidence level** | **Study details/limitations** | **Patients’ characteristics** | **Interventions** |
| **Study type:** Meta-analysis of randomized controlled trials  **Evidence level:** High | ***Countries:*** Not applicable  ***Centers:*** Not applicable  ***Setting:*** Patients with severe acute pancreatitis  ***Funding sources*:** None  ***Dropout rates:*** Not applicable  ***Limitations*:** Risk of bias during blinding and allocation | Eleven studies included a total of 562 patients with severe acute pancreatitis | 281 patients received enteral nutrition (EN) and 281 patients received parenteral nutrition (PN) |
| **Outcome Measurement/results** | Mortality, infections, complications, multiple organ failure, length of hospital stay | EN decreased the mortality rate (relative risk [RR] 0.43, 95% confidence interval [CI]: 0.23–0.78, P= 0.006) and lowered the risk of infection and complications (RR 0.53, 95% CI: 0.39–0.71, P= 0.000) more so than does PN. The EN group had a similar risk of multiple organ failure (MOF) compared with the PN group (RR 0.63, 95% CI: 0.39–1.02, P= 0.059). The use of EN was also found to significantly reduce mean hospitalization time (mean difference −2.93, 95% CI: −4.52–1.34, P= 0.000) | |
| **Conclusions** | Compared to PN, EN significantly reduces the risk of mortality, infection, and complications for patients with SAP. EN support also decreases the rate of MOF and surgical intervention | | |

| **Paper:** Meta-Analysis of Enteral Nutrition versus Total Parenteral Nutrition in Patients with Severe Acute Pancreatitis  Annals of nutrition and metabolism 2008  Cao Y, *et al.* | | | |
| --- | --- | --- | --- |
| **Study type/ evidence level** | **Study details/limitations** | **Patients’ characteristics** | **Interventions** |
| **Study type:** Systematic review and meta-analysis  **Evidence level:** High | ***Countries:*** Not applicable  ***Centers:*** Not applicable  ***Setting:*** Patients with severe acute pancreatitis  ***Funding sources*:** Not reported  ***Dropout rates:*** Not applicable  ***Limitations*:** Limited number of patients were included in the trials; not all trials were blinded due to the nature of the interventions | 6 randomized controlled trials (RCTs) were identified and included in the meta-analysis, with 224 participants with severe acute pancreatitis | Enteral nutrition *versus* total parenteral nutrition |
| **Outcome Measurement/results** | Infections, artificial nutrition-related complications, pancreatitis-related complications, non-pancreatitis-related complications, organ failure, and mortality | Compared with total parenteral nutrition, enteral nutrition was associated with a significantly lower risk of infections [odds ratio (OR) 0.236; 95% confidence interval (95% CI) 0.120–0.464, p< 0.001], pancreatitis-related complications (0.456; 0.234–0.888, p= 0.021), organ failure (0.334; 0.167–0.670, p= 0.002), multiple organ dysfunction syndrome (0.306; 0.128–0.736, p= 0.008), and mortality (0.251; 0.095–0.666, p= 0.005). There were no significant differences in artificial nutrition-related complications (0.642; 0.354–1.162, p= 0.143), and non-pancreatitis-related complications (0.716; 0.325–1.576, p= 0.406) between the two groups | |
| **Conclusions** | Enteral nutrition appears safer than total parenteral nutrition in nutrition support of patients with severe acute pancreatitis | | |

| **Paper:** Enteral Nutrition within 48 Hours of Admission Improves Clinical Outcomes of Acute Pancreatitis by Reducing Complications: A Meta-Analysis  PLOS ONE 2013  Li JY, *et al.* | | | |
| --- | --- | --- | --- |
| **Study type/ evidence level** | **Study details/limitations** | **Patients’ characteristics** | **Interventions** |
| **Study type:** Systematic review and meta-analysis  **Evidence level:** High | ***Countries:*** Not applicable  ***Centers:*** Not applicable  ***Setting:*** Patients with acute pancreatitis  ***Funding sources*:** Supported by National Natural Science Foundation of China (No. 81270442 and No. 81000152) and Youthful Teacher Foster Plan of Sun Yat-Sen University  ***Dropout rates:*** Not applicable  ***Limitations*:** Not all the articles included were RCTs | Eleven studies containing 775 patients with acute pancreatitis | Enteral nutrition (EN) initiated within 48 hours of admission versus total parenteral nutrition (TPN) or EN outside 48 hours |
| **Outcome Measurement/results** | Number of overall infections, catheter-related septic complications, pancreatic infection, hyperglycemia, pulmonary complication, organ failure, death, length of hospitalization | Early enteral nutrition was associated with significant reductions in overall infections (OR 0.38; 95%CI 0.21–0.68, P< 0.05), in catheter-related septic complications (OR 0.26; 95%CI 0.11–0.58, P< 0.05), in pancreatic infection (OR 0.49; 95%CI 0.31–0.78, P< 0.05), in hyperglycemia (OR 0.24; 95%CI 0.11–0.52, P< 0.05), in the length of hospitalization (mean difference 22.18; 95%CI 23.482(20.87); P< 0.05), and in mortality (OR 0.31; 95%CI 0.14–0.71, P< 0.05), but no difference was found in pulmonary complications (P> 0.05). The stratified analysis based on the severity of disease revealed that, even in predicted severe or severe acute pancreatitis patients, early enteral nutrition still showed a protective power against all the infection complications as a whole, catheter-related septic complications, pancreatic infection complications, and organ failure that was only reported in the severe attack of the disease (all P< 0.05). | |
| **Conclusions** | Enteral nutrition within 48 hours of admission improves the clinical outcomes in acute pancreatitis as well as in predicted severe or severe acute pancreatitis by reducing complications | | |

| **Paper:** Meta-analysis of parenteral nutrition versus enteral nutrition in patients with acute pancreatitis  BMJ 2004  Marik PE, et al. | | | |
| --- | --- | --- | --- |
| **Study type/ evidence level** | **Study details/limitations** | **Patients’ characteristics** | **Interventions** |
| **Study type:** Meta-analysis of Randomized Controlled Trials  **Evidence level:** High | ***Countries:*** Not applicable  ***Centers:*** Not applicable  ***Setting:*** Patients with acute pancreatitis  ***Funding sources*:** None  ***Dropout rates:*** Not applicable  ***Limitations*:** Relatively poor quality of the included studies | 263 patients with acute pancreatitis | Randomized controlled studies that compared enteral nutrition with parenteral nutrition in patients with acute pancreatitis |
| **Outcome Measurement/results** | Infections, complications other than infections, operative interventions, length of hospital stay, and mortality | Enteral nutrition was associated with a significantly lower incidence of infections (relative risk 0.45; 95% confidence interval 0.26 to 0.78, P= 0.004), reduced surgical interventions to control pancreatitis (0.48, 0.22 to 1.0, P= 0.05), and a reduced length of hospital stay (mean reduction  2.9 days, 1.6 days to 4.3 days, P< 0.001). There were no significant differences in mortality (relative risk 0.66, 0.32 to 1.37, P= 0.3) or non-infectious complications (0.61, 0.31 to 1.22, P= 0.16) between the two groups of patients | |
| **Conclusions** | Enteral nutrition should be the preferred route of nutritional support in patients with acute pancreatitis | | |

| **Paper:** Early enteral nutrition versus delayed enteral nutrition in acute pancreatitis. A PRISMA-compliant systematic review and meta-analysis  Medicine 2017  Feng P, *et al.* | | | |
| --- | --- | --- | --- |
| **Study type/ evidence level** | **Study details/limitations** | **Patients’ characteristics** | **Interventions** |
| **Study type:** Systematic review and meta-analysis  **Evidence level:** Moderate | ***Countries:*** Not applicable  ***Centers:*** Not applicable  ***Setting:*** Patients with acute pancreatitis  ***Funding sources*:** Not reported  ***Dropout rates:*** Not applicable  ***Limitations*:** Non-randomized studies were included (a sensitivity analysis was performed) | 6 articles, enrolling 1007 patients, were included in this meta-analysis, comprising 2 retrospective studies and 4 RCTs | Early enteral nutrition (EEN) within 48hours versus delayed enteral nutrition (DEN) beyond 48hours |
| **Outcome Measurement/results** | Complications and mortality | For complications, the pooled analysis showed that EEN was related to a reduced risk of multiple organ failure (RR=0.67, 95% CI 0.46–0.99, P= 0.04), but not for necrotizing pancreatitis (RR=0.95, 95% CI 0.81–1.12, P= 0.57). There was a tendency for decreased systemic inﬂammatory response syndrome in the EEN group, but the trend was not signiﬁcant (RR=0.85, 95% CI 0.711.02, P= 0.09). For mortality, no signiﬁcant difference was found between the EEN and DEN groups (RR=0.78, 95% CI 0.27–2.24, P= 0.64) | |
| **Conclusions** | Early enteral nutrition within 48hours is superior to DEN beyond 48hours for patients with acute pancreatitis | | |

| **Paper:** Enteral nutrition provided within 48 hours after admission in severe acute pancreatitis. A systematic review and meta-analysis  Medicine 2018  Song J, *et al.* | | | |
| --- | --- | --- | --- |
| **Study type/ evidence level** | **Study details/limitations** | **Patients’ characteristics** | **Interventions** |
| **Study type:** Systematic review and meta-analysis of randomized controlled trials  **Evidence level:** High | ***Countries:*** Not applicable  ***Centers:*** Not applicable  ***Setting:*** Patients with severe acute pancreatitis  ***Funding sources*:** Supported by grants from the National Science Foundation of China  ***Dropout rates:*** Not applicable  ***Limitations*:** Some included RCTs were small in size and single center. The blinding was not addressed in all included RCTs, different feeding routes were used, Different criteria for the definition of severe acute pancreatitis (SAP) or predicted SAP (pSAP) | 1051 patients with severe acute pancreatitis | Randomized controlled trials of early enteral nutrition (starting within 48hours after admission) versus late enteral nutrition or total parental nutrition in severe acute pancreatitis or predicted severe acute pancreatitis |
| **Outcome Measurement/results** | Mortality, multiple organ failure, operative intervention, systemic infection, local septic complications, gastrointestinal symptoms, systemic inflammatory response syndrome, other local complications | Comparing early enteral nutrition to late enteral nutrition or total parental nutrition in SAP or pSAP, the pooled risk ratios were 0.53 (95% conﬁdence interval [CI] 0.35–0.81, P= 0.003) for mortality, 0.58 (95% CI 0.43–0.77, P= 0.0002) for multiple organ failure (MOF), 0.50 (95% CI 0.33–0.75, P= 0.0008) for operative intervention, 0.75 (95% CI 0.61–0.93, P= 0.009) for systemic infection, 0.42 (95% CI 0.26–0.69, P= 0.0005) for local septic complications, 0.84 (95% CI 0.74–0.96, P= 0.01) for gastrointestinal symptoms. 0.87 (95% CI 0.74–1.02, P= 0.08) for systemic inﬂammatory response syndrome (SIRS), and 1.24 (95% CI 0.66–2.31, P= 0.50) for other local complications | |
| **Conclusions** | Enteral nutrition within 48hours after admission is efﬁcient and safe for the patients with SAP or pSAP | | |

| **Paper:** Inﬂuence of enteral versus parenteral nutrition on blood glucose control in acute pancreatitis: A systematic review  Clinical nutrition 2007  Petrov MS, *et al.* | | | |
| --- | --- | --- | --- |
| **Study type/ evidence level** | **Study details/limitations** | **Patients’ characteristics** | **Interventions** |
| **Study type:** Systematic review and meta-analysis of randomized controlled trials  **Evidence level:** Moderate | ***Countries:*** Not applicable  ***Centers:*** Not applicable  ***Setting:*** Patients with acute pancreatitis  ***Funding sources*:** Not reported  ***Dropout rates:*** Not applicable  ***Limitations*:** Two studies were of relatively poor quality | 264 non-diabetic patients with acute pancreatitis | Enteral versus parenteral nutrition in acute pancreatitis |
| **Outcome Measurement/results** | Risk of hyperglycemia and insulin requirement | Intake of nutrients did not differ among enterally and parenterally fed patients in 5 of 6 randomized controlled trials. Enteral nutrition reduced the risk of hyperglycemia (relative risk 0.53; 95% conﬁdence interval 0.29–0.98; p< 0.04) and insulin requirement (relative risk 0.41; 95% conﬁdence interval 0.24–0.70; p< 0.001) | |
| **Conclusions** | Enteral nutrition, when compared with parenteral nutrition, is associated with better blood glucose control in patients with acute pancreatitis | | |

| **Paper:** Enteral Nutrition and the Risk of Mortality and Infectious Complications in Patients With Severe Acute Pancreatitis. A Meta-analysis of Randomized Trials  Archives of surgery 2008  Petrov MS, *et al.* | | | |
| --- | --- | --- | --- |
| **Study type/ evidence level** | **Study details/limitations** | **Patients’ characteristics** | **Interventions** |
| **Study type:** Meta-analysis of randomized controlled trials  **Evidence level:** High | ***Countries:*** Not applicable  ***Centers:*** Not applicable  ***Setting:*** Patients with severe acute pancreatitis  ***Funding sources*:** Non reported  ***Dropout rates:*** Not applicable  ***Limitations*:** Risk of imprecision for some of the included trials; Inconsistency for some of the analyzed outcomes | 95 patients were randomly allocated to the enteral nutrition (EN) group and 107 to the parenteral nutrition (PN) group | Enteral versus parenteral nutrition in patients with predicted severe acute pancreatitis |
| **Outcome Measurement/results** | Infectious complications, pancreatic infections, mortality, organ failure | Enteral feeding reduced the risk of infectious complications (relative risk, 0.47; 95% confidence interval, 0.280.77; P< 0.001), pancreatic infections (0.48; 0.26-0.91; P= 0.02), and mortality (0.32; 0.11-0.98; P= 0.03). The risk reduction for organ failure was not statistically significant (0.67; 0.30-1.52; P= 0.34) | |
| **Conclusions** | Enteral nutrition results in clinically relevant and statistically significant risk reduction for infectious complications, pancreatic infections, and mortality | | |

| **Paper:** Comparison of complications attributable to enteral and parenteral nutrition in predicted severe acute pancreatitis: a systematic review and meta-analysis  British journal of nutrition 2010  Petrov MS, *et al.* | | | |
| --- | --- | --- | --- |
| **Study type/ evidence level** | **Study details/limitations** | **Patients’ characteristics** | **Interventions** |
| **Study type:** Systematic review and meta-analysis of randomized controlled trials  **Evidence level:** Moderate | ***Countries:*** Not applicable  ***Centers:*** Not applicable  ***Setting:*** Patients with predicted severe acute pancreatitis  ***Funding sources*:** Not reported  ***Dropout rates:*** Not applicable  ***Limitations*:** Different adherence to nutrition protocols, different definitions of diarrhea | Ninety-two (53 %) patients with predicted severe pancreatitis receiving parenteral nutrition (PN), and eighty-two (47%) patients receiving enteral nutrition (EN) | Parenteral nutrition versus enteral nutrition |
| **Outcome Measurement/results** | Complications related to the use of nutrition in patients with predicted severe acute pancreatitis | Diarrhea occurred in six of ninety-two (7%) patients receiving PN and twenty-four of eighty-two (29%) patients receiving EN (OR 0.20; 95% CI 0.09, 0.43; P< 0.001). Hyperglycaemia developed in twenty-one of ninety-two (23%) patients receiving PN and nine of eighty-two (11%) receiving EN (OR 2.59; 95% CI 1.13, 5.94; P= 0.03). PN, when compared with EN, reduced the odds of abdominal bloating by 64% (P= 0.31) per protocol, and by 63% (P= 0.32) by intention to treat, although these were not statistically signiﬁcant | |
| **Conclusions** | Enteral nutrition should be the treatment of choice in acute pancreatitis | | |

| **Paper:** Meta-Analysis of Early Enteral Nutrition Provided Within 24 Hours of Admission on Clinical Outcomes in Acute Pancreatitis  Journal of Parenteral and Enteral Nutrition 2018  Qi D, *et al.* | | | |
| --- | --- | --- | --- |
| **Study type/ evidence level** | **Study details/limitations** | **Patients’ characteristics** | **Interventions** |
| **Study type:** Systematic review and meta-analysis of randomized controlled trials  **Evidence level:** Low | ***Countries:*** Not applicable  ***Centers:*** Not applicable  ***Setting:*** Patients with predicted severe or severe acute pancreatitis  ***Funding sources*:** Not reported  ***Dropout rates:*** Not applicable  ***Limitations*:** 6 of the included trials presented a risk of imprecision and were of poor quality, with high heterogeneity | 727 patients with predicted severe or severe acute pancreatitis | Early enteral nutrition (EN) within 24 hours versus late enteral nutrition >24 hours |
| **Outcome Measurement/results** | Mortality, multiple organ failure, infectious complications, adverse events, pancreatic-related infections | Comparing early EN to late EN or total parental nutrition in AP, the odds ratios (OR) were 0.56 (95% CI 0.23–1.34) for the risk of mortality, 0.40 (95% CI 0.20–0.79) for multiple organ failure, 0.57 (95% CI 0.23–1.42) for infectious complications, 0.45 (95% CI 0.17–1.21) for adverse events, and 0.83 (95% CI 0.59–1.18) for pancreatic-related infections. Subgroup analysis for early EN in predicted severe or SAP showed a significant reduction in multiple organ failure (OR 0.30; 95% CI 0.09–0.96) and pancreatic-related infections (OR 0.51, 95% CI 0.29–0.88). Early EN provided no benefits for mild to moderate AP | |
| **Conclusions** | Early EN within 24 hours of admission provides benefits for predicted severe or severe acute pancreatitis, but not for mild to moderate pancreatitis | | |

| **Paper:** Enteral versus parenteral nutrition in critically ill patients with severe pancreatitis: a meta-analysis  European journal of clinical nutrition 2017  Yao H, *et al.* | | | |
| --- | --- | --- | --- |
| **Study type/ evidence level** | **Study details/limitations** | **Patients’ characteristics** | **Interventions** |
| **Study type:** Systematic review and meta-analysis of randomized controlled trials  **Evidence level:** High | ***Countries:*** Not applicable  ***Centers:*** Not applicable  ***Setting:*** Patients with severe acute pancreatitis  ***Funding sources*:** Not reported  ***Dropout rates:*** Not applicable  ***Limitations*:** Small sample sizes (risk of imprecision), differences in the caloric and protein intake | 348 patients with severe acute pancreatitis | Enteral nutrition (EN) versus parenteral nutrition (PN) |
| **Outcome Measurement/results** | Overall mortality, multiple organ failure | Compared with PN, EN was associated with a signiﬁcant reduction in overall mortality (risk ratio (RR) = 0.36, 95% conﬁdence interval (CI) 0.20–0.65, P= 0.001) and the rate of multiple organ failure (RR= 0.39, 95% CI 0.21–0.73, P= 0.003) | |
| **Conclusions** | Enteral nutrition should be recommended as the preferred route of nutrition for critically ill patients with severe acute pancreatitis | | |

| **Paper:** Enteral nutrition is superior to parenteral nutrition in severe acute pancreatitis: results of a randomized prospective trial  British Journal of Surgery 1997  Kalfarentzos S, *et al.* | | | |
| --- | --- | --- | --- |
| **Study type/ evidence level** | **Study details/limitations** | **Patients’ characteristics** | **Interventions** |
| **Study type:** Randomized Controlled Trial  **Evidence level:** Moderate | ***Countries:*** Greece  ***Centers:*** University of Patras, Patras, Greece  ***Setting:*** Patients with severe acute pancreatitis  ***Funding sources*:** Not reported  ***Dropout rates:*** 5%  ***Limitations*:** Small sample size (imprecision); unclear randomization | 40 patients with acute pancreatitis and three or more criteria according to the Imrie classification, or Acute Physiology And Chronic Health Evaluation (APACHE) II score of 8 or more, C-reactive protein concentration greater than 120 mg/l within 48h of admission, and grade D or E by computed tomography (CT) according to the Balthazar criteria | The first (n = 18) patients received enteral nutrition through a nasoenteric tube with a semi-elemental diet, while the second group (n = 20) received parenteral nutrition through a central venous catheter |
| **Outcome Measurement/results** | Safety (assessed by the clinical course of the disease); laboratory findings; incidence of complications; nitrogen balance; cost of nutritional support | Enteral feeding was well tolerated without adverse effects on the course of the disease. Patients who received enteral feeding experienced fewer total complications (P< 0.05) and were at a lower risk of developing septic complications (P< 0.01) than those receiving parenteral nutrition. The cost of nutritional support was three times higher in patients who received parenteral nutrition | |
| **Conclusions** | Early enteral nutrition should be used preferentially in patients with severe acute pancreatitis | | |

| **Paper:** Effect of Enteral Versus Parenteral Nutrition on Inflammatory Markers in Severe Acute Pancreatitis  Pancreas 2007  Mora J, *et al.* | | | |
| --- | --- | --- | --- |
| **Study type/ evidence level** | **Study details/limitations** | **Patients’ characteristics** | **Interventions** |
| **Study type:** Randomized Controlled Trial  **Evidence level:** Moderate | ***Countries:*** Spain  ***Centers:*** Hospital de la Santa Creu i Sant Pau Barcelona, Spain  ***Setting:*** Patients with severe acute pancreatitis  ***Funding sources*:** Not reported  ***Dropout rates:*** 0%  ***Limitations*:** Small sample size; unclear randomization and allocation methods | 22 patients with severe acute pancreatitis | Patients were randomized to receive either total parenteral nutrition -TPN- (n = 11) or total enteral nutrition -TEN- (n = 11) (Peptisorb; Nutricia SRL, Zoetermeer, the Netherlands) within 72 hours of the onset of the disease |
| **Outcome Measurement/results** | CRP determination, tumor necrosis factor-alpha (TNF alpha), and interleukin 6 (IL-6) concentrations in heparinized plasma samples were obtained on days 0, 5, and 10 after initiation of nutritional support | C reactive protein and IL-6 values decreased in both groups during the ﬁrst 10 days of nutrition, with no signiﬁcant differences between the 2 groups (Mann-Whitney U test). The fall in TNF-alpha values was more evident in TEN patients than in the TPN group, although the effect was not signiﬁcant | |
| **Conclusions** | Early implementation of total enteral nutrition could attenuate, at least with equal efﬁcacy, the inﬂammatory process in patients with severe acute pancreatitis | | |

| **Paper:** Effect of early enteral nutrition (EN) on endotoxin in serum and intestinal permeability in patients with severe acute pancreatitis  European Review for Medical and Pharmacological Sciences 2017  Shen QX, *et al.* | | | |
| --- | --- | --- | --- |
| **Study type/ evidence level** | **Study details/limitations** | **Patients’ characteristics** | **Interventions** |
| **Study type:** Randomized controlled trial  **Evidence level:** Moderate | ***Countries:*** China  ***Centers:*** Zhumadian Central Hospital, Henan, China  ***Setting:*** Patients with severe acute pancreatitis  ***Funding sources*:** Not reported  ***Dropout rates:*** 0%  ***Limitations*:** No info about the sample size calculation | 70 cases of patients with severe acute pancreatitis | Patients selected were randomly divided into two groups, including a group of patients having parenteral nutrition (group PN) and that had enteral nutrition (group EN) |
| **Outcome Measurement/results** | Differences in serum endotoxin level; differences in the lactulose/mannitol ratio of urine before intervention and one and two weeks after the intervention | One and two weeks after the intervention, the serum endotoxin level and the lactulose/mannitol excretion rate of urine of the group PN were significantly higher than the group EN (p< 0.05) | |
| **Conclusions** | EN can better promote the elimination of serum endotoxin and reduce intestinal permeability | | |

| **Paper:** Enteral Nutrition in Severe Acute Pancreatitis  JOP Journal of Pancreas (online) 2009  Doley RP, *et al.* | | | |
| --- | --- | --- | --- |
| **Study type/ evidence level** | **Study details/limitations** | **Patients’ characteristics** | **Interventions** |
| **Study type:** Randomized Controlled Trial  **Evidence level:** Low | ***Countries:*** India  ***Centers***: Postgraduate Institute of Medical Education and Research. Chandigarh, India  ***Setting:*** Patients with severe acute pancreatitis  ***Funding sources*:** Not reported  ***Dropout rates:*** 0%  ***Limitations*:** Unclear randomization and allocation methods | Fifty patients with severe acute pancreatitis | Patients were randomized to receive total enteral nutrition (n=25) or total parenteral nutrition (n=25) |
| **Outcome Measurement/results** | Serum C-reactive protein, transferrin levels, albumin, surgical intervention, infections, duration of hospital stay and mortality | There was a significant decrease in serum C-reactive protein values in both the enteral nutrition group and the total parenteral nutrition group at one week and two weeks (P< 0.001 for both). Serum albumin rose from a prenutritional value of 2.82±0.51 g/dL to 3.34±0.45 g/dL on day 14 of nutritional support in the enteral nutrition group (P= 0.003); in the total parenteral nutrition group, the level rose from 3.10±0.59 g/dL to 3.21±0.30 g/dL (P= 0.638). A significant rise in transferrin value was observed from day 0 to day 14 in the enteral nutrition group (169±30 to 196±36 mg/dL; P< 0.001) whereas, in the total parenteral nutrition group, a less significant difference (191±41 to 201±29 mg/dL; P=0.044) was observed. There was no significant difference in surgical interventions (56.0% versus 60.0%; P=1.000), infective complications (64.0% versus 60.0%; P= 1.000), hospital stay (42 days, 15-108 days, versus 36 days, 20-77 days; median, range; P= 0.755), or mortality (20.0% versus 16.0%; P= 1.000) in enteral nutrition versus total parenteral nutrition, respectively | |
| **Conclusions** | Enteral nutrition and total parenteral nutrition are comparable in the management of severe acute pancreatitis in terms of hospital stay, need for surgical intervention, infections and mortality | | |

| **Paper:** A Randomised Clinical Trial to Assess the Effect of Total Enteral and Total Parenteral Nutritional Support on Metabolic, Inflammatory and Oxidative Markers in Patients with Predicted Severe Acute Pancreatitis (APACHE II ≥6)  Pancreatology 2003  Gupta R, *et al.* | | | |
| --- | --- | --- | --- |
| **Study type/ evidence level** | **Study details/limitations** | **Patients’ characteristics** | **Interventions** |
| **Study type:** Randomized Controlled Trial  **Evidence level:** Low | ***Countries:*** United Kingdom (UK)  ***Centers:*** University Department of Surgery, Southampton General Hospital, and institute of Human Nutrition, University of Southampton, Southampton, UK  ***Setting:*** Patients with predicted severe acute pancreatitis  ***Funding sources*:** Grant from Nutricia  ***Dropout rates:*** 0%  ***Limitations*:** Small sample size (high risk of imprecision) | Patients admitted with predicted severe acute pancreatitis (APACHE II score > 5) | Patients were randomized to total enteral (TEN; n = 8) or total parenteral nutrition (TPN; n = 9) |
| **Outcome Measurement/results** | C-reactive protein, fatigue (visual analog scale), oxidative stress (plasma thiobarbituric acid-reactive substances), plasma glutamine, and anti-endotoxin IgG and IgM antibody concentrations (on admission, days 3 and 7). Clinical progress was monitored using the APACHE II score. Organ failure, complications, and costs | All patients tolerated the feeding regime well, with few nutrition-related complications. Fatigue improved in both groups but more rapidly in the TEN group. Oxidative stress was high on admission and rose by similar amounts in both groups. Plasma glutamine concentrations did not change significantly in either group. In the TPN group, 3 patients developed respiratory failure, and 3 developed non-respiratory single organ failure. There were no such complications in the TEN group. Hospital stay was shorter in the TEN group [7 (4–14) vs. 10 (7–26) days; p= 0.05], as was time to passing flatus and time to opening bowels [1 (0–2) vs. 2 (1–5) days; p= 0.01] The cost of TEN was considerably less than of TPN | |
| **Conclusions** | Immediate institution of nutritional support in the form of TEN is safe in predicted severe acute pancreatitis. It is as safe and as efficacious as TPN and may be beneficial in the clinical course of this disease | | |

| **Paper:** Early Enteral Nutrition Prevent Acute Pancreatitis From Deteriorating in Obese Patients  Journal of clinical gastroenterology 2018  Jin Z, *et al.* | | | |
| --- | --- | --- | --- |
| **Study type/ evidence level** | **Study details/limitations** | **Patients’ characteristics** | **Interventions** |
| **Study type:** Randomized Controlled Trial  **Evidence level:** Moderate | ***Countries:*** China  ***Centers:*** Department of Hepatobiliary Surgery, The Second Afﬁliated Hospital and Yuying Children’s Hospital of Wenzhou Medical University, Wenzhou, P.R. China  ***Setting:*** Patients with moderately severe or severe acute pancreatitis  ***Funding sources*:** Supported by Natural Science Foundation of Zhejiang province (no. LQ16H080002), Grant of National Natural Science Foundation (no. 81600167), and Wenzhou City Science and Technology Projects (no. 2015Y0069)  ***Dropout rates:*** 0%  ***Limitations*:** Unclear randomization methods | Patients with moderately severe acute pancreatitis (AP) or severe AP were divided into the visceral fat obesity (VFO) group and the non-VFO group by obesity index VFO | The patients received “delayed” enteral nutrition (started enteral nutrition feeding after the ﬁrst 48 hours after admission to the hospital: group A: patients of non-VFO, n = 108; group B: VFO patients, n = 88) or EEN (in the VFO subgroup, group C: n = 91) |
| **Outcome Measurement/results** | Complications, clinical outcomes, plasma levels of cytokines, and intestine gut barrier index at different timepoints after admission | Early enteral nutrition prevented the VFO patients from developing pancreatic necrotic infection | |
| **Conclusions** | Early enteral is able to prevent AP from deteriorating in obese patients | | |

| **Paper:** Early Enteral Nutrition in Severe Acute Pancreatitis: A Prospective Randomized Controlled Trial Comparing Nasojejunal and Nasogastric Routes  Journal of clinical gastroenterology 2006  Ajay K, *et al.* | | | |
| --- | --- | --- | --- |
| **Study type/ evidence level** | **Study details/limitations** | **Patients’ characteristics** | **Interventions** |
| **Study type:** Randomized Controlled Trial  **Evidence level:** Moderate | ***Countries:*** India  ***Centers:*** Department of Gastroenterology and Human Nutrition, All India Institute of Medical Sciences, New Delhi, India  ***Setting:*** Patients with severe acute pancreatitis  ***Funding sources*:** Not reported  ***Dropout rates:*** 0%  ***Limitations*:** Small sample size (risk of imprecision) | 31 patients with severe acute pancreatitis | Patients with SAP were randomized to feeding by either nasogastric -NG- (15 patients) or nasojejunal -NJ- (16 patients) tube. A semi-elemental formula was used through an enteral tube in both groups |
| **Outcome Measurement/results** | Recurrence of pain and tolerance of feeding | Recurrence of pain occurred in only 1 patient each in the 2 groups. Diarrhea occurred in 3 and 4 patients in the NJ and NG groups, respectively. There were 4 deaths in the NJ group and 5 in the NG group. Two patients in the NJ group and 1 in the NG group underwent surgery. There was no diﬀerence in the outcome measures (ie, discharge, surgery, and death) | |
| **Conclusions** | Enteral nutrition at a slow infusion is well tolerated by both NJ and NG routes in patients with severe acute pancreatitis. Neither NJ nor NG feeding leads to recurrence or worsening of pain | | |

| **Paper:** A Randomized Controlled Trial of Enteral versus Parenteral Feeding in Patients with Predicted Severe Acute Pancreatitis Shows a Significant Reduction in Mortality and in Infected Pancreatic Complications with Total Enteral Nutrition  Digestive surgery 2006  Petrov MS, *et al.* | | | |
| --- | --- | --- | --- |
| **Study type/ evidence level** | **Study details/limitations** | **Patients’ characteristics** | **Interventions** |
| **Study type:** Randomized controlled trial  **Evidence level:** High | ***Countries:*** Russia  ***Centers:*** Department of Surgery, Nizhny Novgorod State Medical Academy, Nizhny Novgorod, Russia  ***Setting:*** Patients with predicted severe acute pancreatitis  ***Funding sources*:** Not reported  ***Dropout rates:*** 1.4%  ***Limitations*:** Small sample size (risk of imprecision) | 70 patients with objectively graded severe acute pancreatitis | Patients were randomly allocated to receive either total enteral nutrition (TEN) or total parenteral nutrition (TPN) within 72 h of the onset of symptoms. |
| **Outcome Measurement/results** | Infectious complications (infected pancreatic necrosis, pancreatic abscess), multiple organ failure, mortality | The incidence of pancreatic infectious complications (infected pancreatic necrosis, pancreatic abscess) was significantly lower in the enterally fed group (7 vs. 16, p= 0.02). In the TEN group, 7 patients developed multiple organ failure, whereas 17 parenterally fed patients developed multiple organ failure (p= 0.02). Overall mortality was 20%, with two deaths in the TEN group and twelve in the TPN group (p< 0.01) | |
| **Conclusions** | Early TEN could be used as prophylactic therapy for infected pancreatic necrosis since it significantly decreased the incidence of pancreatic infectious complications as well as the frequency of multiple organ failure and mortality | | |

| **Paper:** Randomized controlled trial of the effect of early enteral nutrition on markers of the inflammatory response in predicted severe acute pancreatitis  British journal of surgery 2000  Powell JJ, *et al.* | | | |
| --- | --- | --- | --- |
| **Study type/ evidence level** | **Study details/limitations** | **Patients’ characteristics** | **Interventions** |
| **Study type:** Randomized controlled trial  **Evidence level:** Moderate | ***Countries:*** Scotland, United Kingdom  ***Centers***: University Department of Surgical and Clinical Sciences and *Department of Radiology, Royal Infirmary of Edinburgh, Edinburgh, UK  ***Setting:*** Patients with predicted severe acute pancreatitis  ***Funding sources*:** Supported by a grant awarded by the Scottish Hospitals Endowment Research Trust, and from the Royal Infirmary of Edinburgh NHS Trust  ***Dropout rates:*** 25.4%  ***Limitations*:** High dropout rates, unclear randomization and allocation methods | 27 patients with prognostically severe acute pancreatitis | Patients with prognostically severe acute pancreatitis within 72 h of disease onset were randomized to receive either enteral nutrition or conventional therapy consisting of a nil-by-mouth regimen |
| **Outcome Measurement/results** | serum concentrations of IL-6, sTNFRI, CRP,  intestinal permeability | There were no significant complications of enteral nutrition. The introduction of enteral nutrition did not affect the serum concentrations of IL-6 (P= 0.28), sTNFRI (P= 0.53), or CRP (P= 0.62) over the first 4 days of the study. Although there were no significant differences in intestinal permeability between the two patient groups at admission (P= 0.13), by day 4, abnormal intestinal permeability occurred more frequently in patients receiving enteral nutrition (P= 0.03) | |
| **Conclusions** | Early enteral nutrition did not ameliorate the inflammatory response in patients with prognostically severe acute pancreatitis | | |

| **Paper:** Evaluation of Early Enteral Feeding Through Nasogastric and Nasojejunal Tube in Severe Acute Pancreatitis. A Noninferiority Randomized Controlled Trial  Pancreas, 2012  Singh N, *et al.* | | | |
| --- | --- | --- | --- |
| **Study type/ evidence level** | **Study details/limitations** | **Patients’ characteristics** | **Interventions** |
| **Study type:** Randomized controlled trial  **Evidence level:** High | ***Countries:*** *India*  ***Centers:*** Departments of Gastroenterology and Human Nutrition, and Biostatistics, All India Institute of Medical Sciences, New Delhi, India  ***Setting:*** Patients with severe acute pancreatitis  ***Funding sources*:** Indian Council of Medical Research, New Delhi  ***Dropout rates:*** 2.5%  ***Limitations*:** - | 78 patients with severe acute pancreatitis (SAP) | Patients with SAP were fed via nasogastric (NG) (candidate) or nasojejunal (NJ) (comparative) route |
| **Outcome Measurement/results** | The primary outcome was the occurrence of any infectious complication in blood, pancreatic tissue, bile, or tracheal aspirate. Secondary end points were pain in refeeding, duration of hospital stay, intestinal permeability assessed by lactulose/mannitol excretion, and endotoxemia assessed by endotoxin core antibody types immunoglobulin G and M | The presence of any infectious complication in the NG and NJ groups was 23.1% and 35.9% (signiﬁcantly different), respectively. The effect size of the difference of infectious complications was -12.8 (95% conﬁdence interval, -29.6 to 4.0). The upper limit of the 95% conﬁdence interval was 4.0 and was within the 5% limit set for noninferiority. The value of 8.0 for the number needed to treat implies that 8 patients should be treated with NG compared with the NJ group to prevent 1 patient from any of the infectious complications | |
| **Conclusions** | Early enteral feeding through NG was not inferior to NJ in patients with SAP | | |

| **Paper:** Early Enteral Nutrition Prevents Intra-abdominal Hypertension and Reduces the Severity of Severe Acute Pancreatitis Compared with Delayed Enteral Nutrition: A Prospective Pilot Study  World journal of surgery 2013  Sun JK, *et al.* | | | |
| --- | --- | --- | --- |
| **Study type/ evidence level** | **Study details/limitations** | **Patients’ characteristics** | **Interventions** |
| **Study type:** Randomized clinical trial (pilot)  **Evidence level:** Moderate | ***Countries:*** China  ***Centers:*** Department of General Surgery, Jinling Hospital, Nanjing University School of Medicine, Nanjing, Jiangsu Province, China  ***Setting:*** Patients with severe acute pancreatitis  ***Funding sources*:** Grants from the Key Project of the Eleventh Five-Year Plan Foundation of People’s Liberation Army  ***Dropout rates:*** 0%  ***Limitations*:** Pilot study, small sample size (risk of imprecision), single-center design | 60 patients with severe acute pancreatitis | Enteral nutrition (EN) was started within 48 h after admission in the EEN group and from the 8th day in the delayed enteral nutrition (DEN) group |
| **Outcome Measurement/results** | Intra-abdominal pressure (IAP), intra-abdominal hypertension (IAH), caloric intake and feeding intolerance (FI), severity markers, and clinical outcome variables | No difference about IAP was found. The IAH incidence of the EEN group was signiﬁcantly lower than that of the DEN group from the 9th day (8/30 versus 18/30; P= 0.009) after admission. The FI incidence of the EEN group was higher than that of the DEN group during the initial 3 days of feeding (25/30 versus 12/30; P= 0.001; 22/30 versus 9/30; P= 0.001; 15/30 versus 4/30; P= 0.002). Patients with an IAP< 15 mmHg had lower FI incidence than those with an IAP >15 mmHg on the 1st day (20/22 versus 17/38; P< 0.001), the 3rd day (11/13 versus 8/47; P< 0.001), and the 7th day (3/5 versus 3/55; P= 0.005) of feeding. The severity markers and clinical outcome variables of the EEN group were signiﬁcantly improved | |
| **Conclusions** | Early enteral nutrition did not increase IAP. In contrast, it might prevent the development of IAH | | |

| **Paper:** Effect of enteral nutrition and ecoimmunonutrition on bacterial translocation and cytokine production in patients with severe acute pancreatitis  Journal of surgical research 2013  Wang G, *et al.* | | | |
| --- | --- | --- | --- |
| **Study type/ evidence level** | **Study details/limitations** | **Patients’ characteristics** | **Interventions** |
| **Study type:** Randomized controlled trial  **Evidence level:** High | ***Countries:*** China  ***Centers:*** Department of Digestive Internal Medicine, Pingxiang Hospital, Southern Medical University, Pingxiang, Jiangxi, People’s Republic of China  ***Setting:*** Patients with severe acute pancreatitis  ***Funding sources*:** Supported by the National Natural Science Foundation of China and Natural Science Foundation of Science and Technology Hall of Jiangxi Province, China  ***Dropout rates:*** 0%  ***Limitations*:** Risk of imprecision (small sample size) | 183 patients with severe acute pancreatitis | Patients were randomly divided into three groups receiving parenteral nutrition (PN), enteral nutrition (EN), or EN + EIN (ecoimmunonutrition) |
| **Outcome Measurement/results** | Acute Physiology and Chronic Health Evaluation II (APACHE II) scores, complications (systemic inﬂammatory response syndrome, multiorgan failure, and infections), intestinal bacterial strains of stool, and plasma concentrations of endotoxin, tumor necrosis factor-a (TNF-a), and interleukin (IL) 6 and IL-10 | The percentage of pancreatic sepsis, multiple organ dysfunction syndrome, and mortality was signiﬁcantly lower in the EN group and was further lower in the EN + EIN group than that in the PN group. The plasma concentrations of TNF-a and IL-6 and APACHE II scores were signiﬁcantly decreased in the EN group and were further lowered in the EN + EIN group than those in the PN group. The plasma concentration of IL-10 was higher in the EN group and was further increased in the EN + EIN group than that in the PN group | |
| **Conclusions** | Enteral nutrition plays effective roles in the treatment of severe acute pancreatitis by decreasing the expression of endotoxin, TNF-a, and IL-6 and the bacterial translocation, enhancing the expression of IL-10, and the combination of ecoimmunonutrition with enteral nutrition results in more therapeutic beneﬁts than enteral nutrition alone | | |

| **Paper:** Effects of early enteral nutrition on immune function of severe acute pancreatitis patients  World journal of gastroenterology 2013  Sun JK, *et al.* | | | |
| --- | --- | --- | --- |
| **Study type/ evidence level** | **Study details/limitations** | **Patients’ characteristics** | **Interventions** |
| **Study type:** Randomized controlled trial  **Evidence level:** Very low | ***Countries:*** China  ***Centers:*** Department of Intensive Care Unit, Nanjing First Hospital, Nanjing Medical University, Nanjing, Jiangsu Province, China  ***Setting:*** Patients with severe acute pancreatitis  ***Funding sources*:** Grants from the Key Project of the Eleventh Five-Year Plan of People’s Liberation Army  ***Dropout rates:*** 0%  ***Limitations*:** No statistical power calculation, unclear methods of randomization and allocation | 60 patients with severe acute pancreatitis | Patients were randomly allocated to receive early enteral nutrition (EEN) or delayed enteral nutrition (DEN). Enteral nutrition was started within 48 h after admission in the EEN group, whereas from the 8th day in the DEN group |
| **Outcome Measurement/results** | Immunologic parameters and C-reactive protein (CRP) levels on days 1, 3, 7 and 14 after admission. Clinical outcome variables  multiple organ dysfunction syndrome, systemic inflammatory response syndrome, and pancreatic infection, as well as the duration of intensive care unit stay | The CD4+ T-lymphocyte percentage, CD4+/ CD8+ ratio, and the CRP levels in EEN group became significantly lower than in DEN group from the 7th day after admission. In contrast, the immunoglobulin G (IgG) levels and human leukocyte antigen-DR expression in EEN group became significantly higher than in the DEN group from the 7th day after admission. No difference in CD8+ T-lymphocyte percentage, IgM, and IgA levels was found between the two groups. The incidences of multiple organ dysfunction syndrome, systemic inflammatory response syndrome, and pancreatic infection, as well as the duration of intensive care unit stay, were significantly lower in EEN group than in DEN group. There was no difference in hospital mortality between the two groups | |
| **Conclusions** | Early enteral nutrition moderates the excessive immune response during the early stage of severe acute pancreatitis without leading to subsequent immunosuppression. Early enteral nutrition can improve the clinical outcome, but not decrease the hospital mortality of severe acute pancreatitis patients | | |

| **Paper:** Total Enteral Nutrition in Prevention of Pancreatic Necrotic Infection in Severe Acute Pancreatitis  Pancreas 2010  Wu XM, *et al.* | | | |
| --- | --- | --- | --- |
| **Study type/ evidence level** | **Study details/limitations** | **Patients’ characteristics** | **Interventions** |
| **Study type:** Randomized controlled trial  **Evidence level:** Moderate | ***Countries:*** China  ***Centers:*** Intensive Care Unit, Shengjing Hospital, China Medical University, Shenyang, Liaoning Province, China  ***Setting:*** Patients with severe acute pancreatitis  ***Funding sources*:** Not reported  ***Dropout rates:*** 0%  ***Limitations*:** Unclear methods of randomization and allocation | 107 patients with acute necrotizing pancreatitis | In the ﬁrst week of hospitalization, patients were randomized to feeding by either total parenteral nutrition, TPN, (54 patients) or total enteral nutrition, TEN, (53 patients) |
| **Outcome Measurement/results** | Organ failure, surgical intervention, pancreatic septic necrosis, mortality | 80% of the patients developed organ failure in the group with total parenteral nutrition, which was higher than in the group with total enteral nutrition (21%). 80% and 22% (P< 0.05) of the patients in the total parenteral nutrition and total enteral nutrition groups, respectively, underwent surgical intervention. The incidence of pancreatic septic necroses in the group with total enteral nutrition (23%) was lower than in the group with total parenteral nutrition (72%, P< 0.05). Mortality in the total parenteral nutrition group (43%) was higher than in the total enteral nutrition group (11%, P< 0.05) | |
| **Conclusions** | Total enteral nutrition is better than total parenteral nutrition in the prevention of pancreatic necrotic infection in severe acute pancreatitis | | |

| **Paper:** Compared with parenteral nutrition, enteral feeding attenuates the acute phase response and improves disease severity in acute pancreatitis  Gut 1998  Windsor ACJ, *et al.* | | | |
| --- | --- | --- | --- |
| **Study type/ evidence level** | **Study details/limitations** | **Patients’ characteristics** | **Interventions** |
| **Study type:** Randomized controlled trial  **Evidence level:** Low | ***Countries:*** United Kingdom  ***Centers:*** Department of surgery and department of radiology, St James’s University Hospital, Leeds, UK  ***Setting:*** Patients with acute pancreatitis  ***Funding sources*:** Not reported  ***Dropout rates:*** 0%  ***Limitations*:** High risk of imprecision, unclear methods of randomization and allocation | 34 patients with acute pancreatitis | Patients were stratiﬁed according to disease severity and randomized to receive either total parenteral nutrition (TPN) or total enteral nutrition (TEN) |
| **Outcome Measurement/results** | Glasgow score, Apache II, computed tomography (CT) scan score, C reactive protein (CRP), serum IgM antiendotoxin antibodies (EndoCAb), and total antioxidant capacity (TAC) were determined on admission in 34 patients with acute pancreatitis. SIRS, sepsis, organ failure, intensive care unit stay. | SIRS, sepsis, organ failure, and intensive therapy unit (ITU) stay, were globally improved in the enterally fed patients. The acute phase response and disease severity scores were signiﬁcantly improved following enteral nutrition (CRP: 156 (117–222) to 84 (50141), p< 0.005; APACHE II scores 8 (6–10) to 6 (4–8), p< 0.0001) without change in the CT scan scores. In parenterally fed patients, these parameters did not change, but there was an increase in EndoCAb antibody levels and a fall in TAC. Enterally fed patients showed no change in the level of EndoCAb antibodies and an increase in TAC | |
| **Conclusions** | TEN moderates the acute phase response, and improves disease severity and clinical outcome despite unchanged pancreatic injury on CT scan. Reduced systemic exposure to endotoxin and reduced oxidant stress also occurred in the TEN group. Enteral feeding modulates the inﬂammatory and sepsis response in acute pancreatitis and is clinically beneﬁcial | | |

**10. Supplemental Files Table 9.** **Supplementary Table Report Template Question 7.**

| **Paper:** Impact of the Timing of Endoscopic Retrograde Cholangiopancreatography for the Treatment of Acute Cholangitis: A Meta-analysis and Systematic Review  Surg Laparosc Endosc Percutan Tech 2022  Lyu T, *et al.* | | | |
| --- | --- | --- | --- |
| **Study type/ evidence level** | **Study details/limitations** | **Patients’ characteristics** | **Interventions** |
| **Study type:** Systematic review and meta-analysis of observational studies  **Evidence level:** Moderate | ***Countries:*** Not applicable  ***Centers:*** Not applicable  ***Setting:*** Patients with acute cholangitis  ***Funding sources*:** Not reported  ***Dropout rates:*** Not applicable  ***Limitations*:** The systematic review included non-randomized studies (selection bias), and the characteristics of the patients in the database studies varied signiﬁcantly. The etiology of acute cholangitis was variable in the included studies | 88.562 patients with acute cholangitis (AC) | Meta-analysis of the outcomes associated with particular timings of ERCP for AC |
| **Outcome Measurement/results** | In-hospital mortality (IHM), 30-day mortality, and length of hospital stay (LHS) | Compared with performing ERCP more than 24 hours after admission, ERCP within 24 hours was associated with lower IHM (P< 0.0004), but no difference in 30-day mortality (P= 0.38) was found between the 2 groups. ERCP performed <48 hours after admission was associated with a lower IHM and 30-day mortality (P< 0.00001 and P = 0.03) than ERCP performed > 48 hours after admission. ERCP performed within 24 or 48 hours was associated with a shorter LHS (P< 0.00001 and P< 0.00001, respectively) | |
| **Conclusions** | ERCP within 48 hours of admission is superior to subsequent ERCP with respect to in-hospital mortality, 30-day mortality, and length of hospital stay, and ERCP performed within 24 hours is associated with lower in-hospital mortality and length of hospital stay | | |

**Research question 7. *Should early (within 48-72 hours) ERCP/ES vs. delayed (> 72 hours) or conservative treatment be used in gallstone-induced acute biliary pancreatitis when cholangitis and/or common bile duct obstruction occur?***

| **Paper:** Urgent Endoscopic Retrograde Cholangiopancreatography (ERCP) vs. Conventional Approach in Acute Biliary Pancreatitis Without Cholangitis: An Updated Systematic Review and Meta-Analysis  Cureus 2022  Shrestha DB, *et al.* | | | |
| --- | --- | --- | --- |
| **Study type/ evidence level** | **Study details/limitations** | **Patients’ characteristics** | **Interventions** |
| **Study type:** Systematic review and meta-analysis of randomized trials and observational studies  **Evidence level:** Low | ***Countries:*** Not applicable  ***Centers:*** Not applicable  ***Setting:*** Patients with acute biliary pancreatitis without cholangitis  ***Funding sources*:** Not reported  ***Dropout rates:*** Not applicable  ***Limitations*:** Unclear assessment of the risk of bias. Risk of imprecision. Clinical heterogeneity in the definition of the treatment and baseline characteristics of the included studies | 595 patients with acute biliary pancreatitis without cholangitis | Early ERCP (within 72 hours of presentation) or conservatively (e.g., no ERCP) |
| **Outcome Measurement/results** | In-hospital mortality (primary outcome) of the study. Local and systemic complications, including new-onset organ failure, pneumonia, pancreatic necrosis and pseudocyst, and ICU admission (secondary outcomes) | No significant reduction in mortality (OR 0.59, 95% CI 0.32 to 1.09; p= 0.09); overall complications (OR 0.56, 95% CI 0.30 to 1.01; p=0.05); new-onset organ failure (OR 1.06, 95% CI 0.65 to 1.75;  p= 0.81); pancreatic necrosis (OR 0.80, 95% CI 0.49 to 1.32; p= 0.38); pancreatic pseudo-cyst (OR 0.44, 95% CI 0.16 to 1.24; p= 0.12); ICU admission (OR 1.64, 95% CI 0.97 to 2.77; p= 0.06); and pneumonia development (OR 0.81, 95% CI 0.40 to 1.65; p= 0.56) by urgent ERCP comparing with conventional approach for acute biliary pancreatitis without cholangitis | |
| **Conclusions** | Early ERCP in acute biliary pancreatitis without cholangitis did not reduce mortality, complications, and other adverse outcomes compared to the conservative treatment | | |

| **Paper:** Timing of Performing Endoscopic Retrograde Cholangiopancreatography and Inpatient Mortality in Acute Cholangitis: A Systematic Review and Meta-Analysis  Clinical and Translational Gastroenterology 2020  Du L, *et al.* | | | |
| --- | --- | --- | --- |
| **Study type/ evidence level** | **Study details/limitations** | **Patients’ characteristics** | **Interventions** |
| **Study type:** Systematic review and meta-analysis of observational studies  **Evidence level:** Low | ***Countries:*** Not applicable  ***Centers:*** Not applicable  ***Setting:*** Patients with acute cholangitis  ***Funding sources*:** Funded by the National Natural Science Foundation of China, Zhejiang Provincial Natural Science Foundation, and Zhejiang Provincial Medical Health Science and Technology Projects  ***Dropout rates:*** Not applicable  ***Limitations*:** Meta-analysis of non-randomized studies, study heterogeneity for acute pancreatitis and immunosuppression, lack of severity stratification, indirectness | 84.063 patients with acute cholangitis | Timing of ERCP (<24, <48, and <72 hours from hospitalization) |
| **Outcome Measurement/results** | In-hospital mortality | The overall pooled in-hospital mortality with acute cholangitis was 1.9% (95% confidence interval [CI] 1.8%–7.6%), which increased to 4.3% (95% CI 1.8%–8.7%) when administrative database studies were excluded. In 9 studies, ERCP performed <24 compared with ≥24 hours decreased in-hospital mortality (odds ratio [OR] 5 0.81, 95% CI 0.73–0.90; I2 50%). In 8 studies, ERCP performed <48 compared with ≥48 hours decreased in-hospital mortality (OR 0.57, 95% CI 0.51–0.63; I2 50%). In 4 studies, ERCP performed <72 compared with ≥72 hours decreased in hospital mortality (OR 0.32, 95% CI 0.15–0.68; I2 50%). Furthermore, hospital stay was reduced in patients receiving ERCP <24 compared with ≥24 hours (mean difference [MD] 3.2 days, 95% CI 2.3–4.1; I2 78%), <48 compared with ≥48 hours (MD 3.6 days, 95% CI 2.1–5.1; I2 98%), and <72 compared with ≥72 hours (MD 4.1 days, 95% CI 0.9–7.3; I2 63%) | |
| **Conclusions** | Earlier ERCP performed in patients with acute cholangitis, even urgently performed <24 hours from presentation, was associated with reduced mortality | | |

| **Paper:** Emergent versus urgent ERCP in acute cholangitis: a systematic review and meta-analysis  Gastrointestinal endoscopy 2020  Iqbal U, *et al.* | | | |
| --- | --- | --- | --- |
| **Study type/ evidence level** | **Study details/limitations** | **Patients’ characteristics** | **Interventions** |
| **Study type:** Systematic review and meta-analysis of observational studies  **Evidence level:** Moderate | ***Countries:*** Not applicable  ***Centers***: Not applicable  ***Setting:*** Patients with acute cholangitis  ***Funding sources*:** Not reported  ***Dropout rates:*** Not applicable  ***Limitations*:** Meta-analysis of non-randomized studies, lack of severity stratification, indirectness | 7534 patients with acute cholangitis | Timing of ERCP on patient outcomes |
| **Outcome Measurement/results** | In-hospital mortality (IHM) (primary outcomes), length of stay (LOS), organ failure, and 30-day mortality | IHM was signiﬁcantly lower in patients who underwent emergent biliary drainage within 48 hours (odds ratio [OR], 0.52; 95% conﬁdence interval [CI], 0.28-0.98). As a sensitivity analysis, we pooled the data from 2 population registry studies of 81.893 patients, which yielded consistent results for the main outcomes. LOS was also signiﬁcantly lower in patients who underwent ERCP within 48 hours with a mean difference of 5.56 days (95% CI, 1.59-9.53). Patients who underwent emergent ERCP also had lower odds of 30-day mortality (OR, 0.39; 95% CI, 0.14-1.08) and organ failure (OR, 0.69; 95% CI, 0.33-1.46) | |
| **Conclusions** | Performing emergent ERCP within 48 hours in patients with acute cholangitis is associated with lower IHM, 30-day mortality, organ failure, and shorter LOS | | |

| **Paper:** Early Endoscopic Retrograde Cholangiopancreatography Versus Conservative Treatment in Patients With Acute Biliary Pancreatitis. Systematic Review and Meta-analysis of Randomized Controlled Trials  Pancreas 2018  Coutinho LMdA, *et al.* | | | |
| --- | --- | --- | --- |
| **Study type/ evidence level** | **Study details/limitations** | **Patients’ characteristics** | **Interventions** |
| **Study type:** Systematic review and meta-analysis of randomized controlled trials  **Evidence level:** Moderate | ***Countries:*** Not applicable  ***Centers:*** Not applicable  ***Setting:*** Patients with acute biliary pancreatitis  ***Funding sources*:** Not reported  ***Dropout rates:*** Not applicable  ***Limitations*:** Heterogeneity in inclusion criteria (presence of cholangitis, only a few studies evaluated the benefits of ERCP separately in moderate acute pancreatitis and severe acute pancreatitis, the criteria used to classify the severity of acute pancreatitis varied across the studies selected) | 1091 patients with acute biliary pancreatitis | A treatment arm involving the use of ERCP within the first 72 hours after admission (intervention arm) and a conservative treatment arm (control arm) |
| **Outcome Measurement/results** | Local adverse events, time to pain relief, time to a reduction in axillary temperature, length of hospital stay, systemic adverse events, development of acute cholangitis, mortality | There was a statistically significant difference between the patients submitted to ERCP in terms of the following: local adverse events (RD, 0.74; 95% confidence interval [CI], 0.55–0.99), time to pain relief and time to a reduction in axillary temperature (RD, −5.01; 95% CI, −6.98 to −3.04, and RD, −1.70; 95 CI%, −2.33 to −1.08, respectively). Patients undergoing ERCP spent less time in hospital (RD, −11.04; 95% CI, −15.15 to −6.93). Cost was lower in the group treated with ERCP. However, no statistical difference between early ERCP and conservative treatment in patients with acute biliary pancreatitis, in terms of the occurrence of systemic adverse events, the development of acute cholangitis, and evolution to death was found | |
| **Conclusions** | Early ERCP decreases local adverse events, shortening the time to pain relief, to a reduction in axillary temperature, hospital stays, and cost in patients with acute biliary pancreatitis | | |

| **Paper:** Meta-Analysis of Early Endoscopic Retrograde Cholangiopancreatography (ERCP) ± Endoscopic Sphincterotomy (ES) Versus Conservative Management for Gallstone Pancreatitis (GSP)  Surg Laparosc Endosc Percutan Tech 2015  Burstow MJ, *et al.* | | | |
| --- | --- | --- | --- |
| **Study type/ evidence level** | **Study details/limitations** | **Patients’ characteristics** | **Interventions** |
| **Study type:** Systematic review and meta-analysis of randomized controlled trials  **Evidence level:** High | ***Countries:*** Not applicable  ***Centers:*** Not applicable  ***Setting:*** Patients with acute biliary pancreatitis  ***Funding sources*:** Not reported  ***Dropout rates:*** Not applicable  ***Limitations*:** Heterogeneity of the included studies, exclusion and inclusion criteria, publication bias | 1314 patients with gallstones pancreatitis | Early intervention (ie, between 24 and 72 h) with ERCP ± ES versus conservative management |
| **Outcome Measurement/results** | Overall mortality, overall complications (pseudocyst formation, organ failure, renal, respiratory, and cardiac, abnormal coagulation, biliary sepsis, development of pancreatic abscess/phlegmon | There was a near signiﬁcant decrease in mortality for ERCP group compared with conservatively managed patients with severe pancreatitis [odds ratio (OR) 0.45; 95% conﬁdence interval (CI), 0.19, 1.09; P= 0.08]. In patients with mild pancreatitis, mortality results were comparable for both groups (OR 0.66; 95% CI, 0.02, 28.75; P= 0.83). Overall complications were signiﬁcantly reduced in the ERCP group in severe pancreatitis patients (OR 0.32; 95% CI, 0.17, 0.61; P= 0.00). In those with mild disease, a strong trend to decreased complications in the ERCP group was seen. However, this was not signiﬁcant (OR 0.67; 95% CI, 0.43, 1.03; P= 0.06) | |
| **Conclusions** | There was a signiﬁcant decrease in complications in patients with severe biliary pancreatitis managed with early ERCP/ES compared with conservative management. No signiﬁcant decrease was observed in mortality, even in severe pancreatitis patients treated with early ERCP/ES | | |

| **Paper:** Early routine endoscopic retrograde cholangiopancreatography strategy versus early conservative management strategy in acute gallstone pancreatitis (Review)  Cochrane Collaboration 2012  Tse F, *et al.* | | | |
| --- | --- | --- | --- |
| **Study type/ evidence level** | **Study details/limitations** | **Patients’ characteristics** | **Interventions** |
| **Study type:** Systematic review and meta-analysis of randomized controlled trials  **Evidence level:** High | ***Countries:*** Not applicable  ***Centers:*** Not applicable  ***Setting:*** Patients with acute gallstone pancreatitis  ***Funding sources*:** McMaster University, Canada, NHS National Institute for Health Research, UK  ***Dropout rates:*** Not applicable  ***Limitations*:** Risk of imprecision | 644 patients with suspected acute gallstone pancreatitis | Early routine ERCP strategy (within 72 hours of admission) versus the early conservative management with or without selective use of ERCP strategy |
| **Outcome Measurement/results** | Mortality, local and systemic complications | In unselected patients with acute gallstone pancreatitis, there were no statistically signiﬁcant differences between the two strategies in mortality (RR 0.74, 95% CI 0.18 to 3.03) and local and systemic complications as deﬁned by the Atlanta Classiﬁcation (RR 0.86, 95% CI 0.52 to 1.43; and RR 0.59, 95% CI 0.31 to 1.11 respectively). Among trials that included patients with cholangitis, the early routine ERCP strategy signiﬁcantly reduced mortality (RR 0.20, 95% CI 0.06 to 0.68), local and systemic complications as deﬁned by the Atlanta Classiﬁcation (RR 0.45, 95% CI 0.20 to 0.99; and RR 0.37, 95% CI 0.18 to 0.78 respectively) and by authors of the primary study (RR 0.50, 95% CI 0.29 to 0.87; and RR 0.41, 95% CI 0.21 to 0.82 respectively). Among trials that included patients with biliary obstruction, the early routine ERCP strategy was associated with a signiﬁcant reduction in local complications as deﬁned by authors of the primary study (RR 0.54, 95% CI 0.32 to 0.91), and a non-signiﬁcant trend towards reduction of local and systemic complications as deﬁned by the Atlanta Classiﬁcation (RR 0.53, 95% CI 0.26 to 1.07; and RR 0.56, 95% CI 0.30 to 1.02 respectively) and systemic complications as deﬁned by authors of the primary study (RR 0.59, 95% CI 0.35 to 1.01). ERCP complications were infrequent | |
| **Conclusions** | In patients with acute gallstone pancreatitis, there is no evidence that early routine ERCP signiﬁcantly affects mortality, and local or systemic complications of pancreatitis, regardless of predicted severity. However, this Cochrane review provides support for current recommendations that early ERCP should be considered in patients with co-existing cholangitis or biliary obstruction | | |

| **Paper:** Early ERCP in Acute Gallstone Pancreatitis without Cholangitis: A Meta-analysis  J Pancreas (Online) 2008  Uy MC, *et al.* | | | |
| --- | --- | --- | --- |
| **Study type/ evidence level** | **Study details/limitations** | **Patients’ characteristics** | **Interventions** |
| **Study type:** Systematic review and meta-analysis of randomized controlled trials  **Evidence level:** Moderate | ***Countries:*** Not applicable  ***Centers:*** Not applicable  ***Setting:*** Patients with acute biliary pancreatitis without cholangitis  ***Funding sources*:** Not reported  ***Dropout rates:*** Not applicable  ***Limitations*:** Risk of imprecision, heterogeneity | 340 patients with acute gallstone pancreatitis without cholangitis | Early ERCP with or without endoscopic sphincterotomy vs. conservative treatment within at most 72 h of admission |
| **Outcome Measurement/results** | Morbidity, mortality | The meta-analysis on morbidity was inconclusive (RR=0.95, 95% CI: 0.74-1.22). The meta-analysis on mortality only showed a trend in favor of conservative management (RR=1.92, 95% CI: 0.86-4.32) for both mild and severe pancreatitis | |
| **Conclusions** | There is a trend towards more mortality from early ERCP with or without sphincterotomy in acute gallstone pancreatitis without cholangitis. Early ERCP should not be carried out unless there is at least a slight suspicion of cholangitis or persistent ampullary obstruction | | |

| **Paper:** Does endoscopic retrograde cholangiopancreatography reduce the risk of local pancreatic complications in acute pancreatitis? A systematic review and meta-analysis  Surgical endoscopy 2008  Petrov MS, *et al.* | | | |
| --- | --- | --- | --- |
| **Study type/ evidence level** | **Study details/limitations** | **Patients’ characteristics** | **Interventions** |
| **Study type:** Systematic review and meta-analysis of randomized controlled trials  **Evidence level:** High | ***Countries:*** *Not applicable*  ***Centers: Not applicable***  ***Setting:*** *Patients with acute biliary pancreatitis*  ***Funding sources*:** Not reported  ***Dropout rates: Not applicable***  ***Limitations*:** Heterogeneity (difference in the deﬁnition of ‘‘early’’ intervention among the trials, which varied from 24 h after the onset of symptoms to 72 h after admission; difference in severity; mixed populations of obstructed and non-obstructed patients) | 717 patients with acute biliary pancreatitis | Early ERCP versus conservative treatment. The intervention group had to receive ERCP with or without ES within 72 h after admission, whereas patients in the control group had to be allocated to conservative treatment initially |
| **Outcome Measurement/results** | Local pancreatic complications, including infected pancreatic necrosis, pancreatic abscess, and pancreatic pseudocyst according to the Atlanta Classiﬁcation | Five trials involving 717 patients were included in this systematic review. Pooled analysis of all the patients with acute pancreatitis did not demonstrate a statistically signiﬁcant difference between the two treatment strategies (relative risk [RR], 0.94; 95% conﬁdence interval [CI],0.63–1.40; p= 0.62). Similar results were observed after subgroup analysis based on the severity of the disease as follows: mild acute pancreatitis (RR, 0.79; 95% CI, 0.26–2.47; p= 0.69); severe acute pancreatitis (RR, 0.77; 95% CI, 0.30–1.98; p= 0.59) | |
| **Conclusions** | The early use of ERCP did not result in a signiﬁcantly reduced risk of local pancreatic complications for either patients with mild acute pancreatitis or those with severe form of the disease | | |

| **Paper:** Is early endoscopic retrograde cholangiopancreatography useful in the management of acute biliary pancreatitis? A meta-analysis of randomized controlled trials  Digestive and liver disease 2008  Moretti A, *et al.* | | | |
| --- | --- | --- | --- |
| **Study type/ evidence level** | **Study details/limitations** | **Patients’ characteristics** | **Interventions** |
| **Study type:** Systematic review and meta-analysis of randomized controlled trials  **Evidence level:** Moderate | ***Countries:*** Not applicable  ***Centers:*** Not applicable  ***Setting:*** Patients with acute biliary pancreatitis  ***Funding sources*:** Not reported  ***Dropout rates:*** Not applicable  ***Limitations*:** Risk of imprecision, heterogeneity | 702 patients with acute biliary pancreatitis | The intervention group received ERCP with or without ES within 72 h after admission, whereas patients in the control group received conservative treatment initially |
| **Outcome Measurement/results** | Pancreatitis complications, mortality | In predicted severe pancreatitis the pooled rate difference for complications in early endoscopic retrograde cholangiopancreatography was 38.5% (95% CI −53% to −23.9%); p< 0.0001. In predicted mild pancreatitis the pooled rate difference for complications in early endoscopic retrograde cholangiopancreatography was 1.8% (95% CI −5.6% to 9.3%); p= 0.6. No mortality was observed in predicted mild pancreatitis. In predicted severe pancreatitis the pooled rate difference for mortality in the early endoscopic retrograde cholangiopancreatography group was 4.3% (95% CI −16% to 7.5%) | |
| **Conclusions** | Early endoscopic retrograde cholangiopancreatography reduces pancreatits-related complications in patients with predicted severe pancreatitis although mortality rate is not affected | | |

| **Paper:** Early Endoscopic Retrograde Cholangiopancreatography Versus Conservative Management in Acute Biliary Pancreatitis Without Cholangitis. A Meta-Analysis of Randomized Trials  Annals of surgery 2008  Petrov MS, *et al.* | | | |
| --- | --- | --- | --- |
| **Study type/ evidence level** | **Study details/limitations** | **Patients’ characteristics** | **Interventions** |
| **Study type:** Systematic review and meta-analysis of randomized controlled trials  **Evidence level:** High | ***Countries:*** Not applicable  ***Centers:*** Not applicable  ***Setting:*** Acute biliary pancreatitis patients without cholangitis  ***Funding sources*:** Not reported  ***Dropout rates:*** *Not applicable*  ***Limitations*:** The methodological quality of the included trials was relatively low, and heterogeneity in the definition of acute cholangitis | 450 patients with acute biliary pancreatitis (ABP) without acute cholangitis | 230 patients were included in the interventional arm (ERCP within 72 hours and 220 in the control arm. Early ERCP (i.e., within 72 hours after admission) with or without ES. Conservative treatment with selective ERCP with or without ES |
| **Outcome Measurement/results** | Mortality and overall complications | In all patients with ABP (predicted mild and severe), early ERCP was associated with a nonsigniﬁcant reduction in overall complications (RR 0.76; 95% conﬁdence interval (CI) 0.41–1.04; P= 0.38) and a nonsigniﬁcant increase in mortality (RR 1.13; 95% CI 0.23–5.63; P= 0.88). Subgroup analysis based on predicted severity did not affect these outcomes (overall complications: predicted mild: RR 0.86; 95% CI 0.62–1.19; P= 0.36; predicted severe: RR 0.82; 95% CI 0.32–2.10; P= 0.68; mortality: predicted mild: RR 1.90; 95% CI 0.25–14.55; P= 0.53; predicted severe: RR 1.28; 95% CI 0.20–8.06; P= 0.80) | |
| **Conclusions** | Early ERCP in patients with predicted mild and predicted severe acute biliary pancreatitis without acute cholangitis does not lead to a signiﬁcant reduction in the risk of overall complications and mortality | | |

| **Paper:** Endoscopic retrograde cholangiopancreatography in gallstone-associated acute pancreatitis (Review)  Cochrane Collaboration 2009  Ayub K, *et al.* | | | |
| --- | --- | --- | --- |
| **Study type/ evidence level** | **Study details/limitations** | **Patients’ characteristics** | **Interventions** |
| **Study type:** Systematic review and meta-analysis of randomized controlled trials  **Evidence level:** High | ***Countries:*** Not applicable  ***Centers:*** Not applicable  ***Setting:*** Adult patients, 15 years old or greater, presenting with gallstone-associated acute pancreatitis  ***Funding sources*:** None  ***Dropout rates:*** Not applicable  ***Limitations*:** Heterogeneity of trials (confounding due to associated acute cholangitis) | 511 patients with gallstone-associated acute pancreatitis (GAP) | ERCP +/- ES (endoscopic sphincterotomy) versus Conservative management within 72 hours of admission |
| **Outcome Measurement/results** | Mortality, morbidity, length of hospitalization and cost | Early ERCP +/- ES was associated with non-signiﬁcant effect on reduction of mortality in predicted mild (OR = 0.62, 95% CI = 0.27 to 1.41) and severe GAP (OR = 0.62, 95% CI = 0.27 to 1.41). Reduction in complications was non-signiﬁcant in predicted mild (OR = 0.89, 95% CI = 0.53 to 1.49), but signiﬁcant in severe GAP (OR = 0.27, 95% CI = 0.14 to 0.53). There was insufﬁcient evidence to draw any conclusions about hospital stay and cost | |
| **Conclusions** | Odds of having complications are reduced in predicted severe disease by early ERCP +/- ES | | |

| **Paper:** Outcomes of delayed versus early endoscopic intervention for acute biliary pancreatitis with non-severe acute cholangitis  BMC surgery 2022  Lyu Y, *et al.* | | | |
| --- | --- | --- | --- |
| **Study type/ evidence level** | **Study details/limitations** | **Patients’ characteristics** | **Interventions** |
| **Study type:** Retrospective cohort study  **Evidence level:** Low | ***Countries:*** China  ***Centers:*** Department of Hepatobiliary Surgery, Dongyang People’s Hospital, Affiliated Dongyang Hospital of Wenzhou Medical University, Zhejiang, People’s Republic of China  ***Setting:*** Patients with acute biliary pancreatitis with non-severe acute cholangitis  ***Funding sources*:** Supported by the Scientific and Technological research project of JinHua, China  ***Dropout rates:*** Not applicable  ***Limitations*:** Retrospective design (selection bias), small sample size, lack of clear diagnostic criteria for acute cholangitis in biliary pancreatitis | 164 patients with concurrent acute biliary pancreatitis and non-severe acute cholangitis  (the definition and severity of acute cholangitis were determined according to the 2018 Tokyo Guidelines) | The patients were divided into two groups: those who underwent ERCP ≤ 72 h after admission (early ERCP group) and those who underwent ERCP > 72 h after admission (delayed ERCP group) |
| **Outcome Measurement/results** | Technical success rate and ERCP-related complications (primary outcomes), hospital stay and patient cost (secondary outcomes) | The study involved 164 patients (early ERCP, n = 70; delayed ERCP, n = 94) who were treated from 1 December 2 to 2016 to 12 December 2021. The patients’ baseline characteristics were not significantly different between the two groups. The technical success rate of ERCP was similar between the two groups (94.29% vs. 97.87%, p= 0.43). Morbidity was also similar between the two groups (p= 0.83). There was no significant difference in the total hospital stay (p= 0.13). However, the early ERCP group had a longer post-ERCP hospital stay (p< 0.001) | |
| **Conclusions** | Delayed ERCP performed > 72 h after admission has economic and safety outcomes similar to those of early ERCP for patients with concurrent acute biliary pancreatitis and non-severe acute cholangitis | | |

| **Paper:** Timing of endoscopic retrograde cholangiopancreatography in the treatment of acute cholangitis of different severity  World journal of gastroenterology 2022  Huang YC, *et al.* | | | |
| --- | --- | --- | --- |
| **Study type/ evidence level** | **Study details/limitations** | **Patients’ characteristics** | **Interventions** |
| **Study type:** Retrospective cohort study  **Evidence level:** Moderate | ***Countries:*** Taiwan  ***Centers:*** Department of Gastroenterology and Hepatology, Chang Gung Memorial Hospital, Taoyuan, Taiwan  ***Setting:*** Patients with acute cholangitis with different severities  ***Funding sources*:** Not reported  ***Dropout rates:*** Not applicable  ***Limitations*:** Retrospective design (risk of selection bias) | According to the 2018 Tokyo guidelines, 683 patients who met the diagnostic criteria for acute cholangitis were retrospectively identified | The results were compared between patients receiving ERCP ≤ 24 h and > 24 h and then between patients receiving ERCP ≤ 48 h and > 48 h. Subgroup analyses were performed in patients with grade I, II or III acute cholangitis |
| **Outcome Measurement/results** | The primary outcome was 30-d mortality. Secondary outcomes were intensive care unit (ICU) admission rate, length of hospital stay (LOHS) and 30-d readmission rate | Taking 24 h as the critical value, compared with ERCP > 24 h, malignant biliary obstruction as a cause of AC was significantly less common in the ERCP ≤ 24 h group (5.2% vs 11.5%). The proportion of cardiovascular dysfunction (11.2% vs 2.6%), respiratory dysfunction (14.2% vs 5.3%), and ICU admission (11.2% vs 4%) in the ERCP ≤ 24 h group was significantly higher, while the LOHS was significantly shorter (median, 6 d vs 7 d). Stratified by the severity of AC, higher ICU admission was only observed in grade III AC and shorter LOHS was only observed in grade I and II AC. There were no significant differences in 30-d mortality between groups, either in the overall population or in patients with grade I, II or III AC. With 48 h as the critical value, compared with ERCP > 48 h, the proportion of choledocholithiasis as the cause of AC was significantly higher in the ERCP ≤ 48 h group (81.5% vs 68.3%). The ERCP ≤ 48 h group had significantly lower 30-d mortality (0 vs 1.9%) and shorter LOHS (6 d vs 8 d). Stratified by AC severity, lower 30-d mortality (0 vs 6.1%) and higher ICU admission rates (22.2% vs 10.2%) were only observed in grade III AC, and shorter LOHS was only observed in grade I and II AC. In the multivariate analysis, cardiovascular dysfunction and time to ERCP were two independent factors associated with 30-d mortality | |
| **Conclusions** | ERCP ≤ 48 h conferred a survival benefit in patients with grade III acute cholangitis | | |

| **Paper:** Optimal timing of biliary drainage based on the severity of acute cholangitis: A single-center retrospective cohort study  World journal of gastroenterology 2022  Lu ZQ, *et al.* | | | |
| --- | --- | --- | --- |
| **Study type/ evidence level** | **Study details/limitations** | **Patients’ characteristics** | **Interventions** |
| **Study type:** Retrospective cohort study  **Evidence level:** Moderate | ***Countries:*** China  ***Centers:*** Department of Emergency Medicine, Beijing Friendship Hospital, Capital Medical University, Beijing, China  ***Setting:*** Acute cholangitis patients with each disease severity grade and organ dysfunction  ***Funding sources*:** Not reported  ***Dropout rates:*** Not applicable  ***Limitations*:** Retrospective design (selection bias, record bias, incomplete data) | 1305 patients who were diagnosed with acute cholangitis (AC) according to the Tokyo guidelines 2018 | Different timing for biliary drainage (12 hours, 24 hours, 48 hours) |
| **Outcome Measurement/results** | In-hospital mortality (IHM), hospital length of stay (LOS), and hospitalization costs associated with the timing of biliary drainage according to the severity grading and different dysfunctioning organs and predictors [age, white blood cell (WBC) count, total bilirubin, albumin, lactate, malignant obstruction, and Charlson comorbidity index (CCI)] | Biliary drainage within 24 or 48 h in Grade III AC patients could dramatically decrease IHM (3.9% vs 9.0%, P= 0.041; 4% vs 9.9%, P= 0.018, respectively), while increasing LOS and hospitalization costs. Biliary decompression performed within 12 h of admission significantly decreased the IHM in AC patients with neurological dysfunction (0% vs 17.3%, P= 0.041) or with serum lactate > 2 mmol/L (0% vs 5.4%, P= 0.016). In the subgroup of AC patients with renal dysfunction, abnormal WBC count, hyperbilirubinemia, or hypoalbuminemia, early drainage (< 24 h) reduced the IHM (3.6% vs 33.3%, P= 0.004; 1.9% vs 5.8%, P= 0.031; 1.7% vs 5.0%, P= 0.019; 0% vs 27%, P= 0.026; respectively). The IHM was lower in patients with AC combined with hepatic dysfunction, malignant obstruction, or a CCI > 3 who had undergone biliary drainage within 48 h (2.6% vs 20.5%, P= 0.016; 3.0% vs 13.5%, P= 0.006; 3.4% vs 9.6%, P= 0.021; respectively) | |
| **Conclusions** | Biliary drainage within 12 h is beneficial for AC patients with neurological or cardiovascular dysfunction, while complete biliary decompression within 24 h of admission is recommended for treating patients with Grade III AC | | |

**11. Supplemental Files Table 10.** Supplementary Table Report Template Question 8.

**Research question 8. *Should surgical or endoscopic step-up approach vs. upfront necrosectomy be used as the first line of treatment for patients with pancreatic necrosis?***

| **Paper:** Treatment of disrupted and disconnected pancreatic duct in necrotizing pancreatitis: A systematic review and meta-analysis  Pancreatology 2019  Van Dijk SM, *et al.* | | | |
| --- | --- | --- | --- |
| **Study type/ evidence level** | **Study details/limitations** | **Patients’ characteristics** | **Interventions** |
| **Study type:** Systematic review and meta-analysis of observational studies  **Evidence level:** Low | ***Countries:*** Not applicable  ***Centers:*** Not applicable  ***Setting:*** Patients with disconnected pancreatic duct in necrotizing pancreatitis  ***Funding sources*:** None  ***Dropout rates:*** Not applicable  ***Limitations*:** The majority of included studies were retrospective by design and only presented results of a single treatment strategy | 583 patients with disrupted and disconnected pancreatic duct in necrotizing pancreatitis | Various treatment strategies |
| **Outcome Measurement/results** | Success rates | The most frequently used treatment strategies included endoscopic transpapillary drainage, endoscopic transluminal drainage, surgical drainage or resection, or combined procedures. Pooled analysis showed success rates of 81% (95%CI: 60-92%) for transpapillary and 92% (95%CI: 77-98%) for transluminal drainage, 80% (95%CI: 67-89%) for distal pancreatectomy and 84% (95%CI: 73-91%) for cyst-jejunostomy. Success rates did not differ between surgical procedures (cyst-jejunostomy and distal pancreatectomy (risk ratio 1.06, p= 0.26)) but distal pancreatectomy was associated with a higher incidence of endocrine pancreatic insufﬁciency (risk ratio 3.06, p= 0.01). The success rate of conservative treatment is unknown | |
| **Conclusions** | Different treatment strategies for pancreatic duct disruption and duct disconnection after necrotizing pancreatitis show high success rates | | |

| **Paper:** Treatment for Infected Pancreatic Necrosis Should be Delayed, Possibly Avoiding an Open Surgical Approach. A Systematic Review and Network Meta-analysis  Annals of surgery 2021  Ricci C, *et al.* | | | |
| --- | --- | --- | --- |
| **Study type/ evidence level** | **Study details/limitations** | **Patients’ characteristics** | **Interventions** |
| **Study type:** Systematic review and meta-analysis of randomized controlled trials  **Evidence level:** High | ***Countries:*** Not applicable  ***Centers:*** Not applicable  ***Setting:*** Patients with infected pancreatic necrosis  ***Funding sources*:** None  ***Dropout rates:*** Not applicable  ***Limitations*:** Heterogeneity | 400 patients with infected pancreatic necrosis | Seven studies were included, involving 400 patients clustered as following: 64 (16%) in early surgical debridement (ED); 27 (6.7%) in peritoneal lavage (PL); 45 (11.3%) in delayed surgical debridement (DD), 169 (42.3%) in the step-up approach with minimally invasive debridement (SUA-DD) and 95 (23.7%) with endoscopic debridement (SUA-EnD) |
| **Outcome Measurement/results** | in-hospital mortality and major morbidity rates (primary endpoints). The secondary endpoints were mortality, length of stay, intensive care unit stay, the pancreatic fistula rate, and exocrine and endocrine insufficiency | The step-up approach with endoscopic debridement had the highest probability of being the safest approach (SUCRA 87.1%), followed by SUA-DD (SUCRA 59.5%); DD, ED, and PL had the lowest probability of being safe (SUCRA values 27.6%, 31.4%, and 44.4%, respectively). Analysis of the secondary endpoints confirmed the superiority of SUA-EnD regarding length of stay, intensive care unit stay, pancreatic fistula rate, and new-onset diabetes. The SUA approaches are similar regarding exocrine function. Mortality was reduced by any delayed approaches (DD, SUA-DD, or SUA-EnD) | |
| **Conclusions** | The first choice for suspected infected pancreatic necrosis is endoscopic debridement. An alternative could be minimally-invasive debridement. Peritoneal lavage, early surgical debridement, and delayed surgical debridment should be avoided | | |

| **Paper:** Endoscopic and surgical drainage for pancreatic ﬂuid collections are better than percutaneous drainage: Meta-analysis  Pancreatology 2020  Szako L, *et al.* | | | |
| --- | --- | --- | --- |
| **Study type/ evidence level** | **Study details/limitations** | **Patients’ characteristics** | **Interventions** |
| **Study type:** Systematic review and meta-analysis of randomized and non-randomized studies  **Evidence level:** Moderate | ***Countries:*** Not applicable  ***Centers:*** Not applicable  ***Setting:*** Patients with pancreatic pseudocyst and walled-off necrosis  ***Funding sources*:** Supported by the ÚNKP-18-19-2 New National Excellence Program of the Ministry of Human Capacities, the Economic Development and Innovation Operative Programme Grant and the Human Resources Development Operational Programme Grants  ***Dropout rates:*** Not applicable  ***Limitations*:** Non-randomized studies were included, low-GRADE quality, heterogeneity, | Patients with pancreatic pseudocyst and walled-off necrosis | Patients were managed endoscopically, percutaneously or surgically |
| **Outcome Measurement/results** | Mortality, clinical success, recurrence, complications, cost and length of hospitalization (LOH) | The outcomes of endoscopic and percutaneous drainage were comparable in six articles. The clinical success of the endoscopic intervention was better considering any types of ﬂuid collections (OR 3.36; 95% conﬁdence interval (CI) 1.48, 7.63; p= 0.004). ED was preferable regarding the recurrence of PP (OR 0.23; 95% CI 0.08, 0.66; p= 0.006). Fifteen articles compared surgical intervention with ED. Signiﬁcant difference was found in postoperative LOH (WMD (days) (4.61; 95%CI -7.89, 1.33; p= 0.006) and total LOH (WMD (days) 3.67; 95%CI -5.00, 2.34; p< 0.001) which favored endoscopy, but ED had a lower rate of clinical success (OR 0.54; 95% CI 0.35, 0.85; p= 0.007) and a higher rate of recurrence (OR 1.80; 95% CI 1.16, 2.79; p= 0.009) in the treatment of PP. Eleven studies compared surgical and percutaneous interventions. PD resulted in a higher rate of recurrence (OR 4.91; 95% CI 1.82, 13.22; p= 0.002) and a lower rate of clinical success (OR 0.13; 95% CI 0.07, 0.22, p< 0.001) | |
| **Conclusions** | Both endoscopy and surgery are preferable over percutaneous intervention, furthermore endoscopic treatment is associated with shorter hospitalization than surgery | | |

| **Paper:** Endoscopic versus surgical treatment for infected necrotizing pancreatitis: a systematic review and meta‑analysis of randomized controlled trials  Surgical endoscopy 2020  Haney CM, *et al.* | | | |
| --- | --- | --- | --- |
| **Study type/ evidence level** | **Study details/limitations** | **Patients’ characteristics** | **Interventions** |
| **Study type:** Systematic review and meta-analysis of randomized controlled trials  **Evidence level:** Moderate | ***Countries:*** Not applicable  ***Centers:*** Not applicable  ***Setting:*** Patients with infected pancreatic necrosis  ***Funding sources*:** Funded by resources from the department of General, Visceral and Transplantation Surgery at the University of Heidelberg  ***Dropout rates: Not applicable***  ***Limitations*:** Small sample size (risk of imprecision), moderate overall certainty | 190 patients with infected pancreatic necrosis | Endoscopy (either step-up or non-step-up procedures) versus Surgery (either step-up or non-step-up procedures) |
| **Outcome Measurement/results** | Mortality and new onset multiple organ failure | Intention to treat analysis showed no difference in mortality. Patients in the endoscopic group had statistically significant lower odds of experiencing new onset multiple organ failure (odds ratio (OR) confidence interval [CI] 0.31 [0.10, 0.98]) and were statistically less likely to suffer from perforations of visceral organs or enterocutaneous fistulae (OR [CI] 0.31 [0.10, 0.93]), and pancreatic fistulae (OR [CI] 0.09 [0.03, 0.28]). Patients with endoscopic treatment had a statistically significant lower mean hospital stay (Mean difference [CI] − 7.86 days [− 14.49, − 1.22]). No differences in bleeding requiring intervention, incisional hernia, exocrine or endocrine insufficiency, or ICU stay were apparent | |
| **Conclusions** | There seem to be possible benefits of endoscopic treatment procedure | | |

| **Paper:** Minimally invasive drainage versus open surgical debridement in SAP/SMAP – a network meta-analysis  BMC Gastroenterology 2019  Zhang K, *et al.* | | | |
| --- | --- | --- | --- |
| **Study type/ evidence level** | **Study details/limitations** | **Patients’ characteristics** | **Interventions** |
| **Study type:** Systematic review and network meta-analysis  **Evidence level:** High | ***Countries:*** Not applicable  ***Centers:*** Not applicable  ***Setting:*** Patients with moderately-severe and severe acute pancreatitis with necrosis  ***Funding sources*:** Supported by grants from the Priority Academic Program Development of Jiangsu Higher Education Institutions, The Innovation Capability Development Project of Jiangsu Province and Jiangsu Biobank of Clinical Resources  ***Dropout rates:*** Not applicable  ***Limitations*:** Heterogeneity | 985 patients with moderately-severe and severe acute pancreatitis with necrosis | Participants were classed into 5 groups: conservative treatment (CST), early minimally invasive drainage (MID) (EMID), late MID (LMID), early open surgical drainage (OSD) (EOSD), and late OSD (LOSD) |
| **Outcome Measurement/results** | Mortality, multiple organ failure | Compared with CST, MID could decrease both mortality and multiple organ dysfunction syndrome (MODS) rate but OSD could not. Both EMID and MID can significantly decrease the mortality and MODS rate compared to CST. PCD might be most likely to have a benefit compared to CST | |
| **Conclusions** | Existing evidence for the use of MID in patients with moderately-severe and severe acute pancreatitis with necrosis is reliable and it can be used as early treatment. OSD, if necessary, should be avoided or delayed as long as possible | | |

| **Paper:** Endoscopic versus surgical treatment for pancreatic pseudocysts. Systematic review and meta-analysis  Medicine 2019  Farias GFA, *et al.* | | | |
| --- | --- | --- | --- |
| **Study type/ evidence level** | **Study details/limitations** | **Patients’ characteristics** | **Interventions** |
| **Study type:** Systematic review and meta-analysis of randomized and observational studies  **Evidence level:** Moderate | ***Countries:*** Not applicable  ***Centers:*** Not applicable  ***Setting:*** Patients with pancreatic pseudocysts  ***Funding sources*:** Not reported  ***Dropout rates:*** Not applicable  ***Limitations*:** The review also included non-randomized studies, different definitions of clinical success | 342 adult patients (>18 years) with pancreatic pseudocysts (PPs) requiring interventional treatment | Endoscopic treatment (intervention) versus surgical treatment (control) |
| **Outcome Measurement/results** | Therapeutic success, adverse events related to drainage, general adverse events, and recurrence (primary outcomes), cost and time of hospitalization (secondary outcomes) | There was no signiﬁcant difference between treatment success rate (risk difference [RD] -0.09; 95% conﬁdence interval [CI] [0.20,0.01]; P= 0.07), drainage-related adverse events (RD -0.02; 95% CI [-0.04,0.08]; P= 0.48), general adverse events (RD -0.05; 95% CI [0.12, 0.02]; P= 0.13) and recurrence (RD: 0.02; 95% CI [-0.04,0.07]; P= 0.58) between surgical and endoscopic treatment.  Regarding the length of hospitalization, the endoscopic group had better results (RD: -4.23; 95% CI [-5.18, -3.29]; P< 0.000001). The endoscopic arm also had better outcomes in terms of costs (RD: -4.68; 95% CI [-5.43,-3.94]; P< 0.00001) | |
| **Conclusions** | There is no signiﬁcant difference between surgical and endoscopic treatment success rates, adverse events, and recurrence for PP. Time of hospitalization and treatment costs are lower in the endoscopic group | | |

| **Paper:** Interventions for necrotising pancreatitis  Cochrane Collaboration 2016  Gurusamy KS, *et al.* | | | |
| --- | --- | --- | --- |
| **Study type/ evidence level** | **Study details/limitations** | **Patients’ characteristics** | **Interventions** |
| **Study type:** Systematic review and meta-analysis of randomized controlled trials  **Evidence level:** High | ***Countries:*** Not applicable  ***Centers:*** Not applicable  ***Setting:*** Patients with acute necrotizing pancreatitis  ***Funding sources*:** Supported by the National Institute for Health Research (NIHR) via Cochrane Infrastructure, Cochrane Programme Grant, or Cochrane Incentive funding to the Cochrane Hepato-Biliary and Upper Gastrointestinal and Pancreatic Diseases Groups  ***Dropout rates:*** Not applicable  ***Limitations*:** Low or very quality of the evidence, imprecision, inconsistency | 306 patients with necrotizing pancreatitis | Five trials (240 participants) investigated the three main treatments: open necrosectomy (121 participants), minimally invasive step-up approach (80 participants), and peritoneal lavage (39 participants). Three trials (66 participants) investigated the variations in the main treatments: early open necrosectomy (25 participants), delayed open necrosectomy (11 participants), video-assisted minimally invasive step-up approach (12 participants), endoscopic minimally invasive step-up approach (10 participants), minimally invasive step-up approach (planned surgery) (four participants), and minimally invasive step-up approach (continued percutaneous drainage) (four participants) |
| **Outcome Measurement/results** | Mortality, serious adverse events within six months, health-related quality of life | Adverse events were fewer in the minimally invasive step-up approach compared to open necrosectomy (serious adverse events: rate ratio 0.41, 95% CI 0.25 to 0.68; 88 participants; 1 study; adverse events: rate ratio 0.41, 95% CI 0.25 to 0.68; 88 participants; 1 study). The proportion of people with organ failure and the mean costs were lower in the minimally invasive step-up approach compared to open necrosectomy (organ failure: OR 0.20, 95% CI 0.07 to 0.60; 88 participants; 1 study; mean difference in costs: USD -11,922; P value < 0.05; 88 participants; 1 studies). There were more adverse events with video-assisted minimally invasive step-up approach group compared to endoscopic-assisted minimally invasive step-up approach group (rate ratio 11.70, 95% CI 1.52 to 89.87; 22 participants; 1 study), but the number of interventions per participant was less with video-assisted minimally invasive step-up approach group compared to endoscopic minimally invasive step-up approach group (difference in medians: 2 procedures; P value < 0.05; 20 participants; 1 study). The differences in any of the other comparisons for number of serious adverse events, the proportion of people with organ failure, number of adverse events, length of hospital stay, and intensive therapy unit stay were either imprecise or were not consistent | |
| **Conclusions** | The minimally invasive step-up approach resulted in fewer adverse events, serious adverse events, less organ failure, and lower costs compared to open necrosectomy. Very low-quality evidence suggested that the endoscopic minimally invasive step-up approach resulted in fewer adverse events than the video-assisted minimally invasive step-up approach but increased the number of procedures required for treatment. There is currently no evidence to suggest that early open necrosectomy is superior or inferior to peritoneal lavage or delayed open necrosectomy | | |

| **Paper:** Time for a Changing of Guard From Minimally Invasive Surgery to Endoscopic Drainage for Management of Pancreatic Walled-off Necrosis  Journal of Clinical Gastroenterology 2019  Khan MA, *et al.* | | | |
| --- | --- | --- | --- |
| **Study type/ evidence level** | **Study details/limitations** | **Patients’ characteristics** | **Interventions** |
| **Study type:** Systematic review and meta-analysis of randomized and non-randomized controlled trials  **Evidence level:** Moderate | ***Countries:*** Not applicable  ***Centers:*** Not applicable  ***Setting:*** Patients with walled-off pancreatic necrosis  ***Funding sources*:** None  ***Dropout rates:*** Not applicable  ***Limitations*:** Non-randomized studies were included in the pooled analysis | 641 patients with symptomatic sterile and infected pancreatic walled-off necrosis (WON) | Six studies (2 randomized controlled trials and 4 observational studies) with 641 patients (326 endoscopic drainage - ED and 315 minimally-invasive surgical necrosectomy - MISN) were included in this meta-analysis |
| **Outcome Measurement/results** | Mortality, major organ failure, adverse events, and length of hospital stay | Rates of mortality for ED and MISN were 8.5% and 14.2%, respectively. Pooled odds ratio (OR) with 95% conﬁdence interval was 0.59 (0.35-0.98), I2= 0% in favor of ED. On subgroup analysis: no difference in mortality was seen based on randomized controlled trials [OR, 0.65 (0.08-5.11)], while ED had improved survival in observational studies [OR, 0.49 (0.270.89)]. Development of new major organ failure rates after interventions were 12% and 54% for ED and MISN, respectively. Pooled OR was 0.12 (0.06-0.31), I2 = 25% in favor of ED. For adverse events, pooled OR was 0.25 (0.10-0.67), I2= 70% in favor of ED. There was no difference in risk of bleeding [OR, 0.68 (0.44-1.05)], while ED was associated with a signiﬁcantly lower rate of pancreatic ﬁstula formation [OR, 0.20 (0.11-0.37)], I2= 0%. Length of stay was also lower with ED. Pooled mean difference was −21.07 (−36.97 to −5.18) days | |
| **Conclusions** | ED is the preferred invasive management strategy over MISN for management of WON as it is associated with lower mortality, risk of major organ failure, adverse events, and length of hospital stay | | |

| **Paper:** Comparative efﬁcacy of stents in endoscopic ultrasonography-guided peripancreatic ﬂuid collection drainage: A systematic review and network meta-analysis  Journal of gastroenterology and hepatology 2020  Park CH, *et al.* | | | |
| --- | --- | --- | --- |
| **Study type/ evidence level** | **Study details/limitations** | **Patients’ characteristics** | **Interventions** |
| **Study type:** Systematic review and meta-analysis of randomized controlled trials and non-randomized studies  **Evidence level:** Moderate | ***Countries:*** Not applicable  ***Centers:*** Not applicable  ***Setting:*** Patients with peripancreatic ﬂuid collection (PFC)  ***Funding sources*:** None  ***Dropout rates:*** Not applicable  ***Limitations*:** The meta-analysis also included non-randomized studies, risk of population displacement, frequently in favor of the more recently developed metal stents (FCSEMS or LAMS) | 1746 patients with peripancreatic fluid collection | Double pigtail plastic stent (DPPS), fully covered self-expanding metal stent (FCSEMS), or lumen-apposing metal stent (LAMS) in EUS-guided PFC drainage |
| **Outcome Measurement/results** | Clinical success, recurrence, adverse events, bleeding risk, stent migration | In terms of clinical success, no signiﬁcant differences were noted in LAMS versus DPPS or LAMS versus FCSEMS (risk ratio [RR] 1.04 [95% credible interval (CrI) 0.991.11] and RR 0.96 [95% CrI 0.91–1.03]), respectively). FCSEMS was superior in clinical success to DPPS (RR 1.09, 95% CrI 1.02–1.15). There was no signiﬁcant difference in the recurrence of PFC among groups. Regarding adverse events, LAMS had a higher bleeding risk than FCSEMS (RR 6.70, 95% CrI 1.77–36.27) and tended to have a higher risk of bleeding than DPPS (RR 2.67, 95% CI 0.71–9.28). In terms of stent migration, there was no signiﬁcant difference between any two groups compared | |
| **Conclusions** | FCSEMS had superior efﬁcacy in terms of clinical success compared with DPPS stents. Signiﬁcant superiority of LAMS to DPPS was not identiﬁed. Additionally, LAMS had the higher risk of bleeding than FCSEMS | | |

| **Paper:** Endoscopic or surgical step-up approach for infected necrotising pancreatitis: a multicentre randomised trial  Lancet 2018  Von Brunschot S, *et al.* | | | |
| --- | --- | --- | --- |
| **Study type/ evidence level** | **Study details/limitations** | **Patients’ characteristics** | **Interventions** |
| **Study type:** Randomized controlled trial  **Evidence level:** High | ***Countries:*** Netherlands  ***Centers:*** Multicenter (Academic Medical Centre, University of Amsterdam, Amsterdam, Netherlands. Pilot Center)  ***Setting:*** Patients with infected pancreatic necrosis  ***Funding sources*:** The Dutch Digestive Disease Foundation, Fonds NutsOhra, and the Netherlands Organization for Health Research and Development  ***Dropout rates:*** 0%  ***Limitations*:** Still a relatively small sample size, the length of follow-up (6 months) could be too short to detect further benefits or complications of the endoscopic step-up approach in the long term | 98 patients with infected necrotizing pancreatitis | Patients were randomly assigned to the endoscopic step-up approach (n=51) or the surgical step-up approach (n=47) |
| **Outcome Measurement/results** | The primary endpoint was a composite of major complications or death during 6-month follow-up (analyses were by intention to treat) | The primary endpoint occurred in 22 (43%) of 51 patients in the endoscopy group and in 21 (45%) of 47 patients in the surgery group (risk ratio [RR] 0.97, 95% CI 0.62–1.51; p= 0.88). Mortality did not differ between groups (nine [18%] patients in the endoscopy group vs. six [13%] patients in the surgery group; RR 1.38, 95% CI 0.53–3.59, p= 0.50), nor did any of the major complications included in the primary endpoint | |
| **Conclusions** | In patients with infected necrotising pancreatitis, the endoscopic step-up approach was not superior to the surgical step-up approach in reducing major complications or death. The rate of pancreatic fistulas and length of hospital stay were lower in the endoscopy group | | |

| **Paper:** Endoscopic versus laparoscopic drainage of pseudocyst and walled‑off necrosis following acute pancreatitis: a randomized trial  Surgical endoscopy 2019  Garg PK, *et al.* | | | |
| --- | --- | --- | --- |
| **Study type/ evidence level** | **Study details/limitations** | **Patients’ characteristics** | **Interventions** |
| **Study type:** Randomized controlled trial  **Evidence level:** Moderate | ***Countries:*** India  ***Centers:*** Department of Gastroenterology and Department of Surgical Disciplines, All India Institute of Medical Sciences, New Delhi, India  ***Setting:*** Patients with pseudocyst or walled-off necrosis following acute pancreatitis  ***Funding sources*:** Grant from Indian Council of Medical Research  ***Dropout rates:*** 0%  ***Limitations*:** Small sample size (Imprecision), external validity (the trial included restricted use of EUS guidance and exclusion of patients with > 30% necrotic debris) | 60 patients with pseudocyst/walled-off necrosis following acute pancreatitis | Endoscopic drainage (per-oral transluminal cystogastrostomy and endoscopic lavage and necrosectomy) was done following a step-up approach for infected collections. Surgical laparoscopic cystogastrostomy was done for drainage, lavage, and necrosectomy |
| **Outcome Measurement/results** | Resolution of the pancreatic fluid collection by the intended modality (primary outcome) and complications (secondary outcome) | The initial success rate was 83.3% in the laparoscopic and 76.6% in the endoscopic group (p= 0.7) after the index intervention. The overall success rate of 93.3% (28/30) and 90% (27/30) in the laparoscopic and endoscopic groups, respectively, was also similar (p= 1.0). Two patients in the laparoscopic group required endoscopic cystogastrostomy for persistent collections. Similarly, two patients in the endoscopic group required laparoscopic drainage. Postoperative complications were comparable between the groups except for higher post-procedure infection in the endoscopic group (19 vs. 9; p= 0.01) requiring endoscopic re-intervention | |
| **Conclusions** | Endoscopic and laparoscopic techniques have similar efficacy for internal drainage of suitable pancreatic fluid collections with < 30% debris | | |

| **Paper:** Comparison of lumen-apposing metal stents versus double-pigtail plastic stents for infected necrotising pancreatitis  Gut 2023  Boxhoorn L, *et al.* | | | |
| --- | --- | --- | --- |
| **Study type/ evidence level** | **Study details/limitations** | **Patients’ characteristics** | **Interventions** |
| **Study type:** Comparison of two prospective cohorts (non-randomized study)  **Evidence level:** Moderate | ***Countries:*** Netherlands  ***Centers:*** Multicenter (Gastroenterology and Hepatology, Amsterdam UMC, University of Amsterdam, Amsterdam, The Netherlands. Coordinating center)  ***Setting:*** Patients with infected necrotizing pancreatitis  ***Funding sources*:** Funded by Boston Scientific Corporation and Amsterdam UMC  ***Dropout rates:*** 0%  ***Limitations*:** Non-randomized design, possible presence of confounding effects, not powered to detect differences in complications | Patients with infected necrotizing pancreatitis | 53 patients with infected necrotizing pancreatitis, who underwent an endoscopic step-up approach with LAMS within a multicentre prospective cohort study were compared with the data of 51 patients in the randomized TENSION trial who had been assigned to the endoscopic step-up approach with double-pigtail plastic stents |
| **Outcome Measurement/results** | Primary end point was the need for endoscopic transluminal necrosectomy. Secondary end points included mortality, major complications, hospital stay and healthcare costs | The need for endoscopic transluminal necrosectomy was 64% (n=34) and was not different from the previous trial using plastic stents (53%, n=27)), also after correction for baseline characteristics (OR 1.21 (95% CI 0.45 to 3.23)). Secondary endpoints did not differ between groups either, which also included bleeding requiring intervention -5 patients (9%) after LAMS placement vs. 11 patients (22%) after placement of plastic stents (relative risk 0.44; 95% CI 0.16 to 1.17). Total healthcare costs were also comparable (mean difference −€6348, bias-corrected and accelerated 95% CI −€26386 to €10121) | |
| **Conclusions** | LAMS do not reduce the need for endoscopic transluminal necrosectomy when compared with double-pigtail plastic stents in patients with infected necrotising pancreatitis. Also, the rate of bleeding complications was comparable | | |

| **Paper:** A Step-up Approach or Open Necrosectomy for Necrotizing Pancreatitis  The New England Journal of Medicine 2010  Van Santvoort HC, *et al.* | | | |
| --- | --- | --- | --- |
| **Study type/ evidence level** | **Study details/limitations** | **Patients’ characteristics** | **Interventions** |
| **Study type:** Randomized controlled trial  **Evidence level:** High | ***Countries:*** Netherlands  ***Centers:*** Multicentric (7 university medical centers and 12 large teaching hospitals of the Dutch Pancreatitis Study Group)  ***Setting:*** Patients with necrotizing pancreatitis and suspected or confirmed infected necrotic tissue  ***Funding sources*:** Supported by a grant from the Dutch Organization for Health Research and Development  ***Dropout rates:*** 0%  ***Limitations*:** - | 88 patients with necrotizing pancreatitis and suspected or confirmed infected necrotic tissue | to undergo primary open necrosectomy or a step-up approach to treatment. |
| **Outcome Measurement/results** | The primary end point was a composite of major complications (new-onset multiple-organ failure or multiple systemic complications, perforation of a visceral organ or enterocutaneous fistula, or bleeding) or death | The primary endpoint occurred in 31 of 45 patients (69%) assigned to open necrosectomy and in 17 of 43 patients (40%) assigned to the step-up approach (risk ratio with the step-up approach, 0.57; 95% confidence interval, 0.38 to 0.87; P= 0.006). Of the patients assigned to the step-up approach, 35% were treated with percutaneous drainage only. New-onset multiple-organ failure occurred less often in patients assigned to the step-up approach than those assigned to open necrosectomy (12% vs. 40%, P= 0.002). The rate of death did not differ significantly between groups (19% vs. 16%, P= 0.70). Patients assigned to the step-up approach had a lower rate of incisional hernias (7% vs. 24%, P= 0.03) and new-onset diabetes (16% vs. 38%, P= 0.02) | |
| **Conclusions** | The minimally invasive step-up approach, as compared with open necrosectomy, reduces the rate of the composite end point of major complications or death among patients with necrotizing pancreatitis and infected necrotic tissue | | |

| **Paper:** Immediate versus Postponed Intervention for Infected Necrotizing Pancreatitis  The New England Journal of Medicine 2021  Boxhoorn L, *et al.* | | | |
| --- | --- | --- | --- |
| **Study type/ evidence level** | **Study details/limitations** | **Patients’ characteristics** | **Interventions** |
| **Study type:** Randomized controlled trial  **Evidence level:** High | ***Countries:*** the Netherlands  ***Centers:*** Multicentric (the Dutch Pancreatitis Study Group)  ***Setting:*** Patients with infected necrotizing pancreatitis  ***Funding sources*:** Supported by Fonds NutsOhra, the Netherlands, and the Amsterdam UMC, University of Amsterdam  ***Dropout rates:*** 0%  ***Limitations*:** the trial protocol allowed for both endoscopic and surgical step-up approaches | 104 patients with infected pancreatic necrosis | Patients were randomly assigned to immediate drainage (55 patients) or postponed drainage (49 patients) |
| **Outcome Measurement/results** | The primary end point was the score on the Comprehensive Complication Index, which incorporates all complications over the course of 6 months of follow-up | The mean score on the Comprehensive Complication Index (scores range from 0 to 100, with higher scores indicating more severe complications) was 57 in the immediate-drainage group and 58 in the postponed-drainage group (mean difference, −1; 95% confidence interval [CI], −12 to 10; P= 0.90). Mortality was 13% in the immediate-drainage group and 10% in the postponed-drainage group (relative risk, 1.25; 95% CI, 0.42 to 3.68). The mean number of interventions (catheter drainage and necrosectomy) was 4.4 in the immediate-drainage group and 2.6 in the postponed-drainage group (mean difference, 1.8; 95% CI, 0.6 to 3.0). In the postponed-drainage group, 19 patients (39%) were treated conservatively with antibiotics and did not require drainage; 17 patients survived. The incidence of adverse events was similar in the two groups | |
| **Conclusions** | This trial did not show the superiority of immediate drainage over postponed drainage with regard to complications in patients with infected necrotizing pancreatitis. Patients randomly assigned to the postponed-drainage strategy received fewer invasive interventions | | |

| **Paper:** An Endoscopic Transluminal Approach, Compared With Minimally Invasive Surgery, Reduces Complications and Costs for Patients With Necrotizing Pancreatitis  Gastroenterology 2019  Bang JY, *et al.* | | | |
| --- | --- | --- | --- |
| **Study type/ evidence level** | **Study details/limitations** | **Patients’ characteristics** | **Interventions** |
| **Study type:** Randomized controlled trial  **Evidence level:** High | ***Countries:*** USA  ***Centers:*** Florida Hospital, Orlando (USA)  ***Setting:*** Patients with conﬁrmed or suspected infected necrotizing pancreatitis  ***Funding sources*:** None  ***Dropout rates:*** 0%  ***Limitations*:** - | 66 patients with conﬁrmed or suspected infected necrotizing pancreatitis who required intervention from May 12, 2014, through March 24, 2017 | Patients were randomly assigned to groups that received minimally invasive surgery (laparoscopic or video-assisted retroperitoneal debridement, depending on location of collection, n= 32) or an endoscopic step-up approach (transluminal drainage with or without necrosectomy, n= 34) |
| **Outcome Measurement/results** | The primary endpoint was a composite of major complications (new-onset multiple organ failure, new-onset systemic dysfunction, enteral or pancreatic-cutaneous ﬁstula, bleeding and perforation of a visceral organ) or death during 6 months of follow-up | The primary endpoint occurred in 11.8% of patients who received the endoscopic procedure and 40.6% of patients who received the minimally invasive surgery (risk ratio 0.29; 95% conﬁdence interval 0.11–0.80; P= 0.007). Although there was no signiﬁcant difference in mortality (endoscopy 8.8% vs. surgery 6.3%; P= 0.999), none of the patients assigned to the endoscopic approach developed enteral or pancreatic-cutaneous ﬁstulae compared with 28.1% of the patients who underwent surgery (P= 0.001). The mean number of major complications per patient was signiﬁcantly higher in the surgery group (0.69 ± 1.03) compared with the endoscopy group (0.15 ± 0.44) (P= 0.007). The physical health scores for quality of life at 3 months were better with the endoscopic approach (P= 0.039), and mean total cost was lower ($75,830) compared with $117,492 for surgery (P= 0.039) | |
| **Conclusions** | In a randomized trial of 66 patients, an endoscopic transluminal approach for infected necrotizing pancreatitis, compared with minimally invasive surgery, signiﬁcantly reduced major complications, lowered costs, and increased quality of life | | |

| **Paper:** A Conservative and Minimally Invasive Approach to Necrotizing Pancreatitis Improves Outcome  Gastroenterology 2011  Van Santvoort HC, *et al.* | | | |
| --- | --- | --- | --- |
| **Study type/ evidence level** | **Study details/limitations** | **Patients’ characteristics** | **Interventions** |
| **Study type:** Prospective observational study  **Evidence level:** Moderate | ***Countries:*** the Netherlands  ***Centers:*** Multicentric (the Dutch Pancreatitis Study Group)  ***Setting:*** Patients with necrotizing pancreatitis  ***Funding sources*:** Supported by a research grant from the Dutch Organization for Health Research and Development  ***Dropout rates:*** Not applicable  ***Limitations*:** Non-randomized design, heterogeneity in the diagnostic process (CT scan) | This study collected data from 639 consecutive patients with necrotizing pancreatitis, from 2004 to 2008, treated at 21 Dutch hospitals | Different interventions (radiologic, endoscopic, surgical) |
| **Outcome Measurement/results** | Mortality, organ failure | Overall mortality was 15%. Organ failure occurred in 240 patients (38%), with 35% mortality. Treatment was conservative in 397 patients (62%), with 7% mortality. An intervention was performed in 242 patients (38%), with 27% mortality; this included early emergency laparotomy in 32 patients (5%), with 78% mortality. Patients with longer times between admission and intervention had lower mortality: 0 to 14 days, 56%; 14 to 29 days, 26%; and >29 days, 15% (P= 0.001). A total of 208 patients (33%) received interventions for infected necrosis, with 19% mortality. Catheter drainage was most often performed as the ﬁrst intervention (63% of cases), without additional necrosectomy in 35% of patients. Primary catheter drainage had fewer complications than primary necrosectomy (42% vs 64%, P= 0.003). Patients with pancreatic parenchymal necrosis (n= 324), compared with patients with only peripancreatic necrosis (n= 315), had a higher risk of organ failure (50% vs 24%, P= 0.001) and mortality (20% vs 9%, P= 0.001) | |
| **Conclusions** | Approximately 62% of patients with necrotizing pancreatitis can be treated without an intervention and with low mortality. In patients with infected necrosis, delayed intervention and catheter drainage as ﬁrst treatment improves outcome | | |

| **Paper:** Superiority of Step-up Approach vs Open Necrosectomy in Long-term Follow-up of Patients With Necrotizing Pancreatitis  Gastroenterology 2019  Hollemans RA, *et al.* | | | |
| --- | --- | --- | --- |
| **Study type/ evidence level** | **Study details/limitations** | **Patients’ characteristics** | **Interventions** |
| **Study type:** Randomized controlled trial  **Evidence level:** High | ***Countries:*** the Netherlands  ***Centers:*** Multicentric (the Dutch Pancreatitis Study Group)  ***Setting:*** Patients with necrotizing pancreatitis  ***Funding sources*:** Funded by the Dutch Digestive Disease Foundation  ***Dropout rates:*** 30.2% (step-up) vs. 20.0% (open necrosectomy)  ***Limitations*:** - | This study followed up 73 patients with necrotizing pancreatitis (of the 88 patients randomly assigned to groups in the PANTER trial) who were still alive after the index admission at a mean of 86 months (± 11 months) of follow-up | Minimally-invasive step-up approach versus open necrosectomy |
| **Outcome Measurement/results** | The primary endpoint was death or major complications. Exocrine insufﬁciency, quality of life (using the Short Form-36 and EuroQol 5 dimensions forms), and Izbicki pain scores were evaluated | From index admission to long-term follow-up, 19 patients (44%) died or had major complications in the step-up group compared with 33 patients (73%) in the open-necrosectomy group (P= 0.005).  A significantly lower proportion of patients in the step-up group had incisional hernias (23% vs. 53%; P= 0.004), pancreatic exocrine insufﬁciency (29% vs. 56%; P= 0.03), or endocrine insufﬁciency (40% vs. 64%; P= 0.05). There were no signiﬁcant differences between groups in proportions of patients requiring additional drainage procedures (11% vs. 13%; P= 0.99) or pancreatic surgery (11% vs. 5%; P= 0.43), or in recurrent acute pancreatitis, chronic pancreatitis, Izbicki pain scores, or medical costs. Quality of life increased during follow-up without a signiﬁcant difference between groups | |
| **Conclusions** | In an analysis of long-term outcomes of trial participants, the step-up approach for necrotizing pancreatitis was superior to open necrosectomy | | |

| **Paper:** Endoscopic Versus Surgical Step-Up Approach for Infected Necrotizing Pancreatitis (ExTENSION): Long-term Follow-up of a Randomized Trial  Gastroenterology 2022  Onnekink AM, *et al.* | | | |
| --- | --- | --- | --- |
| **Study type/ evidence level** | **Study details/limitations** | **Patients’ characteristics** | **Interventions** |
| **Study type:** Randomized controlled trial  **Evidence level:** High | ***Countries:*** the Netherlands  ***Centers:*** Multicentric (the Dutch Pancreatitis Study Group)  ***Setting:*** Patients with infected pancreatic necrosis  ***Funding sources*:** Not reported  ***Dropout rates:*** 0%  ***Limitations*:** quality of life was not assessed directly after randomization (at baseline) and at predeﬁned time points after the initial 6-month follow-up; the long-term follow-up period was not standardized between patients | In this long-term follow-up study, clinical data of 83 patients with infected pancreatic necrosis (of the originally 98 included patients) from the TENSION trial who were still alive after the initial 6-month follow-up were evaluated | Endoscopic versus surgical step-up approach |
| **Outcome Measurement/results** | The primary end point was a composite of death and major complications. Secondary end points included individual major complications, pancreaticocutaneous ﬁstula, reinterventions, pancreatic insufﬁciency, and quality of life | After a mean follow-up period of 7 years, the primary end point occurred in 27 patients (53%) in the endoscopy group and 27 patients (57%) in the surgery group (risk ratio [RR], 0.93; 95% conﬁdence interval [CI], 0.65–1.32; P= 0.688). Fewer pancreaticocutaneous ﬁstulas were identiﬁed in the endoscopy group (8% vs. 34%; RR, 0.23; 95% CI, 0.08–0.83). After the initial 6-month follow-up, the endoscopy group needed fewer reinterventions than the surgery group (7% vs. 24%; RR, 0.29; 95% CI, 0.09–0.99). Pancreatic insufﬁciency and quality of life did not differ between groups | |
| **Conclusions** | At long-term follow-up, the endoscopic step-up approach was not superior to the surgical step-up approach in reducing death or major complications in patients with infected necrotizing pancreatitis. Patients assigned to the endoscopic approach developed overall fewer pancreaticocutaneous ﬁstulas and needed fewer reinterventions after the initial 6-month follow-up | | |

| **Paper:** Timing of surgical intervention in patients of infected necrotizing pancreatitis not responding to percutaneous catheter drainage  Pancreatology 2016  Shenvi S, *et al.* | | | |
| --- | --- | --- | --- |
| **Study type/ evidence level** | **Study details/limitations** | **Patients’ characteristics** | **Interventions** |
| **Study type:** Randomized controlled trial  **Evidence level:** Low | ***Countries:*** India  ***Centers:*** Division of Surgical Gastroenterology and the Department of Gastroenterology, Postgraduate Institute of Medical Education & Research, Chandigarh, India  ***Setting:*** Patients with infected necrotizing pancreatitis  ***Funding sources*:** Not reported  ***Dropout rates:*** 0%  ***Limitations*:** Small sample size (risk of imprecision), early termination | 40 patients with infected pancreatic necrosis | Patients were randomized to group A (step-up approach as a bridge to surgery) or group B (step-up approach with the intention to avoid surgery) |
| **Outcome Measurement/results** | Weekly inﬂammatory and nutritional markers were monitored in both groups | The trial was stopped prematurely because of difﬁculty in accrual and poor progress (8 patients were enrolled). All subsequent patients were managed with step-up approach with the intention to avoid surgery. Of 35 patients, 24 patients were managed by PCD alone while 11 patients required surgery. In patients who did not require surgery; levels of serum high sensitivity C-reactive protein (hsCRP), interleukin-6(IL6) and prealbumin showed a falling trend. This group also had higher baseline albumin and higher albumin at 4 weeks | |
| **Conclusions** | Step-up approach with the intention to avoid surgery led to a success rate of 68.5% | | |

**12. Supplemental Files Table 11.** **Supplementary Table Report Template Question 9.** **Research question 9. *Should delayed (after 4 weeks) therapeutic interventions (endoscopic or surgical step-up approach, surgical necrosectomy) vs. early interventions be used for patients with acute biliary necrotizing pancreatitis who remain clinically stable?***

| **Paper:** Interventions for necrotising pancreatitis  Cochrane Collaboration 2016  Gurusamy KS, *et al.* | | | |
| --- | --- | --- | --- |
| **Study type/ evidence level** | **Study details/limitations** | **Patients’ characteristics** | **Interventions** |
| **Study type:** Systematic review and meta-analysis of randomized controlled trials  **Evidence level:** High | ***Countries:*** Not applicable  ***Centers:*** Not applicable  ***Setting:*** Patients with acute necrotizing pancreatitis  ***Funding sources*:** Supported by the National Institute for Health Research (NIHR) via Cochrane Infrastructure, Cochrane Programme Grant, or Cochrane Incentive funding to the Cochrane Hepato-Biliary and Upper Gastrointestinal and Pancreatic Diseases Groups  ***Dropout rates:*** Not applicable  ***Limitations*:** Low or very quality of the evidence, imprecision, inconsistency | 306 patients with necrotizing pancreatitis | Five trials (240 participants) investigated the three main treatments: open necrosectomy (121 participants), minimally invasive step-up approach (80 participants), and peritoneal lavage (39 participants). Three trials (66 participants) investigated the variations in the main treatments: early open necrosectomy (25 participants), delayed open necrosectomy (11 participants), video-assisted minimally invasive step-up approach (12 participants), endoscopic minimally invasive step-up approach (10 participants), minimally invasive step-up approach (planned surgery) (four participants), and minimally invasive step-up approach (continued percutaneous drainage) (four participants) |
| **Outcome Measurement/results** | Mortality, serious adverse events within six months, health-related quality of life | Adverse events were fewer in the minimally invasive step-up approach compared to open necrosectomy (serious adverse events: rate ratio 0.41, 95% CI 0.25 to 0.68; 88 participants; 1 study; adverse events: rate ratio 0.41, 95% CI 0.25 to 0.68; 88 participants; 1 study). The proportion of people with organ failure and the mean costs were lower in the minimally invasive step-up approach compared to open necrosectomy (organ failure: OR 0.20, 95% CI 0.07 to 0.60; 88 participants; 1 study; mean difference in costs: USD -11,922; P value < 0.05; 88 participants; 1 studies). There were more adverse events with video-assisted minimally invasive step-up approach group compared to endoscopic-assisted minimally invasive step-up approach group (rate ratio 11.70, 95% CI 1.52 to 89.87; 22 participants; 1 study), but the number of interventions per participant was less with video-assisted minimally invasive step-up approach group compared to endoscopic minimally invasive step-up approach group (difference in medians: 2 procedures; P value < 0.05; 20 participants; 1 study). The differences in any of the other comparisons for number of serious adverse events, the proportion of people with organ failure, number of adverse events, length of hospital stay, and intensive therapy unit stay were either imprecise or were not consistent | |
| **Conclusions** | The minimally invasive step-up approach resulted in fewer adverse events, serious adverse events, less organ failure, and lower costs compared to open necrosectomy. Very low-quality evidence suggested that the endoscopic minimally invasive step-up approach resulted in fewer adverse events than the video-assisted minimally invasive step-up approach but increased the number of procedures required for treatment. There is currently no evidence to suggest that early open necrosectomy is superior or inferior to peritoneal lavage or delayed open necrosectomy | | |

| **Paper:** Early versus delayed interventions for necrotizing pancreatitis: A systematic review and meta-analysis  DEN open 2022  Nakai Y, *et al.* | | | |
| --- | --- | --- | --- |
| **Study type/ evidence level** | **Study details/limitations** | **Patients’ characteristics** | **Interventions** |
| **Study type:** Systematic review and meta-analysis of retrospective cohort studies  **Evidence level:** Moderate | ***Countries:*** Not applicable  ***Centers:*** Not applicable  ***Setting:*** Patients with necrotizing pancreatitis  ***Funding sources*:** None  ***Dropout rates:*** Not applicable  ***Limitations*:** Pooled analysis of retrospective cohort studies (selection bias), small sample size (imprecision), high heterogeneity for some endpoints | Patients with acute necrotizing pancreatitis | 11 retrospective studies were identified, including 775 patients with early interventions and 725 patients with delayed interventions (≤4 vs. >4 weeks) |
| **Outcome Measurement/results** | Adverse events, mortality, technical success, clinical success | Patients with early interventions tended to be complicated by organ failure. The rate of adverse events was comparable (OR 1.41, 95% CI 0.66–3.01; p= 0.38) but the rate of mortality was signiﬁcantly higher (OR 1.70, 95% CI 1.21–2.40; p< 0.01) in early interventions. Technical success rates were similarly high but clinical success rates tended to be low (OR 0.39, 95% CI 0.15–1.00; p = 0.05) in early interventions, though not statistically signiﬁcant. Pooled ORs for necrosectomy and open surgery were 2.14 and 1.23, respectively | |
| **Conclusions** | Early interventions for necrotizing pancreatitis are associated with higher mortality rates and do not reduce adverse events or improve clinical success | | |

| **Paper:** Early endoscopic drainage of PNC is feasible and seems to be safe as well as effective but is associated with increased risk of complications as compared to delayed drainage  Journal of gastroenterology 2022  Gao L, *et al.* | | | |
| --- | --- | --- | --- |
| **Study type/ evidence level** | **Study details/limitations** | **Patients’ characteristics** | **Interventions** |
| **Study type:** Systematic review and meta-analysis of randomized and non-randomized studies  **Evidence level:** Moderate | ***Countries:*** Not applicable  ***Centers:*** Not applicable  ***Setting:*** Patients with infected pancreatic necrosis  ***Funding sources*:** Supported by National Natural Science Foundation of China and Applied Basic Research Project of PLA  ***Dropout rates:*** Not applicable  ***Limitations*:** The pooled analysis included both randomized and non-randomized studies, small sample size (imprecision) | 742 patients with infected pancreatic necrosis (IPN) | Seven clinical studies were included with a total of 742 patients with IPN requiring intervention, of whom 321 received early intervention and 421 delayed intervention |
| **Outcome Measurement/results** | Mortality, hospital stay, complications | Early minimally invasive intervention did not increase hospital mortality (odds ratio 1.65, 95% conﬁdence interval 0.97–2.81; p= 0.06) but was associated with a remarkably prolonged hospital stay and an increased incidence of gastrointestinal ﬁstula or perforation when compared with delayed intervention | |
| **Conclusions** | It does appear that timing of intervention is a risk factor for adverse outcomes | | |

| **Paper:** Treatment for Infected Pancreatic Necrosis Should be Delayed, Possibly Avoiding an Open Surgical Approach. A Systematic Review and Network Meta-analysis  Annals of surgery 2021  Ricci C, *et al.* | | | |
| --- | --- | --- | --- |
| **Study type/ evidence level** | **Study details/limitations** | **Patients’ characteristics** | **Interventions** |
| **Study type:** Systematic review and meta-analysis of randomized controlled trials  **Evidence level:** High | ***Countries:*** Not applicable  ***Centers:*** Not applicable  ***Setting:*** Patients with infected pancreatic necrosis  ***Funding sources*:** None  ***Dropout rates:*** Not applicable  ***Limitations*:** Heterogeneity | 400 patients with infected pancreatic necrosis | Seven studies were included, involving 400 patients clustered as following: 64 (16%) in early surgical debridement (ED); 27 (6.7%) in peritoneal lavage (PL); 45 (11.3%) in delayed surgical debridement (DD), 169 (42.3%) in the step-up approach with minimally invasive debridement (SUA-DD) and 95 (23.7%) with endoscopic debridement (SUA-EnD) |
| **Outcome Measurement/results** | in-hospital mortality and major morbidity rates (primary endpoints). The secondary endpoints were mortality, length of stay, intensive care unit stay, the pancreatic fistula rate, and exocrine and endocrine insufficiency | The step-up approach with endoscopic debridement had the highest probability of being the safest approach (SUCRA 87.1%), followed by SUA-DD (SUCRA 59.5%); DD, ED, and PL had the lowest probability of being safe (SUCRA values 27.6%, 31.4%, and 44.4%, respectively). Analysis of the secondary endpoints confirmed the superiority of SUA-EnD regarding length of stay, intensive care unit stay, pancreatic fistula rate, and new-onset diabetes. The SUA approaches are similar regarding exocrine function. Mortality was reduced by any delayed approaches (DD, SUA-DD, or SUA-EnD) | |
| **Conclusions** | The first choice for suspected infected pancreatic necrosis is endoscopic debridement. An alternative could be minimally-invasive debridement. Peritoneal lavage, early surgical debridement, and delayed surgical debridment should be avoided | | |

| **Paper:** Comparison of early and delayed EUS-guided drainage of pancreatic fluid collection  Endoscopy international open 2018  Chantarojanasiri T, *et al.* | | | |
| --- | --- | --- | --- |
| **Study type/ evidence level** | **Study details/limitations** | **Patients’ characteristics** | **Interventions** |
| **Study type:** Retrospective cohort study  **Evidence level:** Low | ***Countries:*** Japan  ***Centers:*** University of Tokyo Hospitals, Japan  ***Setting:*** Patients with infected or symptomatic pancreatic fluid collections  ***Funding sources*:** Not reported  ***Dropout rates:*** Not applicable  ***Limitations*:** Non-randomized design (selection bias), small sample size (imprecision) | 35 consecutive patients who received EUS-guided drainage (EUS-PCD) of infected or symptomatic pancreatic fluid collections (PFC) at the University of Tokyo | Early vs. delayed (≥ 4 weeks) EUS-PCD were compared |
| **Outcome Measurement/results** | Technical success, adverse events, mortality | A total of 35 patients underwent EUS-PCD (12 early and 23 delayed) using 19 large-bore fully-covered metallic stent and 16 plastic stents. The median diameter of PFC was 110 mm (40 – 180) and 122 mm (17– 250) in the early and delayed drainage groups, respectively. Median time from onset of acute pancreatitis to drainage was 23 and 85 days for early and delayed drainage, respectively. The technical success rate of EUS-guided drainage was 100%. Endoscopic necrosectomy was performed in six early and 16 cases of delayed drainage. The adverse event rate was 25% (3 bleeding) and 13% (2 perforations and 1 CO 2 retention) in the early and delayed drainage groups, respectively. Two patients died (1 early and 1 delayed) due to multiorgan failure | |
| **Conclusions** | Endoscopic drainage and subsequent necrosectomy of symptomatic pancreatic fluid collections within 4 weeks after the onset of acute pancreatitis is feasible, given that the collection is encapsulated and attached to the gastrointestinal tract | | |

| **Paper:** Timing of surgical intervention in patients of infected necrotizing pancreatitis not responding to percutaneous catheter drainage  Pancreatology 2016  Shenvi S, *et al.* | | | |
| --- | --- | --- | --- |
| **Study type/ evidence level** | **Study details/limitations** | **Patients’ characteristics** | **Interventions** |
| **Study type:** Randomized controlled trial  **Evidence level:** Low | ***Countries:*** India  ***Centers:*** Division of Surgical Gastroenterology and the Department of Gastroenterology, Postgraduate Institute of Medical Education & Research, Chandigarh, India  ***Setting:*** Patients with infected necrotizing pancreatitis  ***Funding sources*:** Not reported  ***Dropout rates:*** 0%  ***Limitations*:** Small sample size (risk of imprecision), early termination | 40 patients with infected pancreatic necrosis | Patients were randomized to group A (step-up approach as a bridge to surgery) or group B (step-up approach with the intention to avoid surgery) |
| **Outcome Measurement/results** | Weekly inﬂammatory and nutritional markers were monitored in both groups | The trial was stopped prematurely because of difﬁculty in accrual and poor progress (8 patients were enrolled). All subsequent patients were managed with step-up approach with the intention to avoid surgery. Of 35 patients, 24 patients were managed by PCD alone while 11 patients required surgery. In patients who did not require surgery; levels of serum high sensitivity C-reactive protein (hsCRP), interleukin-6(IL6) and prealbumin showed a falling trend. This group also had higher baseline albumin and higher albumin at 4 weeks | |
| **Conclusions** | Step-up approach with the intention to avoid surgery led to a success rate of 68.5% | | |

| **Paper:** Surgical management of pancreatic necrosis: towards lesser and later  Journal of hepatobiliary and pancreatic science 2010  Cheung MT, *et al.* | | | |
| --- | --- | --- | --- |
| **Study type/ evidence level** | **Study details/limitations** | **Patients’ characteristics** | **Interventions** |
| **Study type:** Retrospective cohort study  **Evidence level:** Low | ***Countries:*** Hong Kong  ***Centers:*** Department of Surgery, Queen Elizabeth Hospital, Gascoigne Road, Kowloon, Hong Kong  ***Setting:*** Patients with acute pancreatic necrosis  ***Funding sources*:** Not reported  ***Dropout rates:*** Not applicable  ***Limitations*:** Non-randomized design (selection bias), small sample size (imprecision) | 26 patients with pancreatic necrosis | Early intervention (< 6 weeks) versus delayed intervention (beyond 6 weeks) |
| **Outcome Measurement/results** | Overall success rate of percutaneous pancreatic necrosectomy (PCPN) and survival rate with respect to the timing of intervention | The overall mortality rate was26.9% and the rate was signiﬁcantly higher in those patients who had earlier intervention (before 6 weeks). Eleven patients had PCPN. There were 2 failures due to PCPN in the early phase; 2 had partial success, while the procedure was completely successful to remove all the necrotic tissues in the other 7 patients | |
| **Conclusions** | Active intervention preferably should be delayed until the necrosis has become walled off, when a variety of minimally invasive maneuvers, notably percutaneous necrosectomy, can be offered to remove the debris. The surgical management of pancreatic necrosis should change towards a strategy of ‘‘lesser and later’’ | | |

| **Paper:** Delayed endoscopic necrosectomy improves hospital length of stay and reduces endoscopic interventions in patients with symptomatic walled-off necrosis  DEN open 2022  Pawa R, *et al.* | | | |
| --- | --- | --- | --- |
| **Study type/ evidence level** | **Study details/limitations** | **Patients’ characteristics** | **Interventions** |
| **Study type:** Retrospective cohort study  **Evidence level:** Low | ***Countries:*** USA  ***Centers:*** Department of Medicine, Division of Gastroenterology, Wake Forest University School of Medicine, Medical Center Boulevard, Winston-Salem, USA  ***Setting:*** Patients with symptomatic walled-off necrosis  ***Funding sources*:** None  ***Dropout rates:*** Not applicable  ***Limitations*:** Retrospective nature of the study (selection bias) and data from a single large tertiary care center (lack of generalizability) | 80 patients with symptomatic walled-off necrosis | Patients undergoing immediate DEN (iDEN) versus delayed DEN (dDEN). Patients with systemic inﬂammatory response syndrome (SIRS) within 1 week prior to endoscopic drainage underwent delayed necrosectomy (dDEN) at least 1 week after initial stent placement. If SIRS was absent in the week prior to endoscopic drainage, necrosectomy was performed at the time of initial LAMS placement (iDEN). |
| **Outcome Measurement/results** | Technical success, clinical success, number of necrosectomies, length of hospital stay | A total of 80 patients underwent DEN for the management of walled-off necrosis (iDEN = 43, dDEN = 37). Technical success was achieved in all patients. Clinical success was seen in 39 (91%) patients in the iDEN group and 34 (92%) in the dDEN group. Amongst iDEN patients, the mean number of necrosectomies was 2.5 (standard deviation [SD] 1.4) in comparison to 1.5 (SD 1.0) for dDEN (p= 0.0011). The median index hospital length of stay was longer with iDEN than dDEN (7.5 days vs. 3.0 days, respectively, p= 0.010). Subgroup analysis was performed based on the percentage of necrosis (<25% vs. >25% necrosis). iDEN was associated with more necrosectomies than dDEN regardless of the percentage of necrosis (p= 0.017 and 0.0067, respectively) | |
| **Conclusions** | Patients undergoing delayed endoscopic necrosectomy had a shorter index hospital stay and fewer necrosectomies than iDEN | | |

| **Paper:** How to identify the indications for early intervention in acute necrotizing pancreatitis patients: a long-term follow-up study  Frontiers in surgery 2022  Lu J, *et al.* | | | |
| --- | --- | --- | --- |
| **Study type/ evidence level** | **Study details/limitations** | **Patients’ characteristics** | **Interventions** |
| **Study type:** Retrospective cohort study  **Evidence level:** Low | ***Countries:*** China  ***Centers:*** Clinical Center of Acute Pancreatitis, Capital Medical University, Beijing, China, and Department of General Surgery, Xuanwu Hospital, Capital Medical University, Beijing, China  ***Setting:*** Patients with acute necrotizing pancreatitis  ***Funding sources*:** Supported by Beijing Municipal Science and Technology Commission, Beijing Municipal Science and Technology Commission Clinical Diagnosis and Treatment Technology Research ***Dropout rates:*** Not applicable  ***Limitations*:** Retrospective design, small sample size | 98 patients with acute necrotizing pancreatitis | Early intervention (step-up surgical necrosectomy) in patients with acute necrotizing pancreatitis. Patients were divided into an early group (n= 43) and a delayed group (n = 55) according to the ﬁrst percutaneous drainage (PCD) intervention time (≤ 4 weeks or > 4 weeks) |
| **Outcome Measurement/results** | Persistent organ failure (POF), number of surgical interventions, mortality, postoperative complications, length of hospital stay, operation costs, overall survival | After the minimally invasive intervention, the body temperature and inﬂammatory factors of the two groups decreased signiﬁcantly, most patients with POF improved, and the number of patients with reversal of POF in the early group was higher than that in the delayed group. Although the patients in the early group required more surgical intervention than those in the delayed group, there was no signiﬁcant difference in mortality, incidence of postoperative complications, total length of hospital stay, or operation cost between the two groups. During long-term follow-up, there was no signiﬁcant difference in the incidence of short-term and long-term complications and overall survival between the two groups | |
| **Conclusions** | Compared to patients in the delayed group, the early intervention did not affect the prognosis of patients with acute necrotizing pancreatitis. It may be more suitable for patients with pancreatic necrosis with deterioration [such as POF or infected pancreatic necrosis (IPN)] | | |

| **Paper:** Safety and Efficacy of Early (<4 Weeks of Illness) Endoscopic Transmural Drainage of Post-acute Pancreatic Necrosis Predominantly Located in the Body of the Pancreas  Journal of gastrointestinal surgery 2021  Rana SS, *et al.* | | | |
| --- | --- | --- | --- |
| **Study type/ evidence level** | **Study details/limitations** | **Patients’ characteristics** | **Interventions** |
| **Study type:** Retrospective cohort study  **Evidence level:** Low | ***Countries:*** India  ***Centers:*** Department of Gastroenterology, Postgraduate Institute of Medical Education and Research (PGIMER), Chandigarh, India  ***Setting:*** Patients with necrotizing pancreatitis  ***Funding sources*:** Not reported  ***Dropout rates:*** Not applicable  ***Limitations*:** Retrospective study design (selection bias), small sample size (imprecision) | 170 patients with pancreatic necrotic collections (PNC) | Early (<4 weeks) endoscopic transluminal drainage in patients with symptomatic PNC. The outcomes and complications were compared with patients with PNC who underwent delayed endoscopic drainage (≥ 4 weeks of the onset of acute necrotizing pancreatitis) |
| **Outcome Measurement/results** | Clinical success, mortality, need for rescue surgical necrosectomy, bleeding | Thirty-four patients (26 males; mean age: 35.9 ± 8.6 years) underwent early and 136 patients (115 males; mean age: 37.9 ± 9.4 years) underwent delayed endoscopic drainage. The PNC was significantly larger (12.3 ± 2.1 cm vs 10.5 ± 2.7 cm, p< 0.001) with increased solid component (47.7 ± 8.9% vs. 28.3 ± 11.7%, p< 0.001) in the early group. Clinical success was achieved in 94% of patients in the early group and all patients in the delayed group. Direct endoscopic necrosectomy was performed more frequently in the early group (50% vs. 7.4%; p< 0.001). There was increased mortality (5.7% vs. 0%), need for rescue surgical necrosectomy (5.7% vs. 0%), and clinically significant bleeding (20% vs. 1.5%, p<0.001) in the early group as compared to the delayed group | |
| **Conclusions** | Early endoscopic drainage of pancreatic necrotic collections is feasible and seems to be safe as well as effective but it is associated with an increased risk of complications as compared to delayed drainage | | |

| **Paper:** Immediate versus Postponed Intervention for Infected Necrotizing Pancreatitis  The New England Journal of Medicine 2021  Boxhoorn L, *et al.* | | | |
| --- | --- | --- | --- |
| **Study type/ evidence level** | **Study details/limitations** | **Patients’ characteristics** | **Interventions** |
| **Study type:** Randomized controlled trial  **Evidence level:** High | ***Countries:*** the Netherlands  ***Centers:*** Multicentric (the Dutch Pancreatitis Study Group)  ***Setting:*** Patients with infected necrotizing pancreatitis  ***Funding sources*:** Supported by Fonds NutsOhra, the Netherlands, and the Amsterdam UMC, University of Amsterdam  ***Dropout rates:*** 0%  ***Limitations*:** the trial protocol allowed for both endoscopic and surgical step-up approaches | 104 patients with infected pancreatic necrosis | Patients were randomly assigned to immediate drainage (55 patients) or postponed drainage (49 patients) |
| **Outcome Measurement/results** | The primary end point was the score on the Comprehensive Complication Index, which incorporates all complications over the course of 6 months of follow-up | The mean score on the Comprehensive Complication Index (scores range from 0 to 100, with higher scores indicating more severe complications) was 57 in the immediate-drainage group and 58 in the postponed-drainage group (mean difference, −1; 95% confidence interval [CI], −12 to 10; P= 0.90). Mortality was 13% in the immediate-drainage group and 10% in the postponed-drainage group (relative risk, 1.25; 95% CI, 0.42 to 3.68). The mean number of interventions (catheter drainage and necrosectomy) was 4.4 in the immediate-drainage group and 2.6 in the postponed-drainage group (mean difference, 1.8; 95% CI, 0.6 to 3.0). In the postponed-drainage group, 19 patients (39%) were treated conservatively with antibiotics and did not require drainage; 17 patients survived. The incidence of adverse events was similar in the two groups | |
| **Conclusions** | This trial did not show the superiority of immediate drainage over postponed drainage with regard to complications in patients with infected necrotizing pancreatitis. Patients randomly assigned to the postponed-drainage strategy received fewer invasive interventions | | |

| **Paper:** Early (<4 Weeks) Versus Standard (≥ 4 Weeks) Endoscopically centered Step-up Interventions for necrotizing Pancreatitis  American Journal of Gastroenterology 2018  Trikudanathan G, *et al.* | | | |
| --- | --- | --- | --- |
| **Study type/ evidence level** | **Study details/limitations** | **Patients’ characteristics** | **Interventions** |
| **Study type:** Retrospective analysis of a prospectively maintained database  **Evidence level:** Moderate | ***Countries:*** USA  ***Centers:*** University of Minnesota, Minneapolis, Mn, USA  ***Setting:*** Patients with necrotizing pancreatitis  ***Funding sources*:** None  ***Dropout rates:*** Not applicable  ***Limitations*:** Non-randomized study design (selection bias) | 305 patients with acute necrotizing pancreatitis (NP) | Of 305 patients with collections associated with NP, 193 (63%) (median age-52 years) required intervention performed by a step-up approach. Of the 193 patients, 76 patients underwent early and 117 patients standard intervention (<4 weeks or ≥ 4 weeks from the onset of pancreatitis) |
| **Outcome Measurement/results** | Organ failure, mortality, need for rescue open necrosectomy, hospital stay, ICU stay | Compared with standard intervention, early intervention was more often performed for infection (91% vs. 39%, p< 0.05), more associated with acute kidney injury (43% vs.32%, p= 0.09), respiratory failure (41% vs. 22%, p= 0.005), and shock (13% vs. 4%, p< 0.05). Organ failure improved significantly after intervention in both groups. There was a significant difference in mortality (13% vs. 4%, p= 0.02) and need for rescue open necrosectomy (7% vs. 1%, p= 0.03) between groups. Patients undergoing early intervention had increased median hospital (37 days vs. 26 days, p= 0.01) and ICU stay (median 2.5 days vs. 0 days, p= 0.001). There was no difference in complications | |
| **Conclusions** | When using an endoscopically centered step-up strategy in necrotizing pancreatitis, early (<4 weeks) interventions were more often performed for infection and organ failure, with no increase in complications, a similar improvement in organ failure, slightly increased need for surgery, and relatively low mortality. Early endoscopic drainage ± necrosectomy should be considered when there is a strong indication for intervention | | |

**13. Supplemental Files Table 12.** Supplementary Table Report Template Question 10.

**Research question 10. *Should early laparoscopic cholecystectomy during index admission (or within 14 days) vs. delayed laparoscopic cholecystectomy after hospital discharge be used for patients with mild acute biliary pancreatitis?***

| **Paper:** Meta-analysis of randomized clinical trials of early versus delayed cholecystectomy for mild gallstone pancreatitis  British Journal of Surgery 2019  Moody N, *et al.* | | | |
| --- | --- | --- | --- |
| **Study type/ evidence level** | **Study details/limitations** | **Patients’ characteristics** | **Interventions** |
| **Study type:** Systematic review and meta-analysis of randomized controlled trials  **Evidence level:** High | ***Countries:*** Not applicable  ***Centers:*** Not applicable  ***Setting:*** Patients with mild gallstone pancreatitis  ***Funding sources*:** Funded by a National Institute for Health Research Academic Clinical Fellowship  ***Dropout rates:*** Not applicable  ***Limitations*:** The primary and secondary endpoints differed between studies | 629 patients with mild gallstone pancreatitis | Patients were randomized as follows: 318 in the early cholecystectomy (EC) group and 311 in the delayed cholecystectomy (DC) group.  EC was defined as cholecystectomy on index admission and DC as interval cholecystectomy performed at least 2 weeks after discharge following an initial admission with mild gallstone pancreatitis |
| **Outcome Measurement/results** | Recurrent biliary complications causing hospital readmission (primary outcomes), intraoperative and postoperative complications, and total length of hospital stay (LOS) (secondary outcomes) | Recurrent biliary events that required readmission were reduced in patients undergoing EC compared with the number of patients having DC (odds ratio (OR) 0.17, 95% 0.09 to 0.33). There was no difference in the rate of intraoperative (OR 0⋅58, 0.17 to 1.92) or postoperative (OR 0.78, 0.38 to 1.62) complications | |
| **Conclusions** | Early cholecystectomy following mild gallstone pancreatitis does not increase the risk of intraoperative or postoperative complications, but reduces the readmission rate for recurrent biliary complications | | |

| **Paper:** The optimal timing of laparoscopic cholecystectomy in patients with mild gallstone pancreatitis. A meta-analysis  Medicine 2019  Zhong FP, *et al.* | | | |
| --- | --- | --- | --- |
| **Study type/ evidence level** | **Study details/limitations** | **Patients’ characteristics** | **Interventions** |
| **Study type:** Systematic review and meta-analysis of randomized and non-randomized studies  **Evidence level:** Moderate | ***Countries:*** Not applicable  ***Centers:*** Not applicable  ***Setting:*** Patients with mild gallstone pancreatitis  ***Funding sources*:** None  ***Dropout rates:*** Not applicable  ***Limitations*:** Non-randomized studies were included | 2639 patients with mild gallstone pancreatitis | Early laparoscopic cholecystectomy (ELC) and delayed laparoscopic cholecystectomy (DLC). ELC was defined as the same admission laparoscopic cholecystectomy, whereas in DLC group, cholecystectomy was performed within 2 weeks after admission |
| **Outcome Measurement/results** | Intraoperative complications, postoperative complications, rate of conversion to open cholecystectomy, operative time, rate of hospital readmission, length of hospital stay, gallstone-related events | There was no signiﬁcant difference in intraoperative complications [risk ratio (RR)=1.46; 95% conﬁdence interval (CI)=0.88–2.41; P= 0.14)], postoperative complications (RR=0.81; 95% CI=0.58–1.14; P= 0.23), rate of conversion to open cholecystectomy (RR=1.00; 95% CI=0.75–1.33; P= 0.99), operative time (MD=1.60; 95% CI= -1.36–4.56; P= 0.29), and rate of readmission (RR=0.63; 95% CI= 0.19–2.10; P= 0.45) between the ELC and DLC groups. However, the ELC group was signiﬁcantly correlated with lower length of hospital stay (MD= -2.01; 95% CI= -3.15 to -0.87; P= 0.0006), fewer gallstone-related events rates (RR=0.17; 95% CI=0.07–0.44; P= 0.0003), and lower endoscopic retrograde cholangiopancreatography (ERCP) usage (RR=0.83; 95% CI=0.71–0.97; P= 0.02) compared with the DLC group | |
| **Conclusions** | Early laparoscopic cholecystectomy is safe and effective for patients with mild acute gallstone pancreatitis | | |

| **Paper:** Early Versus Delayed Cholecystectomy for Acute Biliary Pancreatitis: A Systematic Review and Meta-Analysis  World Journal of Surgery 2022  Prasanth J, *et al.* | | | |
| --- | --- | --- | --- |
| **Study type/ evidence level** | **Study details/limitations** | **Patients’ characteristics** | **Interventions** |
| **Study type:** Systematic review and meta-analysis of randomized controlled trials  **Evidence level:** High | ***Countries:*** Not applicable  ***Centers:*** Not applicable  ***Setting:*** Patients with acute biliary pancreatitis  ***Funding sources*:** None  ***Dropout rates:*** Not applicable  ***Limitations*:** Signiﬁcant heterogeneity in the deﬁnition of early and delayed cholecystectomy and the deﬁnition of the severity of pancreatitis in each RCT, lack of high-quality data on patients with moderately-severe and severe pancreatitis | 1176 patients with acute biliary pancreatitis (mostly of mild severity) | Early Cholecystectomy (EC) (deﬁned as cholecystectomy within the same admission) versus  Delayed Cholecystectomy (DC) (deﬁned as cholecystectomy done after the resolution of symptoms of pancreatitis or after a speciﬁc time interval (after 48 h or 6 weeks) |
| **Outcome Measurement/results** | Recurrent biliary events, recurrent pancreatitis | High-quality evidence from seven RCTs (867 participants) showed a statistically signiﬁcant reduction in the risk for recurrent biliary events in favor of early cholecystectomy (RR 0.10, 95% CI 0.05 to 0.19, I2= 0%). High-quality evidence from ﬁve trials was in favor of early cholecystectomy with a signiﬁcant reduction in the risk of recurrent pancreatitis (RAP) in comparison to delayed cholecystectomy (RR 0.21, 95% CI 0.09 to 0.51, I2= 0%) | |
| **Conclusions** | Early cholecystectomy has deﬁnite advantages over delayed cholecystectomy in terms of reducing recurrent pancreaticobiliary events following mild acute biliary pancreatitis | | |

| **Paper:** Timing of Laparoscopic Cholecystectomy After Mild Biliary Pancreatitis: A Systematic Review and Meta-Analysis  Journal of laparoendoscopic & advanced surgical techniques 2017  Yang DJ, *et al.* | | | |
| --- | --- | --- | --- |
| **Study type/ evidence level** | **Study details/limitations** | **Patients’ characteristics** | **Interventions** |
| **Study type:** Systematic review and meta-analysis of randomized controlled trials and non-randomized studies  **Evidence level:** Moderate | ***Countries:*** Not applicable  ***Centers:*** Not applicable  ***Setting:*** Patients with mild biliary pancreatitis  ***Funding sources*:** Not reported  ***Dropout rates:*** Not applicable  ***Limitations*:** The precise timing of early cholecystectomy was variable. The included studies were highly heterogeneous because of their different study designs (inconsistency). The pooled analysis only included three randomized trials (imprecision) | 2291 patients with mild biliary pancreatitis | 1141 (49.8%) patients underwent early laparoscopic cholecystectomy (ELC), and 1150 (50.2%) underwent delayed laparoscopic cholecystectomy (DLC). ELC was defined as initial cholecystectomy or laparoscopic cholecystectomy performed within 14 days after admission. The control group was deﬁned as DLC (from 3 days to 12 weeks) |
| **Outcome Measurement/results** | Complications, readmission, length of hospital stay, rates of conversion to open cholecystectomy, duration of surgery | The included studies described 2291 patients, of whom 1141 (49.8%) underwent ELC and 1150 (50.2%) underwent DLC. The reported rate of complications for ELC (6.8%) was lower than that for DLC (13.45%). The reported rate of readmission for ELC was lower than that for DLC. The length of hospital stay was longer with DLC than with ELC. ELC and DLC did not have signiﬁcantly different rates of conversion to open cholecystectomy and duration of surgery | |
| **Conclusions** | ELC is better than DLC in many aspects for acute mild pancreatitis patients undergoing laparoscopic cholecystectomy. ELC associated with few complications and readmissions, as well as a short length of hospital stay | | |

| **Paper:** The safety, feasibility, and cost-effectiveness of early laparoscopic cholecystectomy for patients with mild acute biliary pancreatitis: A meta-analysis  The Surgeon 2020  Yuan X, *et al.* | | | |
| --- | --- | --- | --- |
| **Study type/ evidence level** | **Study details/limitations** | **Patients’ characteristics** | **Interventions** |
| **Study type:** Systematic review and meta-analysis of randomized controlled trials  **Evidence level:** High | ***Countries:*** Not applicable  ***Centers:*** Not applicable  ***Setting:*** Patients with mild acute biliary pancreatitis  ***Funding sources*:** None  ***Dropout rates:*** Not applicable  ***Limitations*:** Small cohorts (risk of imprecision), secondary outcomes with high heterogeneity | 439 patients with mild acute biliary pancreatitis | Early laparoscopic cholecystectomy (ELC, within 72 h after admission) versus delayed laparoscopic cholecystectomy (DLC, beyond 72 h after admission) for patients with mild acute biliary pancreatitis |
| **Outcome Measurement/results** | Complication rate, rate of conversion to open cholecystectomy, rate of recurrence of acute pancreatitis, length of hospital stay | A total of 4 studies involving 439 (215 vs. 224) patients were included. The difference in complication rate [3.3% vs. 3.2%; RR 1.03 (0.35, 3.01), P= 0.961] and rate of conversion to open cholecystectomy [3.8% vs. 3.3%; RR 1.13 (0.37, 3.43), P= 0.830] were insigniﬁcant between patients who underwent ELC and ones who underwent DLC. The difference in the rate of recurrence of acute pancreatitis was signiﬁcant between ELC and DLC (2.17% vs. 8.99%; RR 0.24 (0.08-0.70), P= 0.009). ELC did not shorten the length of hospital stay (random-effects model analysis: WMD -1.09 days (-2.67, 0.48), P= 0.173; ﬁxed-effect model analysis: WMD -0.62 days (-1.00, -0.24), P= 0.001) | |
| **Conclusions** | Compared to DLC, ELC is equally safe and feasible both in complication rate and rate of conversion to open procedure, and signiﬁcantly reduces the recurrence rate of acute pancreatitis | | |

| **Paper:** Cholecystectomy 7 days vs 4 weeks after mild biliary pancreatitis; looking a decrease the incidence of persistent choledocholithiasis and ERCP: A multicentric randomized clinical trial  International journal of surgery 2022  Facundo Gomez H, *et al.* | | | |
| --- | --- | --- | --- |
| **Study type/ evidence level** | **Study details/limitations** | **Patients’ characteristics** | **Interventions** |
| **Study type:** Randomized controlled trial  **Evidence level:** High | ***Countries:*** Spain  ***Centers:*** Multicentric (Moises Broggi Hospital, Barcelona, Coordinating Center)  ***Setting:*** Patients with mild acute biliary pancreatitis  ***Funding sources*:** Catalan Surgical Society  ***Dropout rates:*** 12.5% (early cholecystectomy) and 13.8% (delayed cholecystectomy)  ***Limitations*:** - | 198 patients with a first episode of mild acute biliary pancreatitis (MABP) defined by the Atlanta 2012 criteria | 98 patients were randomized to early surgery (7 days) and 100 to delayed surgery (4 weeks) |
| **Outcome Measurement/results** | Readmission rates for recurrent biliary events and the incidence of residual choledocholithiasis, postoperative hospital stay, complications | Early surgery reduced the rate of readmissions for biliary events before cholecystectomy by half (7.2% vs. 15.8%, p= 0.058). There were no differences in the type of surgery, postoperative stay, or complications compared with delayed surgery. Choledocholithiasis was observed in 9.0% of patients in the early group and 7.7% in the delayed group (p= 0.719). The preoperative or intraoperative imaging study avoided unnecessary ERCP, which was performed in only 6 (3%) patients | |
| **Conclusions** | Early cholecystectomy performed seven days after resolution of mild acute biliary pancreatitis had a low incidence of recurrent biliary events and complications and was not associated with an increase in residual choledocholithiasis or need for unnecessary ERCP | | |

| **Paper:** Early Cholecystectomy Safely Decreases Hospital Stay in Patients With Mild Gallstone Pancreatitis. A Randomized Prospective Study  Annals of surgery 2010  Aboulian A, *et al.* | | | |
| --- | --- | --- | --- |
| **Study type/ evidence level** | **Study details/limitations** | **Patients’ characteristics** | **Interventions** |
| **Study type:** Randomized controlled trial  **Evidence level:** High | ***Countries:*** California (USA)  ***Centers:*** Departments of Surgery and Emergency Medicine, Harbor UCLA Medical Center, Torrance, CA; and Los Angeles Biomedical Research Institute, Los Angeles, CA  ***Setting:*** Patients with mild gallstone pancreatitis  ***Funding sources*:** Not reported  ***Dropout rates:*** 0%  ***Limitations*:** The study design was powered to detect a difference in length of stay and not sufﬁciently powered to detect a difference in the secondary endpoint | 50 patients with mild gallstone pancreatitis (Ranson score ≤ 3) | Patients were prospectively randomized to either an early laparoscopic cholecystectomy group (within 48 hours of admission) (25 patients) versus a control laparoscopic cholecystectomy group (performed after resolution of abdominal pain and normalizing trend of laboratory enzymes) (25 patients) |
| **Outcome Measurement/results** | The primary end point was hospital length of stay. Secondary end point was a composite of rates of conversion to an open procedure, perioperative complications, and need for endoscopic retrograde cholangiography | The hospital length of stay was shorter for the early cholecystectomy group (mean: 3.5 [95% CI, 2.7–4.3], median: 3 [IQR, 2–4]) compared with the control group (mean: 5.8 [95% CI, 3.8–7.9], median: 4 [IQR, 4–6] P= 0.0016). Six patients from the early group required endoscopic retrograde cholangiography, compared with 4 in the control group (P= 0.72). There was no statistically signiﬁcant difference in the need for conversion to an open procedure or in perioperative complication rates between the 2 groups | |
| **Conclusions** | In mild gallstone pancreatitis, laparoscopic cholecystectomy performed within 48 hours of admission, regardless of the resolution of abdominal pain or laboratory abnormalities, results in a shorter hospital length of stay with no apparent impact on the technical difﬁculty of the procedure or perioperative complication rate | | |

| **Paper:** Impact of Early Cholecystectomy on the Cost of Treating Mild Gallstone Pancreatitis: Gallstone PANC Trial  Journal of the American College of Surgeons 2021  Isbell KD, *et al.* | | | |
| --- | --- | --- | --- |
| **Study type/ evidence level** | **Study details/limitations** | **Patients’ characteristics** | **Interventions** |
| **Study type:** Randomized controlled trial  **Evidence level:** High | ***Countries:*** Texas (USA)  ***Centers:*** Department of Surgery and Center for Surgical Trials and Evidence-based Practice (CSTEP), the Center for Clinical Research and Evidence-Based Medicine, McGovern Medical School at the University of Texas Health Science Center, Houston, TX, and the University of Houston, HCA Healthcare Kingwood, Kingwood, TX, USA  ***Setting:*** Patients with mild gallstone pancreatitis  ***Funding sources*:** National Institute of General Medical Sciences  ***Dropout rates:*** 1.02%  ***Limitations*:** Small sample size, uncertain generalizability | 97 patients with mild gallstone pancreatitis | Patients were randomized in early (cholecystectomy within 24 hours of admission) (early) (n= 49) and control (n= 48) (cholecystectomy after clinical resolution) groups |
| **Outcome Measurement/results** | Costs for index admissions and all gallstone pancreatitis-related care 90 days post-discharge | Early cholecystectomy resulted in a mean absolute difference in LOS of -0.96 days (95% CI, -1.91 to 0.00, p= 0.05). Ninety-day mean total costs were $14,974 (early) vs. $16,190 (control) (cost ratio [CR], 0.92; 95% CI, 0.73-1.15, p= 0.47), with a mean absolute difference of $1,216 less (95% CI, -$4,782 to $2,349, p= 0.50) per patient in the early group. On Bayesian analysis, there was an 81% posterior probability that early cholecystectomy reduced 90-day total costs | |
| **Conclusions** | In this single-center trial, early cholecystectomy for mild gallstone pancreatitis reduced 90-day length of hospital stay and had an 81% probability of reducing 90-day healthcare system costs | | |

| **Paper:** Index versus delayed cholecystectomy in mild gallstone pancreatitis: results of a randomized controlled trial  HPB 2018  Noel R, *et al.* | | | |
| --- | --- | --- | --- |
| **Study type/ evidence level** | **Study details/limitations** | **Patients’ characteristics** | **Interventions** |
| **Study type:** Randomized controlled trial  **Evidence level:** High | ***Countries:*** Sweden  ***Centers:*** Surgical department at the Karolinska University Hospital, Sweden  ***Setting:*** Patients with mild gallstone pancreatitis  ***Funding sources*:** Not reported  ***Dropout rates:*** 3.1% in the index group and 14.7% in the delayed group  ***Limitations*:** Single-center design | 66 patients with mild gallstone pancreatitis | Patients with mild gallstone pancreatitis were randomized into index–or delayed cholecystectomy (IC vs. DC). IC was performed within 48 hours from randomization, providing a stable or improved clinical condition. Patients randomized to DC were scheduled for cholecystectomy at least 6 weeks after primary discharge |
| **Outcome Measurement/results** | The primary outcome was gallstone-related events. Secondary outcomes were rates of cholecystectomy complications, common bile duct stones (CBDS) detected at cholecystectomy and patient reported quality-of-life and pain | There were signiﬁcantly higher rates of gallstone-related events in the DC compared with the IC group (nine patients vs. one patient, p= 0.013). No statistically signiﬁcant differences could be demonstrated in cholecystectomy complications (p= 0.605) and CBDS discovered during cholecystectomy (p = 0.302) between the groups. Pain and emotional well-being measured by SF-36 were improved signiﬁcantly in the IC group at follow-up | |
| **Conclusions** | Delayed cholecystectomy in mild gallstone pancreatitis can no longer be recommended since it is associated with an increased risk for recurrent gallstone-related events and impaired patient’s reported outcomes | | |

**14. Supplemental Files Figure 1.** GRADE Assessment PICO 1. Should routine enhanced CT scan at the time of hospital admission vs. contrast-enhanced CT performed 72-96 hours after onset of symptoms be used for the diagnosis of local complications (fluid collections, pancreatic necrosis) in patients with severe acute biliary pancreatitis?


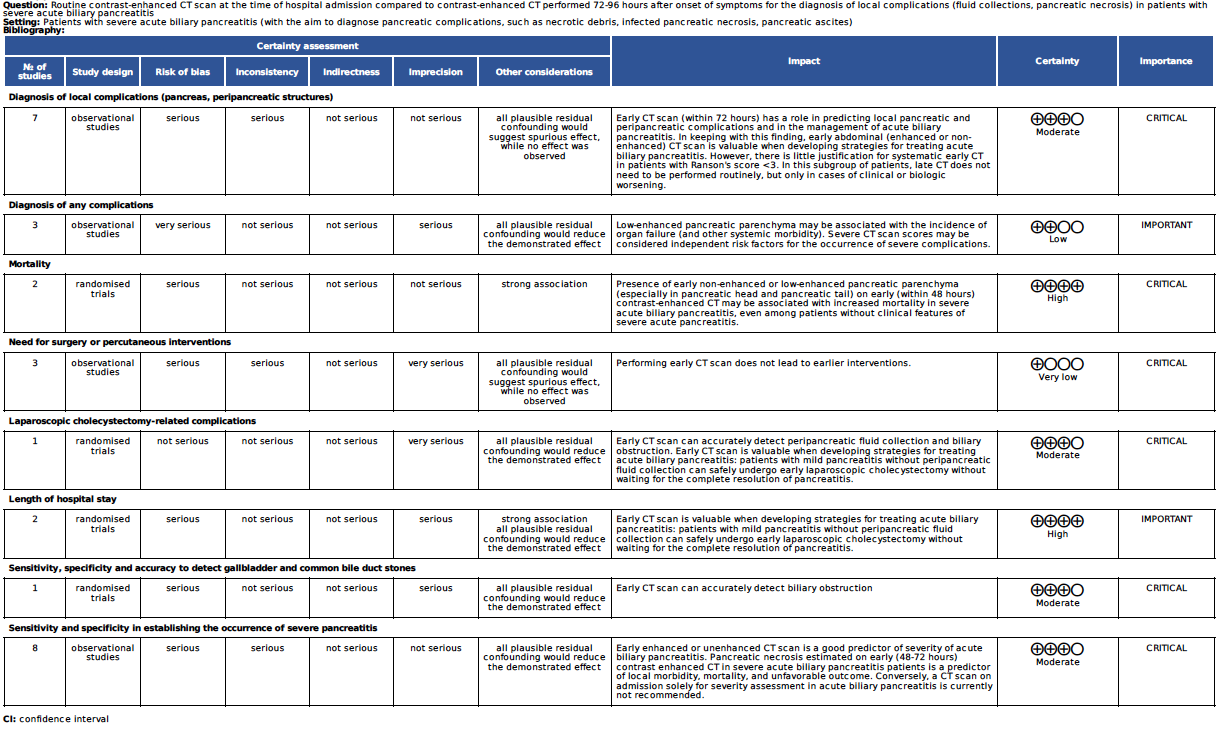


**15. Supplemental Files Figure 2.** GRADE Assessment PICO 2. Should routine prophylactic antibiotics vs. no routine prophylactic antibiotics be used for patients with acute biliary pancreatitis in the absence of infectious complications?


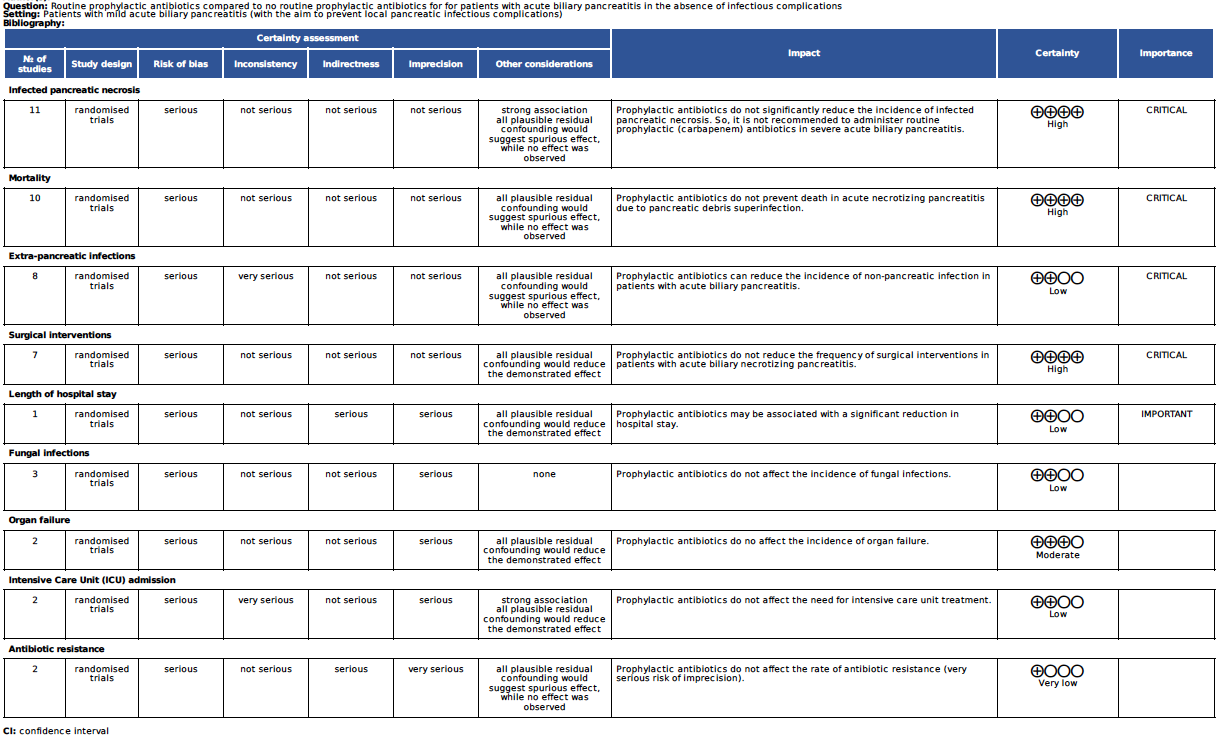


**16. Supplemental Files Figure 3.** GRADE Assessment PICO 3. Should serum measurements of procalcitonin (PCT) vs. other sepsis markers be used for the early diagnosis of infected pancreatic necrosis in patients with severe acute biliary pancreatitis?


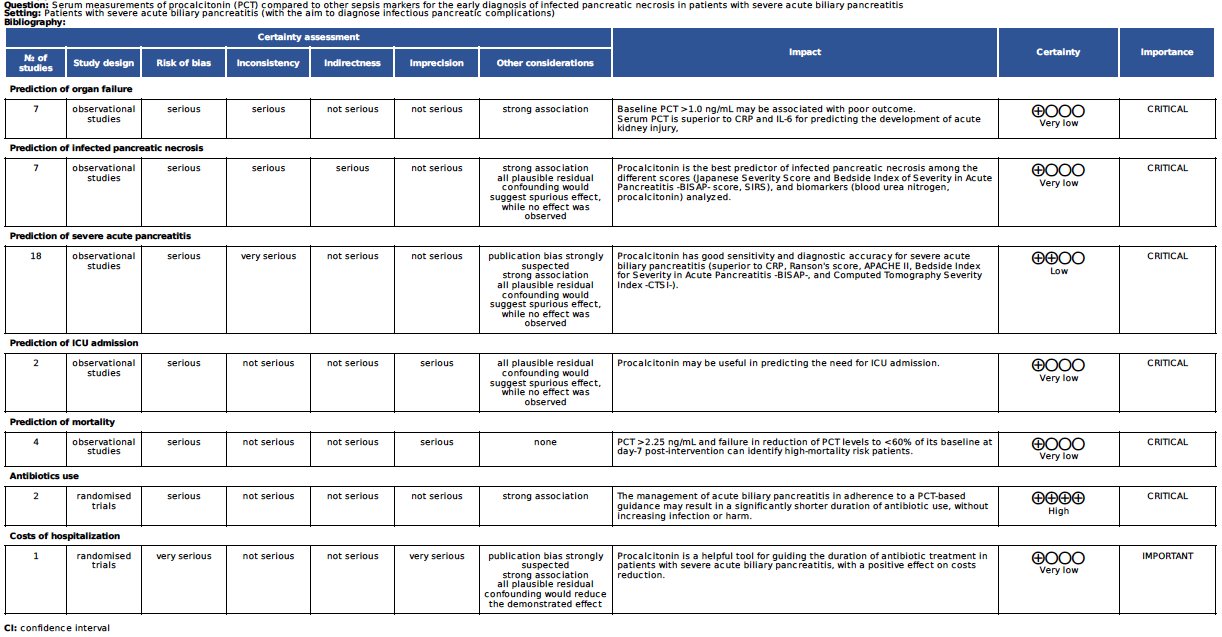


**17. Supplemental Files Figure 4.** GRADE Assessment PICO 4. Should early (within 24 hours) oral feeding as tolerated vs. keeping the patient nil per os be used for patients with mild acute biliary pancreatitis (if tolerated)?


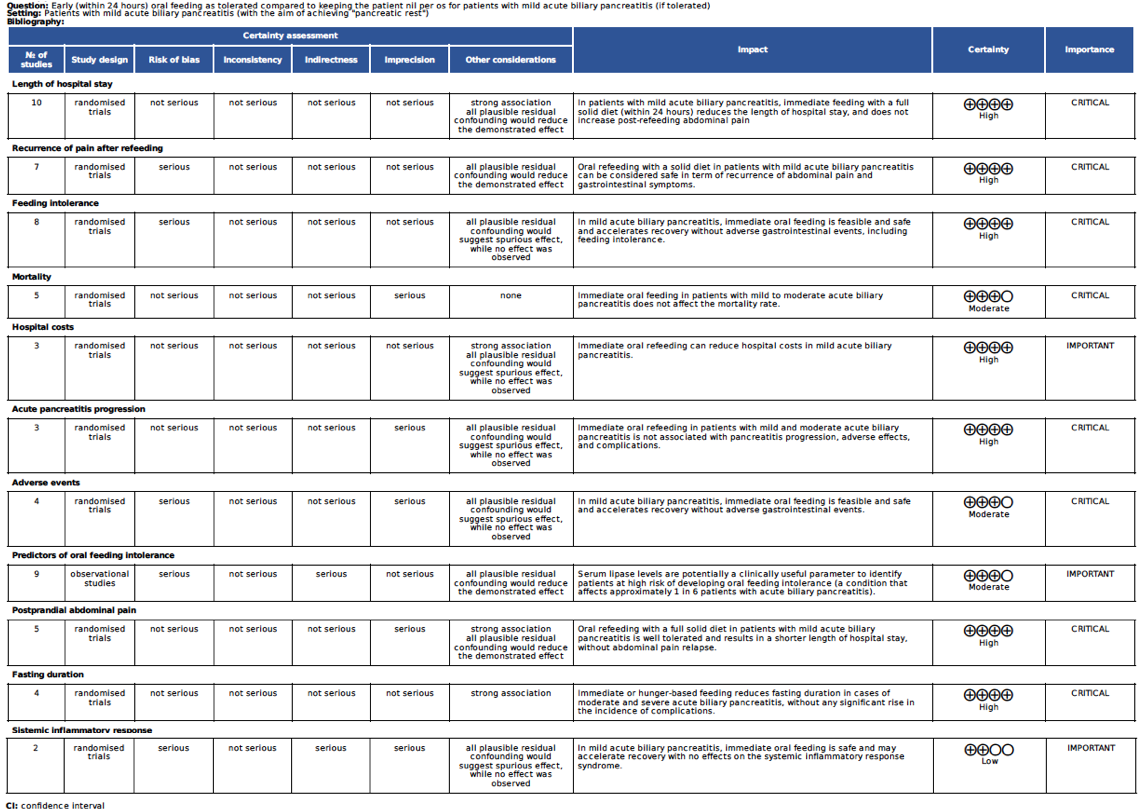


**18. Supplemental Files Figure 5.** GRADE Assessment PICO 5. Should enteral nutrition (EN) vs. total parenteral nutrition (TPN) be used for the prevention of gut failure and infectious complications in patients with acute biliary pancreatitis and the inability to feed orally?


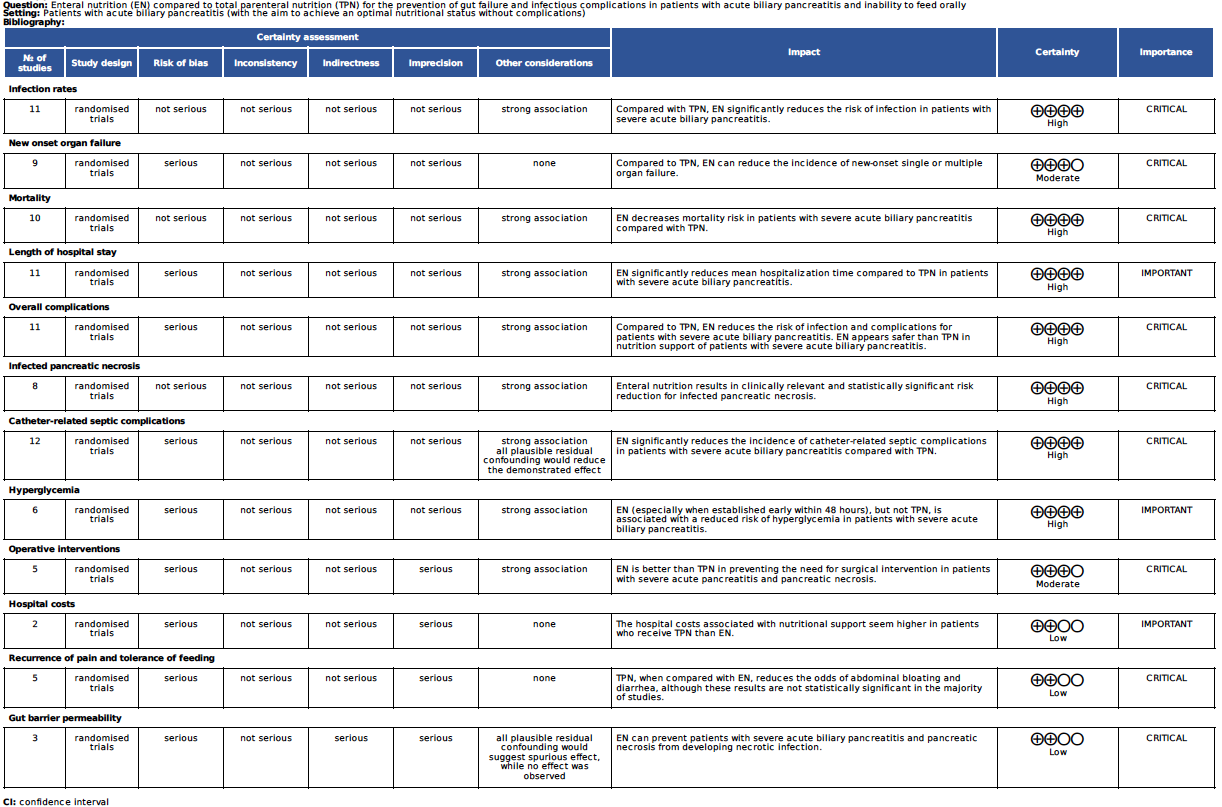


**19. Supplemental Files Figure 6.** GRADE Assessment PICO 6. Should early enteral nutrition (eEN) within 48 hours vs. delayed enteral nutrition (dEN) beyond 48 hours be used in patients with severe acute biliary pancreatitis and inability to feed orally?


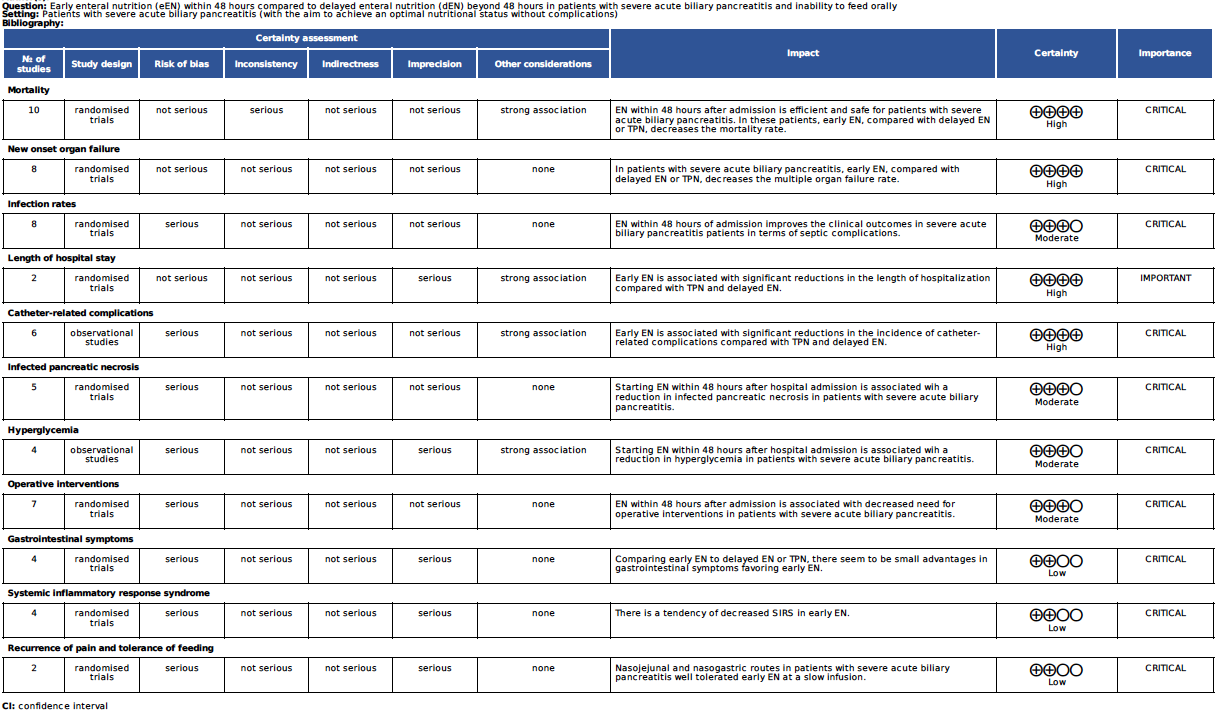


**20. Supplemental Files Figure 7.** GRADE Assessment PICO 7. Should early (within 48-72 hours) ERCP/ES vs. delayed (> 72 hours) or conservative treatment be used in gallstone-induced acute biliary pancreatitis when cholangitis and/or common bile duct obstruction occur?


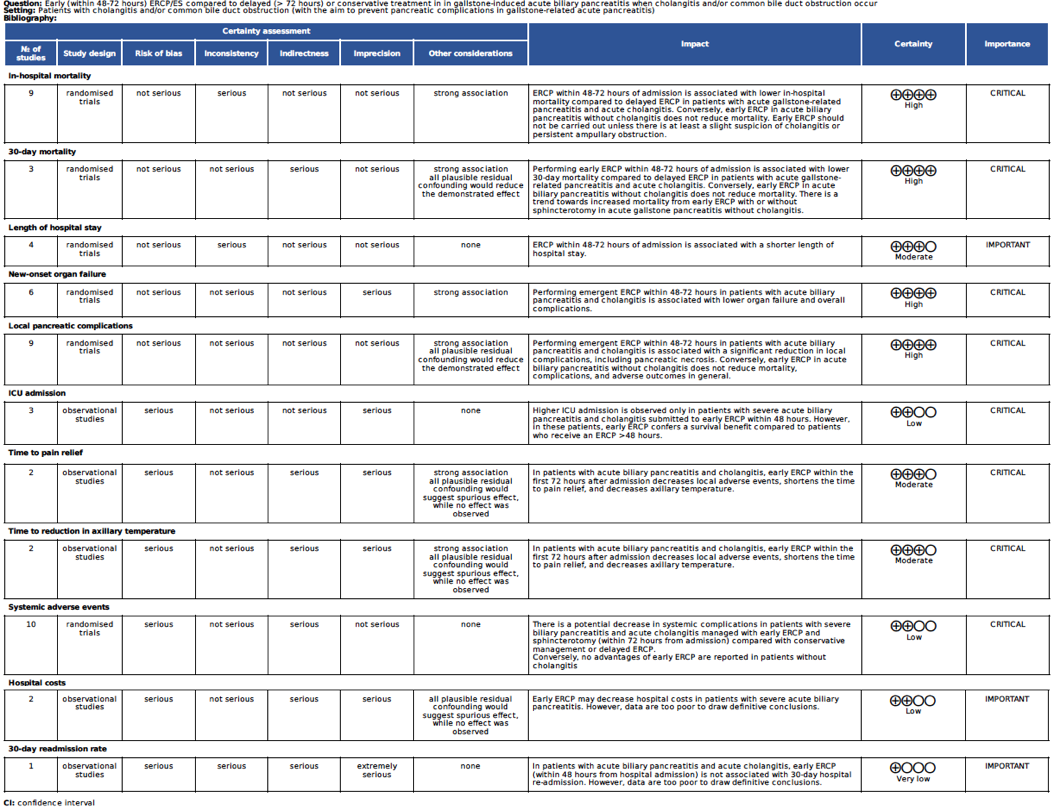


**21. Supplemental Files Figure 8.** GRADE Assessment PICO 8. Should surgical or endoscopic step-up approach vs. upfront necrosectomy be used as the first line of treatment for patients with pancreatic necrosis?


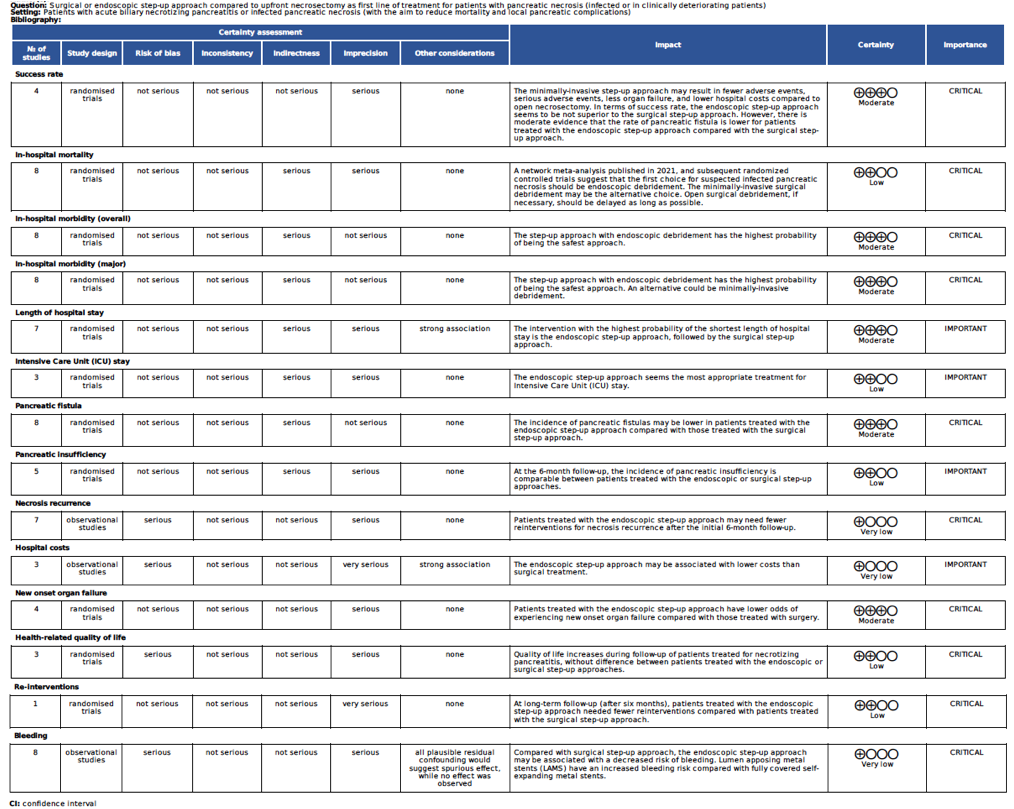


**22. Supplemental Files Figure 9.** GRADE Assessment PICO 9. Should delayed (after 4 weeks) therapeutic interventions (endoscopic or surgical step-up approach, surgical necrosectomy) vs. early interventions be used for patients with acute biliary necrotizing pancreatitis who remain clinically stable?


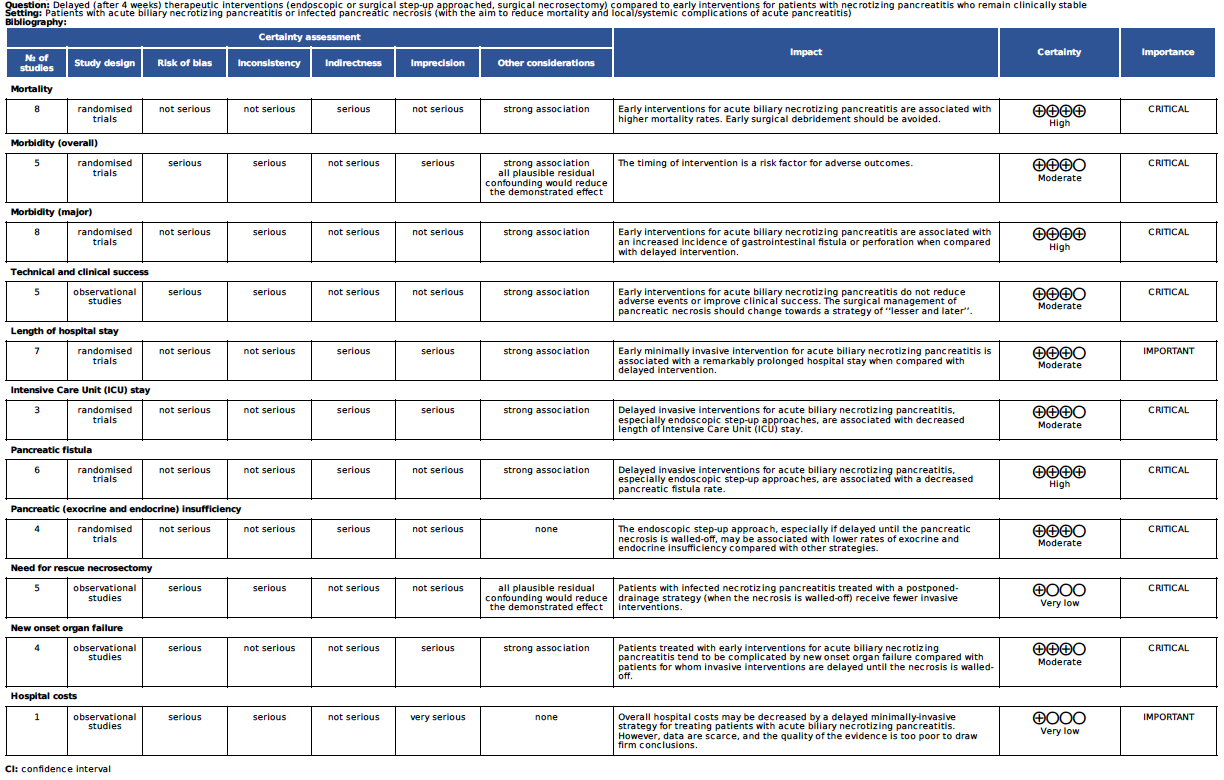


**23. Supplemental Files Figure 10.** GRADE Assessment PICO 10. Should early laparoscopic cholecystectomy during index admission (or within 14 days) vs. delayed laparoscopic cholecystectomy after hospital discharge be used for patients with mild acute biliary pancreatitis?


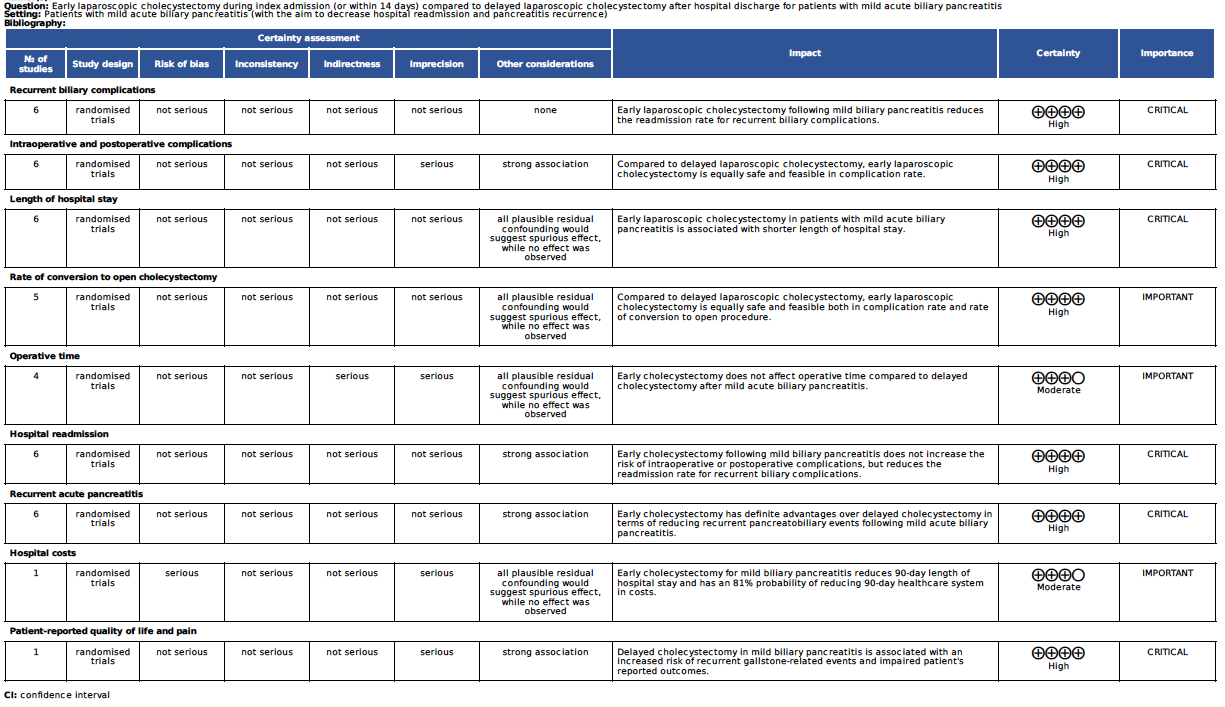

Supplement: SUPPLEMENTARY MATERIAL [file sla-279-203-s001.docx]
